# Behavioral Determinants and Effectiveness of Digital Behavior Change Interventions for the Prevention of Sexually Transmitted Infections and HIV: Overview of Systematic Reviews

**DOI:** 10.2196/74201

**Published:** 2026-01-29

**Authors:** Giuliano Duarte-Anselmi, Susana Sanduvete-Chaves, Salvador Chacón-Moscoso, Daniel López-Arenas

**Affiliations:** 1 Facultad de Psicología Universitat de Barcelona Barcelona Spain; 2 Facultad de Ciencias Médicas Universidad de Santiago de Chile Santiago Chile; 3 Departamento de Psicología Experimental Facultad de Psicología Universidad de Sevilla Sevilla Spain; 4 Universidad Autónoma de Chile Santiago Chile

**Keywords:** behavioral change, behavioral design, sexually transmitted diseases, HIV, digital behavior change intervention (DBCI)

## Abstract

**Background:**

Unsafe sexual practices remain a major contributor to global morbidity, premature mortality, and health care burden. More than 1 million people acquire a sexually transmitted infection (STI) daily, including HIV. Although biomedical innovations such as pre-exposure prophylaxis have expanded prevention options, consistent condom use and regular HIV and STI testing remain essential behavioral strategies. Adherence to these behaviors remains uneven, underscoring the need for complementary digital and behavioral approaches. Digital behavior change interventions (DBCIs), technology-based programs designed to support health-related behavior change, offer scalable and personalized tools for safer-sex promotion. However, evidence regarding their behavioral components and effectiveness remains fragmented across systematic reviews (SRs).

**Objective:**

This study aims to synthesize and critically appraise evidence on the effectiveness of DBCIs for preventing STIs and HIV, and to identify which behavior change techniques (BCTs) and theoretical domains framework (TDF) have been used to improve safe-sex behaviors.

**Methods:**

A search was conducted in MEDLINE, Cochrane Database of SRs, Epistemonikos, and PsycINFO for all publications up to November 12, 2025, without language or date restrictions. Eligible SRs examined DBCIs targeting STI and HIV prevention or reduction of risky sexual behaviors. Two reviewers (GDA and DLA) independently screened, extracted data, and appraised methodological quality using the AMSTAR-2 tool. The reporting followed the PRIOR (Preferred Reporting Items for Overviews of Reviews) and PRISMA-S (Preferred Reporting Items for SRs and Meta-Analyses Literature Search Extension) recommendations.

**Results:**

Overall, 23 SRs, comprising 514 primary studies and 129,481 participants, met the inclusion criteria. Most interventions were SMS-based, mobile app–based, or web-delivered. Digital interventions consistently improved STI and HIV testing uptake and engagement with sexual health services. Evidence for condom use and biological outcomes was mixed. Improvements in cognitive determinants, such as HIV-related knowledge, motivation, and self-efficacy, were frequently reported. Only 4 reviews explicitly applied BCT or TDF taxonomies, identifying goal setting, feedback on behavior, and prompts and cues as commonly used techniques. Research predominantly originated from high-income settings, with limited evidence from low- and middle-income countries and minimal reporting of sex- or gender-disaggregated outcomes.

**Conclusions:**

DBCIs show promise for strengthening STI/HIV prevention, particularly by increasing testing behaviors and supporting cognitive determinants of risk reduction. However, sustained condom use and biological outcomes remain inconsistent, and reporting of behavioral mechanisms is limited. This overview is the first to integrate effectiveness evidence with a systematic, mechanism-focused mapping of BCTs and TDF constructs, providing an innovation not present in earlier reviews. Clarifying which active components of digital interventions are most consistently linked to beneficial outcomes offers concrete guidance for designing culturally tailored, theory-driven, and equity-focused digital strategies. These insights have direct implications for researchers, clinicians, and policymakers seeking to develop digital prevention programs that more effectively address behavioral determinants of STI and HIV risk.

**Trial Registration:**

PROSPERO CRD42023485887; https://www.crd.york.ac.uk/PROSPERO/view/CRD42023485887

**International Registered Report Identifier (IRRID):**

RR2-10.5867/medwave.2025.02.3020

## Introduction

Unsafe sexual practices are major contributors to global morbidity and premature mortality, representing one of the leading behavioral risk factors worldwide [1,2]. Among young people, these practices significantly increase the risk of sexually transmitted infections (STIs) including syphilis, gonorrhea, chlamydia, and HIV, as well as human papillomavirus (HPV)–related cancers [3,4]. A recent report by the World Health Organization highlights an alarming decrease in condom use among adolescents in Europe, leading to higher rates of unprotected sex and, consequently, an increased risk of STIs, particularly among adolescents from low-income families [5]. Although biomedical innovations such as pre-exposure prophylaxis (PrEP) have expanded prevention options, consistent condom use and regular HIV and STI testing remain essential behavioral strategies for reducing infection risk [6]. Together, these measures form a complementary prevention framework; yet, adherence remains uneven across populations. HIV and AIDS continues to be a leading cause of death globally, with more than 1 million people acquiring an STI daily and nearly 39 million living with HIV [7,8]. Behavioral determinants, including motivation, self-regulation, risk perception, social and cultural norms, and structural barriers, shape whether individuals engage in STI and HIV prevention behaviors, yet they are often insufficiently addressed or poorly defined in existing prevention strategies.

Given the scale of these challenges, effective prevention strategies must emphasize sociocultural and behavioral changes, such as increasing awareness, reducing stigma, and promoting safe sex practices like consistent condom use and regular STI and HIV testing [9-11]. Widespread access to the Internet and mobile phones presents a unique opportunity to leverage digital interventions as private and effective methods for improving sexual health, particularly in regions with varying levels of literacy [11-14].

Recent evidence underscores the growing use of digital technologies in HIV and STI prevention. A 2024 umbrella review found that eHealth interventions, ranging from mobile apps and websites to telemedicine and social media programs, were generally effective in supporting HIV prevention, testing, and clinical management, although the methodological quality of many reviews was low [15]. However, the Shi et al [15] review included both prevention and treatment interventions, whereas the present overview focuses exclusively on preventive strategies and their behavioral mechanisms. Evidence also suggests that the inclusion of behavior change techniques (BCTs) in digital tools enhances user engagement and intervention effectiveness [16]. Additionally, recent work has highlighted the expanding role of interactive digital tools in partner notification and sexual health engagement [17]. Together, these findings demonstrate the rapid evolution of digital health approaches and emphasize the need to systematically map their behavioral components to guide the design of effective prevention programs. However, most existing systematic reviews (SRs) and umbrella reviews describe the effects of digital interventions without examining their mechanisms of action, that is, the specific theoretical pathways and BCTs through which interventions influence prevention behaviors. Without identifying these mechanisms of action, it is difficult to understand why some digital programs succeed while others do not, and which components should be replicated or scaled.

Digital interventions can be broadly defined as health-promoting programs delivered through digital platforms such as websites, mobile apps, text messaging, or social media [18]. Within this broad category, digital behavior change interventions (DBCIs) are those that explicitly incorporate theoretical frameworks and structured BCTs to influence health-related behaviors [19]. In other words, while all DBCIs are digital interventions, not all digital interventions qualify as DBCIs. This conceptual distinction underpins our search strategy and synthesis approach, focusing on interventions that use digital delivery to achieve behavioral outcomes through identifiable active ingredients. These definitions are provided upfront to reduce conceptual ambiguity, as emphasized by recent critiques in digital behavior change research.

DBCIs offer multiple advantages over traditional prevention approaches: they can deliver tailored, interactive, and adaptive content; are cost-effective and scalable; and can integrate technological features such as automated feedback and passive sensing [20-22]. However, information alone is insufficient to drive behavior change: integrating BCTs to these digital platforms is essential for achieving meaningful health outcomes [23,24].

In this context, the theoretical domains framework (TDF) is presented as a widely used proposal to systematically identify barriers and facilitators to change specific behaviors, helping design more effective interventions in health, among other fields. It is useful to understand why people do or do not do something, and highlight factors needing intervention [25,26]. It synthesizes 33 behavior change theories into 14 core domains, such as cognitive (knowledge, skills, beliefs about capabilities and consequences), affective and emotional (emotions and reinforcement), social and environmental (social and professional role, social influences, environmental context, and resources), and beliefs and intentions (optimism, intentions, goals, memory, attention, decision processes, behavioral regulation) [27].

Given the volume of SRs assessing digital interventions for STI and HIV prevention, an overview of reviews enables synthesis and comparison across multiple bodies of evidence rather than relying on a single set of primary studies. This approach provides a broader understanding of intervention effectiveness, identifying patterns, strengths, methodological gaps, and avenues for future research [28,29]. Despite their promise, many digital interventions lack clear descriptions of the BCTs and theoretical domains they use, hindering replicability and practical translation [30,31]. Identifying the most effective BCTs, especially those that successfully promote safe sex and reduce STI and HIV transmission, is crucial for public health initiatives [32-35]. To date, no overview has systematically integrated BCTs, theoretical domains, and prevention outcomes to produce a mechanism-focused synthesis of digital interventions for STI and HIV prevention. Existing reviews also provide limited up-to-date evidence and do not incorporate studies published through 2025. Addressing this gap is essential for identifying which behavioral components drive meaningful changes in prevention behaviors and for informing the development of digital strategies that are theoretically grounded, culturally responsive, and scalable.

Accordingly, this overview aims to synthesize current evidence on the use of BCTs and the TDF in digital interventions designed to prevent STIs and HIV. By examining how these behavioral components are implemented and how they influence prevention-related outcomes, this research seeks to inform the development of more effective, theory-driven digital interventions and strengthen future public health strategies. This overview therefore provides not only an updated assessment of the evidence but also a behavioral mapping that has been largely absent from previous syntheses. It further advances the field by incorporating SRs published through 2025, which have not yet been integrated in any prior synthesis.

## Methods

### Study Design

This study is an overview of SRs and adheres to the Cochrane Handbook for SRs of Interventions [29] and the PRIOR (Preferred Reporting Items for Overviews of Reviews) statement [36]. The PRIOR checklist is reported in Multimedia Appendix 1 and the Sex and Gender Equity in Research (SAGER) guidelines [37], in Multimedia Appendix 2 (section 2). In addition, the search strategy and reporting follow the PRISMA-S (Preferred Reporting Items for Systematic Reviews and Meta-Analyses Literature Search Extension) [38] to ensure full transparency and reproducibility of search methods (see Multimedia Appendix 3).

### Protocol and Registration

The protocol for this overview was prospectively registered in PROSPERO (CRD42023485887) on December 5, 2023 (Multimedia Appendix 2 section 3), and later published in full [39]. The methods adhered to the predefined protocol and incorporated SAGER guidance where applicable. Reporting also followed PRISMA 2020 recommendations for SRs and overviews [40]. The full protocol can be downloaded from the Open Science Framework [41,42].

### Patient and Public Involvement

Neither patients nor the public were involved in designing or conducting this study. Therefore, no ethical approval was required for this overview. The analyzed data were open access.

### Eligibility Criteria

The eligibility criteria were reported in detail in our protocol [39]. The inclusion criteria for this overview were based on the population, intervention, comparison, outcome, study type (PICOS) framework (Table 1).

**Table 1:** Eligibility criteria for elements of a comprehensive search strategy.

Element	Inclusion and exclusion criteria
Population	Included: SRs^a^ that have evaluated the effect of digital behavior change interventions in any population and that have described BCTs^b^, mechanisms of action, or any behavioral model or framework that takes into account how the digital intervention influences the behavior change used to reduce the risk or prevent the transmission of STIs^b^, including HIV. Excluded: studies that did not focus on prevention or that focused on treatment and adherence to antiretroviral therapy and self-care of people living with HIV were excluded, as the focus of the question research is risk reduction and prevention of STIs and HIV.
Intervention	Included: SRs that evaluated digital and mobile health behavior change interventions focusing on modifying unsafe sexual behaviors or preventing STIs and HIV, that is, interventions carried out using a digital or mobile platform as a direct interface with the participants. Excluded: studies that did not report the use of digital intervention, that is, did not incorporate digital technology such as smartphones, computers, tablets, multimedia, and social networks.
Comparator	As this was an overview of SRs, a comparator or control group was not an inclusion criterion for this study. However, given our interest in effectiveness of interventions and BCTs, we included reviews where evidence from primary experimental studies with an appropriate comparator was available.
Outcome	Included: during the selection process, we do not consider concrete results as an inclusion criterion. All studies that assessed short- or long-term behavior change concerning the following primary outcomes were included: reduction in risky sexual behaviors, such as condom use (last sexual encounter, frequency, consistency) and increased STI and HIV testing; and prevention (vaccination against HPV^d^ and hepatitis A and B, and HIV pre-exposure prophylaxis). As secondary outcomes, the use of behavior change theories or techniques was analyzed, using standardized classifications if available, such as the taxonomy of BCTs. Excluded: any study that did not include a primary and/or secondary outcome.
Study design	Included: SRs only Excluded: studies other than SRs (eg, primary studies, commentary articles, and conferences) were excluded.

^a^SR: systematic review.

^b^BCT: behavior change technique.

^c^STI: sexually transmitted infection.

^d^HPV: human papillomavirus.

Summary of inclusion and exclusion criteria used to identify SRs of digital behavior change interventions for the prevention of STIs and HIV. Criteria were defined following the Cochrane Handbook and the PRIOR guideline. All SRs were operationally defined as secondary research including primary clinical studies with explicit search strategies in ≥2 databases [29,43].

### Information Sources

**Figure 1:**
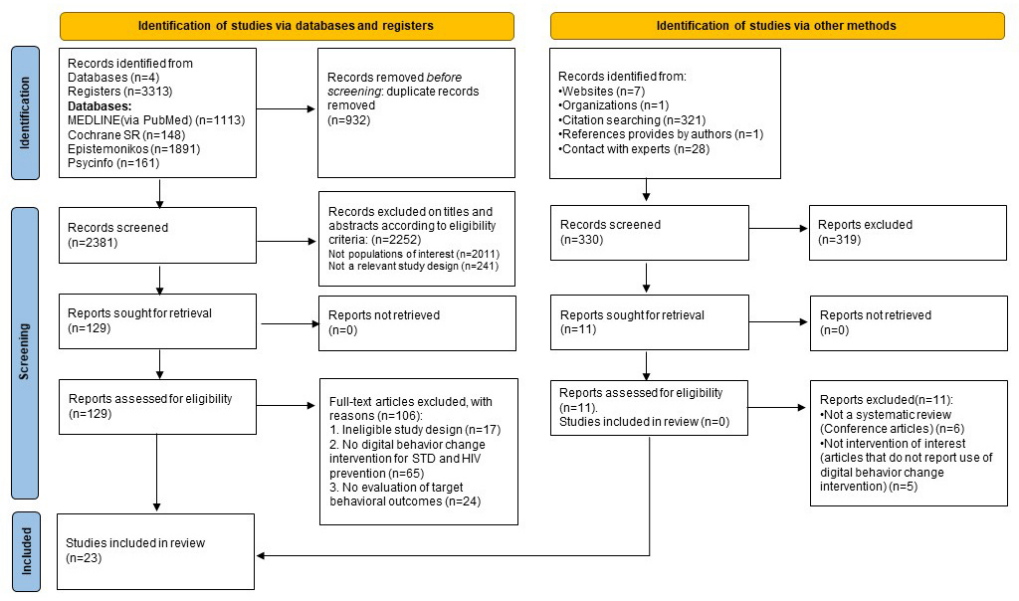
Study selection flow diagram for the overview of systematic reviews on digital behavior change interventions (DBCIs) for STI/HIV prevention, following the PRIOR reporting guideline. PRIOR: Preferred Reporting Items for Overviews of Reviews.

In this overview, a comprehensive search strategy was used, leveraging multiple key sources. Primary searches were conducted on the leading international databases MEDLINE (via PubMed), the Cochrane Database of SRs, Epistemonikos, and PsycINFO. Each database was searched independently using its native platform; no multidatabase platform (eg, EBSCOhost and Ovid) was used. To enhance the scope of our search, we conducted supplementary searches to identify any studies potentially overlooked by the primary search strategy or absent from the indexed databases. These supplementary efforts included (1) meticulous manual reviews of the references cited in the included studies, (2) examination of related SRs that shared at least one study with the reviews included in our study, and (3) additional records identified through various websites (n=7), organizations (n=1), citation searching (n=321), references provided by authors (n=1), and consultations with experts (n=28; Figure 1). Citation searching involved both backward citation screening (reference list checking) and forward citation tracking using Google Scholar. No additional search methods such as automated alerts, web-scraping tools, or application programming interface–based search retrieval were used.

Since this overview synthesizes published SRs rather than primary studies, study and trial registries (eg, ClinicalTrials.gov, International Clinical Trials Registry Platform) were not searched. Each source was rigorously scrutinized, with the date of the last search or consultation carefully documented to ensure the currency and relevance of the findings. Database-specific yields were MEDLINE (via PubMed; n=1113), Cochrane Library (n=148), PsycINFO (n=161), and Epistemonikos (n=1891), as detailed in Multimedia Appendix 2. Detailed methodologies and search strategies are available in Multimedia Appendix 2 (section 4).

### Search Strategy

The electronic search strategy (sections 5 and 6 in Multimedia Appendix 2) was developed and conducted under the supervision of an experienced librarian. The strategy did not undergo formal PRESS (Peer Review of Electronic Search Strategies) peer review. The search strategy was newly developed for this overview and was not adapted from any previous review. The first author performed the electronic search from the databases’ inception up to November 12, 2025, with no restrictions on publication date, language, or country of origin, following PRISMA-S recommendations to ensure transparent and reproducible reporting of search methods. No methodological search filters (eg, SR filters, randomized trial filters, and human-only filters) were applied beyond the predefined eligibility criteria. An earlier search completed on August 31, 2024, was rerun and updated in accordance with PRISMA-S guidance [38], to capture the most recent SRs before resubmission. Additionally, we manually searched the bibliographies of relevant reviews and the articles initially retrieved. Letters were also sent to authors and experts identified in the included and excluded studies during the screening stage to identify additional eligible studies. The eligibility criteria are listed in Table 1. Full search dates for each database, including the initial search (August 31, 2024) and rerun (November 12, 2025), are reported in Multimedia Appendix 2. Full search strategies for all databases, copied verbatim as executed, are provided in Multimedia Appendix 2.

### Study Selection Process

Following deduplication and a pilot test of the inclusion and exclusion criteria, 2 independent reviewers (GDA and DLA) screened all titles, abstracts, and full-text articles for eligibility without knowledge of each other’s decisions. For the deduplication process, all records retrieved from database searches and supplementary sources were imported into Collaboratron (Epistemonikos). The software’s automated similarity-detection algorithm was used to identify duplicate entries, followed by manual verification by 2 independent reviewers (GDA and DLA) to ensure accuracy. This hybrid deduplication approach, combining automated and manual procedures, aligns with PRISMA-S recommendations for transparent management of search records. Records from electronic and bibliographic searches were stored and full text screening was conducted using Collaboratron by Epistemonikos [44]. Differences between the 2 reviewers (GDA and DLA) were resolved through discussion, and a third reviewer (SSC) was consulted when necessary. The list of studies excluded after the full text review, along with reasons for exclusions, is detailed in section 7 in Multimedia Appendix 2.

We reached out to 28 authors (up to 3 email attempts) to request additional information, particularly on gray literature such as conference presentations and reports (see the list of experts in the Contacting Experts section of section 4 in Multimedia Appendix 2).

The interrater reliability was assessed using Cohen kappa coefficient [45]. Two independent reviewers (GDA and DLA) evaluated the full-texts (section 8 in Multimedia Appendix 2).

### Data Collection Process

We developed a data extraction tool in Microsoft Excel to obtain various study data recommended by Cochrane [46,47]. The data extracted from the SRs selected for the study was tested and calibrated by the team (section 9 in Multimedia Appendix 2). For this purpose, 1 author (GDA) created the spreadsheet and then extracted the data from 1 SR. Subsequently, 2 authors (GDA and DLA) independently extracted data from 3 SRs, and all authors provided feedback on whether the data elements were complete, and the extracted data were unambiguous. Once a consensus was reached through discussion, 1 author (GDA) created a data extraction manual for the spreadsheet, which can be found in Multimedia Appendix 2 (section 10).

### Data Items

Data items included SR characteristics, PICOS criteria, and variables related to DBCI (Textbox 1).

**Data items in this overview of systematic reviews.**

**Data items**

- Bibliographic information (author, year of publication, title, and aim of the SR).

**Population characteristics:**

- Participants (total number of participants included in the studies)
- Population age
- Specific population (men who have sex with men, lesbian, gay, bisexual, transgender, queer, and other sexual orientations and gender identities (LGBTQI+) individuals, people with a diagnosis of sexually transmitted disease [STD] without HIV)

**Study characteristics**

- Total number of studies included in the review
- Number of randomized controlled trials (RCTs) included
- Type of studies (only RCTs, only non-RCTs, including experimental nonrandomized and observational studies-, both RCTs and non-RCTs)
- Review period (range of publication years of the primary studies included in each systematic review, reflecting the temporal coverage of the synthesized evidence)
- Period or specific date range of the literature search (years during which databases were searched by the authors of each SR);
- Country or geographic location of the studies

**Intervention details**

- Target population
- Target behavior
- Explicit mention or extraction of theoretical frameworks used.
- Behavioral outcomes (condom use, frequency of unprotected sexual intercourse, number of sexual partners, STD and HIV testing, uptake of medical male circumcision, HIV counseling, vaccination)
- Cognitive outcomes (self-efficacy, STD and HIV-related knowledge, attitudes toward condom use, and pre-exposure prophylaxis awareness)
- Biological outcomes (acquisition of HIV or sexually transmitted infection)

**Intervention acceptability and feasibility**

- Acceptability: participants’ acceptance of the intervention
- Practicability: ease of implementation in the real world
- Effectiveness: achievement of the intervention objectives
- Affordability: cost-effectiveness of the intervention
- Spill-over effects: unintended consequences
- Equity: impact on health equity

**Technology and delivery methods**

- Mobile devices, desktop computers, digital billboards, wearable accessories, digital objects, and projection and holograms
- Mode of delivery of digital contents (audio calls and messages, video calls and messages, text and instant messages, emails, audio broadcasts and podcasts, websites and computer programs and apps, eBooks, virtual or augmented reality, artificial intelligence; for example, use of artificial intelligence–based chatbots to promote safe sex or other sexual behaviors)

**Other characteristics**

- Number of primary studies included and overlap, tools used to assess the risk of bias in primary studies, whether a meta-analysis was conducted, and certainty of evidence (Grading of Recommendations, Assessment, Development, and Evaluation)

### Quality Appraisal of the SRs

We performed critical appraisals of SRs using AMSTAR-2 [48], as outlined in our published protocol [39]. AMSTAR-2 consists of 16 items that assess the thoroughness of various aspects of a SR, such as the preparation process, literature search, study selection, data extraction, and analysis, as well as potential biases (eg, risk of bias, publication bias, or funding sources). Based on the type and number of weaknesses identified (ie, unmet items), the reviews were assigned a confidence rating: high, moderate, low, or critically low. Two authors (GDA and DLA) independently assessed all SRs using a spreadsheet (Microsoft Excel 2010) and reached consensus through discussion (section 11 in Multimedia Appendix 2).

### Overlap in Primary Studies Included in Reviews

To ensure the accuracy of the primary study outcome data and avoid overlap, we checked whether the included SRs shared overlapping primary studies. This was done by creating a citation matrix and calculating the overall corrected covered area (CCA) using Graphical Representation of Overlap for Overviews (GROOVE) [49]. The CCA quantifies the degree of overlap between primary studies included across SRs, calculated as the number of repeated primary studies (numerator) divided by the product of the total number of unique primary studies and the number of reviews, minus the total number of unique studies (denominator). The resulting value represents the proportion of shared evidence between reviews. A CCA of 0% to 5% indicates a slight overlap, 6% to 10% a moderate overlap, 11% to 15% a high overlap, and greater than 15% a very high overlap.”

### Data Synthesis Methods

#### Overview

Across the included SRs, the types of control or comparison groups varied substantially. In most cases, DBCIs were compared against nondigital or usual-care conditions, such as standard health education, printed materials, or no intervention controls. A smaller number of reviews included comparators that were themselves digital but lacked explicit behavior change components (eg, informational websites or SMS reminders without BCTs). This heterogeneity makes it difficult to disentangle whether observed effects are attributable to the digital delivery mode, the behavioral content, or both. Therefore, comparator conditions reported by each review were documented, and findings were interpreted with caution, emphasizing the combined influence of digital and behavioral mechanisms [50].

#### Outcome Definitions

Behavioral outcomes were classified according to the definitions provided in the included SRs. “STI and HIV prevention behaviors” encompassed all behavioral actions aimed at reducing infection risk, including but not limited to condom use, STI and HIV testing, vaccination, and adherence to treatment. “Safe sex behaviors” referred specifically to sexual practices such as consistent condom use, partner reduction, and negotiation of safer sex. This hierarchical approach was adopted to maintain consistency with the terminology used in the original reviews while avoiding redundancy between overlapping categories. A clarifying note was also added to Table 2 indicating that “safe sex behaviors” represent a subset of “STI and HIV prevention behaviors.

**Table 2:** Main characteristics of systematic reviews published between 2014 and 2015. “Safe sex behaviors” are a subset of “sexually transmitted infection and HIV prevention behaviors. The table summarizes study design, population, intervention features, behavioral and cognitive outcomes, and country income level based on World Bank classification.

Characteristics (N=23^a^)				Results
**SR^b^ design**				
	Number of studies included		514^c^	
	Participants (total)		129,481	
	Range of years of the studies included		(2014-2025)	
	**Place (country: geographic location)**			
		High-income countries	204	
		Upper-middle-income countries	22	
		Lower-middle-income countries	17	
	Only RCT^d^ (n=23)		9	
	Only non-RCT (includes nonrandomized experimental and observational studies) (n=23)		2	
	RCT and non-RCT (n=23)		12	
	Total RCTs (N=514)		410	
**Population type (n=23)**				
	Adolescents (population age 10 to 19 years)		14	
	Youth (20 to 29 years)		17	
	Adults ( >29 years)		15	
	Men who have sex with men		14	
	LGBTQI+^e^ individuals		10	
	People diagnosed with an STD^f^ ( without HIV)		10	
**Intervention characteristics (n=23)**				
	Target of behavior (prevention of STI^g^ and HIV)		19	
	Target behavior (safe sex)		16	
	Target behavior (STI and HIV testing)		17	
	Target behavior (treatment-related, ie, attend the appointment or getting treatment for an STI)		10	
	Environment: schools		10	
	Environment: universities (where it was implemented)		4	
	Environment: health centers (where it was implemented)		10	
	Explicit framework of theoretical domains of behavior change from the studies included in the SR		10	
	Description of behavior change techniques according to BCTTv1		2	
	Description according to Theoretical Domains Framework (TDF)		1	
**Behavioral outcomes (n=23)**				
	Condom use (internal or external)		19	
	Frequency in unprotected sexual intercourse		11	
	Number of sexual partners		7	
	STD and HIV testing		18	
	Uptake of medical male circumcision		4	
	HIV counseling		8	
	Get vaccinated against (VPH- HEP A y B)		7	
**Cognitive outcomes (mediators of prevention) (n=23)**				
	Self-efficacy		10	
	STD- and HIV-related knowledge		10	
	Attitudes toward condom use		8	
	Pre-exposure prophylaxis awareness		4	
**Biological outcomes**				
	HIV or STI acquisition (n=23)		8	
**Technology delivered (n=23)**				
	Mobile device		19	
	Desktop computer		12	
	Digital billboard		0	
	Wearable (clothing and accessory)		0	
	Digital object		0	
	Projection and hologram		0	
**Digital content type (n=23)**				
	Audio call and message		10	
	Video call and message		14	
	Text and instant message		18	
	Email		9	
	Video game		6	
	Audio broadcast and podcast		1	
	Website, computer, program and app		18	
	eBook		1	
	Virtual or augmented reality		2	
**Other descriptions**				
	Artificial intelligence-based chatbots for promoting safe sex or other sexual behaviors		0	
**Meta-analysis and certainty of evidence (n=23)**				
	Meta-analysis		7	
	Certainty of evidence (GRADE)^h^		4	
**APEASE^i^ (n=23)**				
	Acceptability		6	
	Practicability		0	
	Effectiveness		19	
	Affordability		4	
	Spill-over effects		3	
	Equity		2	

^a^Total number of SRs included in this overview.

^b^SR: systematic review.

^c^Total number of primary studies included in the SRs of this overview.

^d^RCT: randomized controlled trial.

^e^LGBTQI+: lesbian, gay, bisexual, transgender, queer, intersex, and plus.

^f^STD: sextually transmitted disease.

^g^STI: sexually transmitted infection.

^h^GRADE: Grading of Recommendations, Assessment, Development, and Evaluation.

^i^APEASE: Acceptability, Practicability, Effectiveness, Affordability, Spill-over effects, and Equity.

A written description was generated on the results of each SR on digital interventions for the prevention of STIs and HIV, and a table detailed the characteristics and outcomes of each review. The extracted data were synthesized using a predefined and team-approved template, identifying common themes and mapping them according to the stated objectives (section 9 in Multimedia Appendix 2).

Subgroup analyses were done to evaluate various factors, including the aim of the SR, the target population, behavioral outcomes, cognitive outcomes, biological outcomes, and the type of digital content and intervention. The mode of delivery (MoD) framework by Marques et al [51] was used to categorize interventions into types such as text and instant messages, video calls and messages, websites, computer programs and apps, emails, video games, audio broadcasts and podcasts, e-books, and virtual or augmented reality.

The effectiveness of digital interventions for STI and HIV prevention was assessed based on SRs that explicitly described the use of BCTs or the TDF. Subsequently, the impact of BCTs on each specific outcome was analyzed following the approach of Michie et al [52,53], allowing for the identification of the most effective techniques. Findings were synthesized into structured tables to visualize the impact of digital interventions across behavioral, cognitive, and biological domains.

Finally, a 5-level classification system was applied: “▼” indicated strong evidence of a negative effect; “O,” mixed or null evidence; “O−” and “O+,” a negative or positive effect with limited evidence; and “▲,” strong evidence of a positive effect. The strength of evidence was determined according to three criteria: (1) the number of SRs reporting consistent results in the same direction, (2) the methodological quality of these reviews based on AMSTAR-2, and (3) the presence of meta-analytic data when available. Strong evidence (“▲” or “▼”) required consistent findings across at least 2 high-quality reviews or meta-analyses, while limited evidence (“O−” or “O+”) was assigned when findings were reported in only one review or when quality or consistency was lower. Mixed or null evidence (“O”) indicated conflicting or inconclusive findings. This approach aligns with recent overviews applying the same evidence-grading framework for BCTs [53,54].

This system aims to provide clear guidance on which BCTs are most effective in digital interventions for STI and HIV prevention. The BCTs were coded according to the BCT Taxonomy version 1 (BCTTv1) [31,55], in studies that reported interventions using TDF, while the original descriptions were retained for studies that explicitly reported BCTs. This methodological approach ensured that conclusions were systematic, evidence-based, and aligned with established behavior change frameworks.

The SAGER [37] guidelines were used to ensure the consideration of sex and gender variables during the data extraction. These guidelines aim to prevent bias and improve the relevance and validity of findings by promoting the clear distinction between “sex” (biological differences) and “gender” (social and cultural factors), and its purpose is for these distinctions to be accurately reflected in study design, data analysis, and results reporting (sections 2 and 12 in Multimedia Appendix 2).

## Results

### Overview

From the 3643 records identified (3313 from electronic databases and 330 from bibliographic sources), 129 full-text articles were assessed for eligibility. Of these, 106 were excluded, leading to the inclusion of 23 SRs in this overview [56-78] (Figure 1). The interrater reliability for full-text screening of the initial 122 studies was robust (κ=0.88). An updated search conducted on November 12 yielded 378 additional records, of which 7 were assessed in full-text and 4 met the inclusion criteria. Additional studies were identified through supplementary methods, including website searches, citation searching, and expert consultations, ensuring a comprehensive review (section 4 in in Multimedia Appendix 2).

### Characteristics of SRs

The 23 SRs included in the overview were published between 2014 and 2025, and covered 514 primary studies, including 410 randomized controlled trials (RCTs). Of these reviews, 9 focused exclusively on RCTs [56-58,66,69,70,75,77,78], 2 on non-RCTs [72,73], and 12 included both study types [59-65,67,68,71,74,76]. These reviews encompassed 129,481 participants, with individual studies ranging from 2662 to 27,704 participants. A detailed appraisal of methodological quality and overlap across the included SRs is presented at the end of this section to contextualize confidence in the synthesized evidence. The targeted populations included adolescents (n=14), youth (n=17), adults (n=15), men who have sex with men (n=14), LGBTQ+ individuals (n=10), and people diagnosed with sexually transmitted diseases other than HIV (n=10; sections 13, 14a, and 14b in Multimedia Appendix 2).

Most studies were conducted in high-income countries, including the United States, Portugal, and Chile, followed by upper-middle-income countries such as China and South Africa, and lower-middle–income countries such as India and Kenya. Country income levels were classified according to the World Bank Country and Lending Groups (FY2025, Atlas method, USD), which use Gross National Income per capita as the defining criterion. Classifications were verified as of November 10, 2025, based on the latest publicly available dataset [79]. All data are reported, and detailed characteristics of the included SRs are presented in section 13 in in Multimedia Appendix 2. The synthesis of study characteristics is summarized in Table 2.

### SAGER Application

The SAGER guidelines revealed that most studies did not include sex- or gender-disaggregated data, and significant gender differences were generally not reported. Only one study, Kamitani et al [63], mentioned transgender participants, but without a detailed analysis (section 12 in in Multimedia Appendix 2).

### Characteristics of the Interventions

#### Overview

Across the included SRs, digital interventions targeted multiple prevention-related behaviors, including STI and HIV prevention, safer sex practices, and engagement with testing and sexual health services. Most SRs focused on STI and HIV prevention behaviors (19/23, 82.6%) safe-sex promotion (16/23, 69.6%), and STI and HIV testing (17/23, 73.9%). Additionally, 10 SRs (43.5%) incorporated strategies to enhance treatment adherence, such as attending medical appointments or completing syphilis treatment (Table 2 and section 15a in in Multimedia Appendix 2).

The research study settings varied, with health centers being the most common (10/23, 43.5%), followed by schools (10/23, 43.5%), while universities (4/23, 17.4%) were the least frequent. Regarding the use of theoretical frameworks, 10 SRs (43.5%) applied a behavior change framework, yet only 2 SRs (8.7%) explicitly described techniques based on the BCTTv1, and just one (4.3%) used the TDF (section 15a in in Multimedia Appendix 2).

In terms of behavioral outcomes, out of 23 SRs analyzed, condom use was assessed in 19 reviews (82.6%), while STI and HIV testing was reported in 18 reviews (78.3%). Other relevant outcomes included the frequency of unprotected sexual intercourse (47.8%), the number of sexual partners (30.4%), and vaccination against HPV or hepatitis (30.4%). Finally, the most analyzed cognitive outcomes were self-efficacy (43.5%), STI and HIV-related knowledge (43.5%), and attitudes toward condom use (34.8%), whereas PrEP awareness was examined in only 4 studies (17.4%; section 15b and 15c in in Multimedia Appendix 2).

#### MoD

According to the MoD classification by Marques et al [51], most interventions were delivered via mobile devices (n=19) and desktop computers (n=12), with text and instant messaging being the most common digital content type (n=18). Other content types included video calls and messages (n=14), emails (n=9), and video games (n=6). Notably, no studies used emerging delivery methods such as digital billboards, wearable accessories, digital objects, or projection and holograms (Table 2; section 15d in in Multimedia Appendix 2).

#### Theoretical Frameworks and BCTs

Of the 23 SRs analyzed, 10 (43.5%) incorporated behavioral theories, with the information-motivation-behavioral skills model (10 of 71 framework mentions, 14.3%), health belief model, and social cognitive theory being the most common. However, 13 reviews (56.5%) lacked any theoretical framework, reflecting inconsistent application of behavior change science. Only 4 (17.4%) reviews explicitly reported the identification or coding of BCTs or TDF [56,58,59,78] (section 15e in in Multimedia Appendix 2).

Overall, there was limited variability in the reporting of theoretical and behavioral frameworks across the 23 SRs. Explicit descriptions of BCTs or TDF mapping were rare, with most reviews indicating “not reported.” This pattern reflects heterogeneity in reporting practices across digital interventions for STI and HIV prevention and highlights that only a minority of reviews provided systematic or detailed descriptions of behavioral frameworks.

#### Effectiveness and Implementation of BCTs in Digital Interventions: Subset Analysis

This subsection focuses specifically on the subset of SRs (4 out of 23) that explicitly identified, coded, or analyzed BCTs within digital interventions for STI and HIV prevention. These reviews (Bailey et al [56]; Burns et al [58]; Clarke et al [59]; and Mo et al [78]) provided sufficient methodological detail to enable comparison of BCT use, frequency, and effectiveness. The remaining reviews, which did not report BCT coding or implementation frameworks, are synthesized in the previous sections that address broader behavioral, cognitive, and biological outcomes. This clarification ensures transparency and maintains consistency with the overview’s comprehensive scope.

DBCIs for STI and HIV prevention have incorporated various BCTs; however, their explicit classification using standardized frameworks such as the BCTTv1 or TDF remains limited. Among the studies analyzed, Burns et al [58] and Clarke et al [59] reported interventions explicitly coded using BCTTv1, whereas Bailey et al [56] used domains associated with TDF to describe behavioral determinants. Mo et al [78] expanded the evidence base by systematically identifying and mapping BCTs across digital HIV prevention interventions for adolescents and young people (Table 3).

**Table 3:** Summary of behavioral determinants, frequently reported behavioral change techniques (BCTs), and observed effectiveness of digital interventions for the prevention of sexually transmitted infections (STIs) and HIV based on 4 reviews (Bailey et al [56]; Burns et al [58]; Clarke et al [59]; and Mo et al [78]). Includes intervention design, target population, mode of delivery, and AMSTAR-2 quality rating. BCT codes correspond to Behavior Change Technique Taxonomy version 1.

Reference and review type	Review period	Mode of delivery	N Primary studies and study designs	Target population and number of participants	Target of behavior	Intervention effectiveness and observed behavior change	Determinants of behavior	Most frequently used BCTs and components	Statistical significance and effect size (*P* value, Cohen *d*, r)	AMSTAR 2 Rating
Bailey et al [56] SR^a^ and meta-analysis	Searches: from 2014 to June 2017 Publication of primary studies included: 1991-2017	Web-based programs, mobile apps, online modules	31 studies RCTs^b^	Population: young people, men who have sex with men (MSM), HIV-positive people, at-risk adults, African American women. Total: 11,293 participants - IDI vs. minimal intervention: 10,423 participants - IDI vs. face-to-face intervention: 870 participants	Prevention of HIV and other STIs, promotion of safe sex behaviors, adherence to testing and treatment. Condom use, partner reduction, and safe sex negotiation	Increased HIV-related knowledge (moderate effect) - small improvement in behavioral intention - Positive effect on HIV prevention behaviors - No clear impact on self-efficacy - No significant effect on biological outcomes (STI and HIV acquisition, viral load)	Goals, behavioral regulation, knowledge, emotion, optimism, beliefs about capabilities	Goal setting (1.1), commitment (1.9), feedback on behavior (2.2), biofeedback (2.6), information about antecedents (4.2), re-attribution (4.3), information about health consequences (5.1), salience of consequences (5.2), verbal persuasion (15.1), self-talk (15.4)	HIV-related knowledge: SMD^c^=0.56 (95% CI 0.33-0.80) - HIV prevention self-efficacy: SMD=0.13 (95% CI 0.00-0.27) - HIV prevention intention: SMD=0.16 (95% CI 0.06-0.26) - HIV prevention behaviors: OR^d^ 1.28 (95% CI 1.04-1.57) - Biological outcomes (STI and HIV acquisition, viral load): OR 1.48 (95% CI 0.96-2.28), *P*=.08 (not significant)	High No critical flaws One noncritical weakness (no information on funding sources)
Burns et al [58] SR of RCTs	Searches: January 1999 and July 2014 Publication of primary studies included:: 2006-2014	Mobile phone-based interventions, SMS reminders, mobile apps, video messages	10 studies RCTs	Population: General population, at-risk adults, young people, (MSM) Total: 16,773 participants	Promotion of sexual health services uptake, reduction of risky sexual behaviors, reduction of recall bias in self-reported sexual activity	Two trials showed significant increases in clinic attendance with SMS reminders. - One trial improved sexual health knowledge. - No trials showed significant increases in condom use. - One trial found mobile technology acceptable for sexual health data collection	Goals, intentions, behavioral regulation, knowledge, social influences, environmental context and resources, reinforcement	Goal Setting (1.1), feedback on Behavior (2.2), information about health consequences (5.1), demonstration of behavior (6.1), social comparison (6.2), prompts/cues (7.1), material incentives (10.1)	Clinic attendance: SMS reminders significantly increased attendance (RR^e^ 0.86, 95% CI 0.74-1.00) - Chlamydia retesting: SMS reminders increased retesting (RR 4.5, 95% CI 1.05-19.22) - HIV testing uptake: no significant effect (RR 0.94, 95% CI 0.81-1.09) - Sexual health knowledge: SMS improved knowledge (RR 1.75, 95% CI 1.11-2.77) - Condom use: no significant changes (RR 0.87, 95% CI 0.62-1.24)	Moderate No critical flaws More than one noncritical weakness (no information on funding sources, publication bias reported but not discussed)
Clarke et al [59] SR	Searches: 1 January 2000 to 1 September 2021. Publication of primary studies included:: 2011-2018	Digital interventions including SMS (text messages), social media, and app-based messaging	13 studies (RCTs=5 Non RCTs before-after=8)	Population: Adolescents, youth, adults, MSM, LGBTQI+^f^. Total: not reported	Increasing attendance at scheduled sexual health appointments	Behavioral interventions increased attendance at scheduled sexual health appointments. Text messages were the most frequently used MoD^g^. Some interventions were effective, while others had mixed results	Beliefs about consequences, environmental context and resources, emotion, reinforcement, social influence, optimism	Credible source (9.1), prompts/cues (7.1), social support (3.2), social reward (10.4), self-incentive (10.5), restructuring of the environment (12.1, 12.2), focus on past success (15.3), vicarious consequences (16.3)	Some interventions significantly increased attendance while others had mixed results	Critically Low More than one critical flaw (no justification for excluding individual studies, no consideration of bias when interpreting results) More than one noncritical weakness (study selection not done in duplicate, no information on funding sources)
Mo et al [78] SR	Searches: January 2008 to November 2024. Publication of primary studies included: 2008-2023	Mobile apps, SMS text messaging, web-based modules, computer-based digital game (IYG-Tech), online educational platforms	34 studies (RCTs, quasi-experimental, and observational designs)	Population: adolescents and young people (10-29 y). Total: Not reported	Prevention of HIV; promotion of safer sex practices; HIV testing; risk reduction behaviors	Narrative synthesis indicated consistent improvements in cognitive determinants (HIV-related knowledge, self-efficacy, perceived risk). Small, inconsistent effects were reported for condom use. Some interventions showed increases in HIV testing motivation or intentions, but behavioral outcomes were heterogeneously measured and rarely pooled	Knowledge, beliefs about consequences, behavioral skills, self-efficacy, environmental context and resources, motivation	Information about health consequences (5.1), feedback on behavior (2.2), prompts/cues (7.1), goal setting (1.1), problem solving (1.2), demonstration of behavior (6.1), social support (3.1), self-monitoring (2.3)	No pooled effect sizes reported. Several primary studies demonstrated significant improvements in HIV knowledge and self-efficacy but effects could not be meta-analyzed	Critically Low More than one critical flaw (no justification for excluding individual studies, no consideration of bias when interpreting results) More than one noncritical weakness (study selection not done in duplicate, no information on funding sources)

^a^SR: systematic review.

^b^RCT: randomized controlled trial.

^c^SMD: standardized mean difference.

^d^OR: odds ratio.

^e^RR: relative risk.

^f^LGBTQI+: lesbian, gay, bisexual, transgender, queer, and other sexual orientations and gender identities.

^g^MoD: mode of delivery.

In total, 26 BCTs were reported. The most frequently used in these interventions included goal setting (1.1), feedback on behavior (2.2), and the use of prompts and cues (7.1), commonly delivered through mobile apps, text messaging (SMS), online modules, and digital games. Other approaches included commitment strategies (1.9), biofeedback (2.6), social support (3.1, 3.2), and providing information about health consequences (5.1). These techniques targeted key behavioral determinants such as knowledge, behavioral regulation, social influence, and reinforcement strategies, aiming to enhance self-efficacy, motivation, and risk awareness (Table 3).

**Figure 2:**
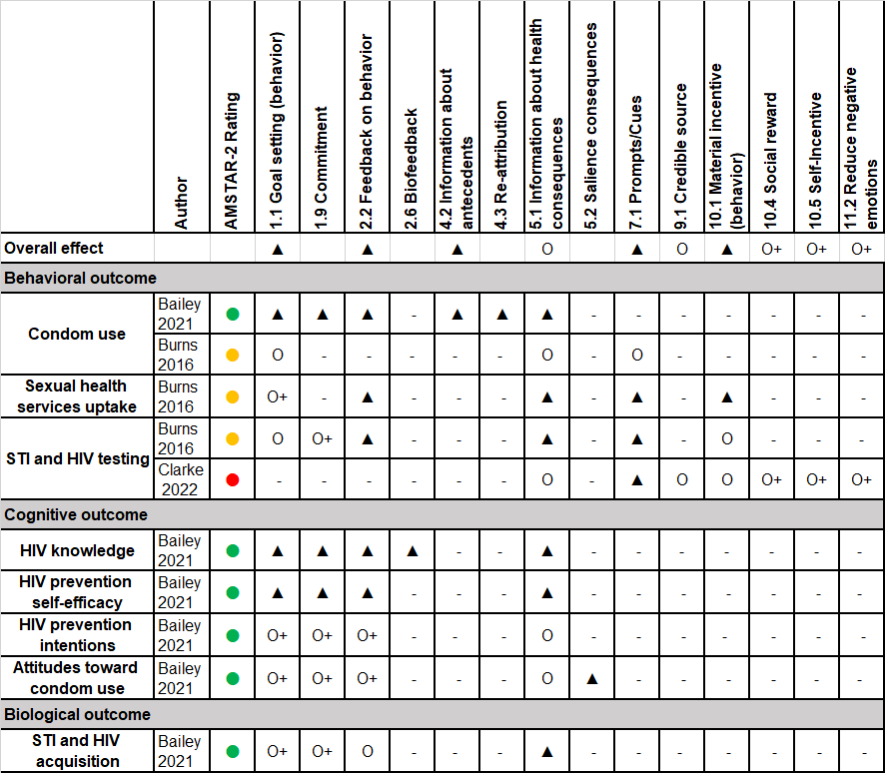
Summary of the effectiveness of behavior change techniques in digital interventions for sexually transmitted infection and HIV prevention.

Table 3 summarizes behavioral determinants, commonly used BCTs, and intervention effectiveness. Figure 2 synthesizes the impact of specific BCTs across behavioral, cognitive, and biological outcomes. Findings on BCT effectiveness were drawn from the 3 SRs that reported effect estimates (Bailey et al [56]; Burns et al [58]; Clarke et al [59]). Mo et al [78] contributed descriptive evidence on BCT implementation but did not report quantitative effect estimates. This section also highlights how BCTs were implemented across SMS-based strategies, mobile apps, online platforms, and digital learning modules.

#### Behavioral Outcomes

##### Condom Use

Digital interventions showed mixed effectiveness. Bailey et al [56] found a significant increase in condom use (odds ratio [OR] 1.28, 95% CI 1.04-1.57, ▲ evidence), supporting the role of goal setting (1.1), commitment (1.9), and feedback (2.2). Conversely, Burns et al [58], reported no significant effect (relative risk [RR] 0.87, 95% CI 0.62-1.24, O evidence), suggesting that effectiveness varied by population and intervention design.

Regarding mobile apps for risk awareness [56], digital interventions focusing on health consequences (5.1) significantly improved HIV-related knowledge (standardized mean difference [SMD]=0.56, 95% CI 0.33-0.80), reinforcing the role of interactive digital tools in promoting condom use behaviors.

##### Sexual Health Services Uptake

To ensure conceptual clarity, in this overview STI and HIV testing refers to diagnostic testing behaviors, whereas sexual health services uptake encompasses broader engagement with preventive or clinical services, including retesting when reported as part of general service use [58].

Mixed results (O+ to ▲) were found regarding the effectiveness of goal setting (1.1) and Feedback (2.2) in increasing engagement with sexual health services. SMS reminders significantly increased clinic attendance (RR 0.86, 95% CI 0.74-1) and chlamydia retesting rates (RR 4.5, 95% CI 1.05-19.22) [58]. SMS reminders providing health-related information (5.1) and material incentives (10.1) were effective in some cases, particularly for STI retesting, though results varied across populations.

Regarding SMS feedback and goal setting [58], participants received weekly SMS messages inquiring about risky sexual behaviors, followed by feedback and goal-setting prompts to encourage health service use.

##### STI/HIV Testing

Evidence from Burns et al [58] was mixed (O to ▲), with Feedback (2.2) and prompts and cues (7.1) being somewhat effective in encouraging STI testing. Clarke et al [59] found that personalized SMS reminders significantly improved STI retesting rates (56% vs. 33%; *P*<.01). However, material incentives (BCT 10.1) yielded mixed effects (O), as financial rewards increased short-term attendance but did not sustain engagement.

Regarding personalized SMS reminders and incentives [59], tailored SMS messages encouraged STI retesting, with financial incentives increasing attendance (29.17% in the incentive group vs. 0% in the control group).

#### Cognitive Outcomes

##### HIV-Related Knowledge

Digital interventions incorporating Information about Health Consequences (5.1) and Biofeedback (2.6) significantly improved HIV-related knowledge (SMD=0.56, 95% CI 0.33-0.80, ▲ evidence). These interventions relied on mobile apps to enhance risk awareness through educational modules and interactive tools [56].

##### HIV Prevention Self-Efficacy

Strong evidence (▲) supported Goal Setting (1.1), Commitment (1.9), and Feedback (2.2) in improving self-efficacy for HIV prevention. Self-monitoring and digital education modules were key to reinforcing behavior change [56].

##### HIV Prevention Intentions and Attitudes Toward Condom Use

For both outcomes, evidence was weaker (O+ to O), indicating that Goal Setting (1.1) and Feedback (2.2) may contribute to positive attitudes toward condom use, but their long-term impact remains uncertain (SMD=0.16, 95% CI 0.06-0.26). Health Consequences (5.1) showed inconsistent effects, suggesting that behavior change may require additional reinforcement. [56].

Regarding mobile apps for risk awareness [56]: digital interventions focusing on HIV prevention knowledge were designed to improve risk awareness and motivation, though long-term behavior adoption remained a challenge.

Complementary evidence was provided by Mo et al [78], who systematically reviewed digital HIV prevention interventions for adolescents and young adults and identified a broad set of BCTs mapped across the included programs. Although this review did not quantify behavioral or biological outcomes associated with specific BCTs, it reported consistent improvements in key cognitive determinants—particularly HIV-related knowledge, prevention self-efficacy, and attitudes toward condom use. These findings reinforce the role of information-based strategies (eg, providing information about health consequences, 5.1), feedback mechanisms (2.2), and prompts and cues (7.1) as foundational techniques supporting cognitive readiness for behavior change in digital sexual health interventions.

#### Biological Outcomes: STI/HIV Acquisition

Weak positive evidence (O+ to O) was assigned to goal setting (1.1), commitment (1.9), and feedback (2.2), with ▲ (strong evidence) for health consequences (5.1). Despite improvements in knowledge and behavioral determinants, these interventions did not significantly reduce STI and HIV acquisition rates (OR 1.48, 95% CI 0.96-2.28; *P*=.08) [56].

In Figure 2, summary of the effectiveness of individual BCTs reported in the 3 reviews included in the BCT subset analysis. Outcomes are grouped into behavioral (eg, condom use, STI and HIV testing), cognitive (eg, knowledge, self-efficacy), and biological domains. Effect strength was graded using a 5-level evidence classification system adapted from Michie et al. (2018) [9] and Mair et al. (2023) [53]: “▲” denotes strong positive. “O” denotes mixed or null, “O+/O−” denotes limited positive or negative, and “▼” denotes strong negative. AMSTAR-2 quality ratings are indicated by colored circles as follows: Green = High, Yellow = Moderate, Orange = Low, Red = Critical Low Classification of effectiveness from this systematic reviews and meta-analyses. ▲ = Positive effect of BCT based on good evidence such as subgroup or regression analyses. O+ = Positive effect of BCT based on low evidence such as frequency of individual BCTs within effective interventions. O = Mixed evidence or no effect. O− = Negative effect of BCT based on low level of evidence such as frequency of individual BCTs within effective interventions. ▼ = Negative effect of BCT based on good evidence such as subgroup or regression analyses.

### Quality Appraisal of the SRs

Confidence levels were determined according to the AMSTAR-2 criteria described in the Methods section, based on the number and severity of critical and noncritical weaknesses. Overall, confidence in the results was high in 21.7% (5/23) of the SRs, moderate in 13% (3/23), low in 17.4% (4/23), and critically low in 47.8% (11/23). The most common weaknesses identified were the absence of reported funding sources for primary studies (22/23, 95.7%), the lack of a full list of excluded studies (13/23, 56.5%), and the omission of a review protocol (10/23, 43.5%; section 16 in in Multimedia Appendix 2).

### Overlap in Primary Studies Cited in Reviews

The overlap assessment revealed a minimal overlap in the 321 primary studies, most of which were cited only once in the 23 SRs, with a CCA of 2.70. Comparisons between pairs of SRs indicated that 82.6% (209 out of 253) had a low overlap (<5%), 8.7% (22 out of 253) had a moderate overlap (5% to <10%), 4% (10 out of 253) had a high overlap (10% to <15%), and 4.7% (12 out of 253) had a very high overlap (≥15%).

Given that 4 reviews (Bailey et al [56]; Burns et al [58]; Clarke et al [59]; and Mo et al [78]) provided explicit coding or mapping of BCTs, these were analyzed as a separate methodological subset (Table 3). The overlap assessment indicated consistently slight overlap (<5%) among them, confirming that this BCT-focused evidence base draws on distinct sets of primary studies. These findings are illustrated in sections 17 and 18 in in Multimedia Appendix 2.

## Discussion

### Principal Results

This overview synthesized evidence from 23 SRs to evaluate the behavioral determinants, theoretical foundations, and effectiveness of DBCIs for preventing STIs and HIV. Consistent with the study aims, the findings indicate that DBCIs, particularly SMS reminders, mobile apps, and interactive web-based programs, can enhance engagement with sexual health services and increase STI and HIV testing. Cognitive determinants, such as HIV-related knowledge, motivation, and self-efficacy, also improved across numerous interventions. The most frequently identified and effective BCTs included goal setting, feedback on behavior, and prompts and cues. However, the high heterogeneity of intervention formats and the inconsistent reporting of BCTs across reviews limit the ability to draw definitive conclusions regarding the independent contribution of specific components. Taken together, these findings offer a clear synthesis of behavioral mechanisms across digital interventions and strengthen confidence in the patterns observed across reviews.

Only 4 reviews, Bailey et al [56], Burns et al [58], Clarke et al [59], and Mo et al [78], explicitly coded or mapped BCTs using standardized frameworks (BCTTv1 or TDF), allowing for a focused subset analysis. For the remaining reviews, behavioral mechanisms could only be inferred from narrative descriptions. The GROOVE overlap analysis confirmed minimal overlap across the 321 primary studies, indicating that the synthesized evidence draws from distinct and independent datasets. This methodological combination strengthens the validity of the synthesized findings and responds directly to recent calls for more mechanism-oriented evidence synthesis in digital health [80].

### Interpretation of Findings

Overall, DBCIs show considerable promise in supporting STI and HIV prevention, particularly through timely reminders, personalized feedback, and interactive educational content. SMS-based interventions consistently improved clinic attendance and STI retesting, reinforcing the well-established role of prompts and cues in facilitating preventive behaviors. Web-based programs and mobile apps contributed to enhanced knowledge, motivation, and behavioral skills, aligning with established behavioral models emphasizing the importance of cognitive determinants. This overview highlights that cognitive determinants represent the most consistent pathways of change in DBCIs, clarifying how digital components influence prevention behaviors.

Behavioral outcomes such as condom use demonstrated mixed effects: Bailey et al [56] reported significant improvements, whereas Burns et al [58] found no notable impact. These differences likely reflect variations in populations, intervention content, MoDs, and follow-up durations, as well as the extent to which interventions incorporated explicit BCTs or theoretical frameworks. Findings from Mo et al [78] further highlight the inconsistency in how interventions implement and report behavior change strategies, particularly among adolescent populations. These discrepancies underscore that digital modalities may be more effective when they target motivational and self-regulatory determinants rather than complex interpersonal behaviors, a distinction that has been underexplored in prior reviews.

The limited and inconsistent application of behavioral theory across reviews is a central issue. Nearly half of the reviews did not reference any behavioral framework, despite strong evidence from broader health literature showing that theoretically grounded digital interventions are more effective and more interpretable. This gap constrains the field’s ability to identify mechanisms of action and optimize intervention design. By systematically examining these gaps, our overview provides conceptual clarity on how limited theoretical integration constrains interpretability, scalability, and optimization of digital interventions. Addressing these gaps is essential for moving the field toward theory-driven digital prevention, where mechanisms of action are explicitly linked to intervention components and outcomes.

The updated search added 4 recent SRs [75-78], which expand the evidence base with up-to-date findings on digital PrEP adherence support, smartphone-based HIV prevention tools, and BCT-coded adolescent interventions. However, their conclusions mirror earlier patterns: digital tools consistently improve testing and cognitive outcomes, whereas sustained behavioral and biological impacts remain inconsistent. By incorporating the most recent evidence available, including 4 SRs published in 2024-2025, this overview offers a timely and comprehensive picture of current digital prevention strategies and how they engage (or fail to engage) key behavioral mechanisms.

### Comparison With Prior Work

Our findings align with prior work demonstrating that digital interventions can effectively promote preventive health behaviors when grounded in behavioral theory and equipped with active engagement strategies. Studies by Simoni et al [23], Albarracín et al [34], and Thomas Craig et al [24] underscore the importance of behavioral mechanisms, self-regulation processes, and contextual tailoring, elements only partially reflected across the reviews included in this overview. Unlike previous syntheses, this overview integrates behavioral mechanisms, intervention content, and methodological quality to provide a more coherent understanding of how and why DBCIs work. This integration allows for a more granular understanding of intervention pathways than outcome-only syntheses can provide.

A key divergence from other health domains (eg, mental health and chronic disease management) is the limited integration of emerging digital technologies, such as AI-driven personalization, conversational agents, and virtual or augmented reality, in STI and HIV prevention. Similarly, sex- and gender-disaggregated analyses remain rare, with only 1 review explicitly addressing transgender populations. This raises concerns about equity and generalizability in digital sexual health research. This highlights a broader limitation in the STI and HIV prevention literature: the field has been slower than other digital health domains to adopt advanced technologies and rigorous behavioral frameworks, reducing its capacity to generate equitable and generalizable impact. Future digital prevention efforts must bridge this technological and conceptual gap to ensure that innovation translates into equitable public health impact. Extending beyond earlier reviews, this overview examines not only whether digital interventions work but also how and why they work, through explicit mapping of behavioral mechanisms across reviews.

### Strengths and Limitations

This overview offers the most comprehensive behavioral synthesis to date of digital interventions for STI and HIV prevention, following the PRISMA-S (Multimedia Appendix 3). The use of AMSTAR-2 enabled robust appraisal of methodological quality, while GROOVE allowed quantification of overlap across primary studies. Together, the minimal overlap and explicit behavioral coding enhance confidence in the synthesized evidence.

However, several limitations should be acknowledged. First, conclusions depend on the quality and reporting of the included SRs, nearly half of which were rated critically low by AMSTAR-2. Second, BCT coding was absent or insufficient in most reviews, restricting the depth of mechanistic synthesis. Third, high intervention heterogeneity precluded meta-analytic pooling and limits comparability. Finally, reliance on SRs means that relevant primary studies not captured in those reviews may have been missed.

Despite these limitations, the overview provides a clear, methodologically grounded synthesis of how digital tools contribute to STI and HIV prevention and where future research should focus. This multilayered approach responds to recent calls for more rigorous, mechanism-oriented evidence synthesis capable of informing real-world decision-making and aligns with emerging frameworks such as evidence-based X, which emphasize the integration of mechanisms, context, and methodological rigor in digital health research [80].

### Conclusions

DBCIs represent a promising and scalable strategy for strengthening STI and HIV prevention, particularly for improving testing behaviors and key cognitive determinants. Interventions incorporating goal setting, feedback on behavior, and prompts and cues show the most consistent positive effects, whereas outcomes related to condom use and biological measures remain mixed. The inconsistent application and reporting of behavioral theory across reviews limits both interpretability and scalability.

Building on prior work, this overview provides a novel contribution by integrating effectiveness evidence with a systematic mapping of BCTs and theoretical mechanisms, a perspective that has been largely absent from earlier syntheses. By identifying which active components are most consistently associated with beneficial outcomes and highlighting persistent reporting and methodological gaps, this study offers actionable guidance for designing next-generation digital interventions. These findings, which incorporate evidence updated through 2025, have pragmatic real-world implications for researchers, clinicians, and policymakers seeking to develop scalable, culturally responsive, and equity-focused digital prevention programs that meaningfully address the behavioral pathways that drive STI and HIV risk across diverse populations. Collectively, these insights offer a pathway for accelerating the development of digital public health tools that are both evidence-based and behaviorally informed.

### Funding

This work was funded by the ANID, the Chilean government, the National Scientific and Technological Development Fund (FONDECYT) research project number 1250316. The funders had no role in the study design; data collection, analysis, or interpretation; the drafting of the report; or the decision to submit the paper for publication.

### Data Availability

Data sharing is not applicable to this article as no new datasets were generated or analyzed during this study. All data used in this overview were extracted from previously published systematic reviews and are included in the manuscript and its supplementary materials.

## Appendix

Multimedia Appendix 1 Reporting guideline for overviews of reviews of healthcare interventions: development of the PRIOR statement.

Multimedia Appendix 2 Behavioral Determinants and Effectiveness of Digital Behavior Change Interventions for STI/HIV Prevention: An Overview of Systematic Reviews.

Multimedia Appendix 3 PRISMA-S Checklist for Reporting Literature Searches.

## Abbreviations

BCT: behavior change technique
BCTTv1: behavior change technique taxonomy version 1
CCA: corrected covered area
DBCI: digital behavior change intervention
GROOVE: Graphical Representation of Overlap for Overviews
HPV: human papillomavirus
MoD: mode of delivery
OR: odds ratio
PICOS: Population, Intervention, Comparison, Outcome, Study type
PrEP: pre-exposure prophylaxis
PRESS: Peer Review of Electronic Search Strategies
PRIOR: Preferred Reporting Items for Overviews of Reviews
PRISMA-S: Preferred Reporting Items for SRs and Meta-Analyses Literature Search Extension
RCT: randomized controlled trial
RR: relative risk
SAGER: Sex and Gender Equity in Research
SMD: standardized mean difference
SR: systematic review
STD: sexually transmitted disease
STI: sexually transmitted infection
TDF: theoretical domains framework
