# Supplementary material for: Behavioral Determinants and Effectiveness of Digital Behavior Change Interventions for the Prevention of Sexually Transmitted Infections and HIV: Overview of Systematic Reviews
Source: J Med Internet Res. 2026 Jan 29;28:e74201. doi: 10.2196/74201 (PMC12902757; doi:10.2196/74201)
Supplement: Multimedia Appendix 1 [file jmir_v28i1e74201_app1.docx]

**Appendix 1**

**PRIOR Checklist**

**Reporting guideline for overviews of reviews of healthcare interventions: development of the PRIOR statement.**

| **Section**  Topic | **#** | **Item** | **Location reported on page #** |
| --- | --- | --- | --- |
| **TITLE** | | |  |
| Title | 1 | Identify the report as an overview of reviews. | 1 |
| **ABSTRACT** | | |  |
| Abstract | 2 | Provide a comprehensive and accurate summary of the purpose, methods, and results of the overview of reviews. | 2 |
| **INTRODUCTION** | | |  |
| Rationale | 3 | Describe the rationale for conducting the overview of reviews in the context of existing knowledge. | 2-3 |
| Objectives | 4 | Provide an explicit statement of the objective(s) or question(s) addressed by the overview of reviews. | 3 |
| **METHODS** | | |  |
| Eligibility criteria | 5a | Specify the inclusion and exclusion criteria for the overview of reviews. If supplemental primary studies were included, this should be stated, with a rationale. | 4, Table 1 |
|  | 5b | Specify the definition of ‘systematic review’ as used in the inclusion criteria for the overview of reviews. | 5 (note) |
| Information sources | 6 | Specify all databases, registers, websites, organizations, reference lists, and other sources searched or consulted to identify systematic reviews and supplemental primary studies (if included).  Specify the date when each source was last searched or consulted. | 5-6, Appendix 3,4 y 5 |
| Search strategy | 7 | Present the full search strategies for all databases, registers and websites, such that they could be reproduced. Describe any search filters and limits applied. | 5-6, Appendix 4-5 |
| Selection process | 8a | Describe the methods used to decide whether a systematic review or supplemental primary study (if included) met the inclusion criteria of the overview of reviews. | 6 |
|  | 8b | Describe how overlap in the populations, interventions, comparators, and/or outcomes of systematic reviews was identified and managed during study selection. | 8 |
| Data collection process | 9a | Describe the methods used to collect data from reports. | 6, Appendix 8 |
|  | 9b | If applicable, describe the methods used to identify and manage primary study overlap at the level  of the comparison and outcome during data collection. For each outcome, specify the method used to illustrate and/or quantify the degree of primary study overlap across systematic reviews. | 6-7, 15, Figure 2 and Appendix 16 |
|  | 9c | If applicable, specify the methods used to manage discrepant data across systematic reviews during data collection. | 6, Appendix 9 |
| Data items | 10 | List and define all variables and outcomes for which data were sought. Describe any assumptions made and/or measures taken to identify and clarify missing or unclear information. | 6-7, Textbox1 Appendix 8-9 |
| Risk of bias assessment | 11a | Describe the methods used to *assess* risk of bias or methodological quality of the included systematic reviews. | 8, Appendix 10 |
|  | 11b | Describe the methods used to *collect* data on (from the systematic reviews) and/or *assess* the risk of bias of the primary studies included in the systematic reviews. Provide a justification for instances where flawed, incomplete, or missing assessments are identified but not re-assessed. | 7 |
|  | 11c | Describe the methods used to *assess* the risk of bias of supplemental primary studies (if included). | NA |
| Synthesis methods | 12a | Describe the methods used to summarize or synthesize results and provide a rationale for the choice(s). | 8 |
|  | 12b | Describe any methods used to explore possible causes of heterogeneity among results. | 8 |
|  | 12c | Describe any sensitivity analyses conducted to assess the robustness of the synthesized results. | 8 |
| Reporting bias assessment | 13 | Describe the methods used to *collect* data on (from the systematic reviews) and/or *assess* the risk of bias due to missing results in a summary or synthesis (arising from reporting biases at the levels of the systematic reviews, primary studies, and supplemental primary studies, if included). | 14, Table 5, Appendix 10 |
| Certainty assessment | 14 | Describe the methods used to *collect* data on (from the systematic reviews) and/or *assess* certainty (or confidence) in the body of evidence for an outcome. | NA |
| **RESULTS** | | |  |
| Systematic review and supplemental primary study selection | 15a | Describe the results of the search and selection process, including the number of records screened, assessed for eligibility, and included in the overview of reviews, ideally with a flow diagram. | 9-20, Figure 1, |
|  | 15b | Provide a list of studies that might appear to meet the inclusion criteria, but were excluded, with the main reason for exclusion. | Appendix 7 |

From: (Gates M, Gates A, Pieper D, et al. Reporting guideline for overviews of reviews of healthcare interventions: development of the PRIOR statement. BMJ 2022;378:e070849. doi:10.1136/bmj-2022-070849.)
