# Supplementary material for: Behavioral Determinants and Effectiveness of Digital Behavior Change Interventions for the Prevention of Sexually Transmitted Infections and HIV: Overview of Systematic Reviews
Source: J Med Internet Res. 2026 Jan 29;28:e74201. doi: 10.2196/74201 (PMC12902757; doi:10.2196/74201)
Supplement: Multimedia Appendix 2 [file jmir_v28i1e74201_app2.docx]

**Appendix 2**

Contents

[Section 2 – SAGER Guidelines 2](#_Toc218584102)

[Section 3. Registered in PROSPERO (CRD42023485887) 3](#_Toc218584103)

[Section 4 - Identification of studies via other methods (Grey Literature Sources) 8](#_Toc218584104)

[Websites 8](#_Toc218584105)

[Organizations 8](#_Toc218584106)

[References Provided by Authors 8](#_Toc218584107)

[Contacting Experts 9](#_Toc218584108)

[Citation Searching 10](#_Toc218584109)

[Section 5 – Search strategy in line-by-line format 22](#_Toc218584110)

[Section 6 – Strategy in block format 26](#_Toc218584111)

[Section 7 – List of excluded studies 27](#_Toc218584112)

[Section 8 – Intercoder reliability calculation report 37](#_Toc218584113)

[Section 9– Data extraction form 38](#_Toc218584114)

[Section 10 – Coding Manual for Study 1. Project Title: Digital Behavior Change Interventions to Prevent Sexually Transmitted Infections (STIs) Including HIV: Evidence Reviews and Integrated Report on the Quantitative and Qualitative Evidence. 40](#_Toc218584115)

[Section 11 – Risk of Bias Assessment (Critical Appraisal of Systematic Reviews) 47](#_Toc218584116)

[Section 12 – Assessment of Sex and Gender Considerations in Included Studies 51](#_Toc218584117)

[Section 13 – Characteristics of Systematic Reviews 53](#_Toc218584118)

[Section 14 – Participant Characteristics 57](#_Toc218584119)

[Section 14 a. Participant characteristics by age and target population 57](#_Toc218584120)

[Section 14 b. Grouping by Target Population and authors of systematic reviews 59](#_Toc218584121)

[Section 15 – Characteristics of Digital Interventions 60](#_Toc218584122)

[Section 15a – Characteristics of Digital Interventions about target population, target behavior, and where were implemented. 60](#_Toc218584123)

[Section 15b – Characteristics of Digital Interventions about description about theory used and behavioral outcomes 61](#_Toc218584124)

[Section 15c – Characteristics of Digital Interventions about cognitive outcomes, biological outcomes and, criteria APEASE 62](#_Toc218584125)

[Section 15d – Characteristics of Digital Interventions about Mode of Delivery (MoD) 64](#_Toc218584126)

[Section 15e – Characteristics of Digital Interventions about distribution of theoretical frameworks used in Interventions use of theory, Behavioral Determinants and Behavior change techniques (BCTs) 67](#_Toc218584127)

[Section 16 – Critical assessments based on AMSTAR 2 70](#_Toc218584128)

[Section 16a – Supplementary Material – AMSTAR-2 Quality Assessment of the Four Included Reviews (Du et al., 2025, Huang et al., 2025, Li et al., 2025, and Mo et al., 2025) 75](#_Toc218584129)

[Section 17 – Overlap in primary studies included in reviews 85](#_Toc218584130)

[Section 18. Matrix of evidence 87](#_Toc218584131)

Section 2 – SAGER Guidelines

| Recommendations per section of the article | | Page # |
| --- | --- | --- |
| Title and abstract | If only one sex is included in the study, or if the results of the study are to be applied to only one sex or gender, the title and the abstract should specify the sex of animals or any cells, tissues and other material derived from these and the sex and gender of human participants. | NA, all genders included |
| Introduction | Authors should report, where relevant, whether sex and/or gender differences may be expected. | NA, all genders included |
| Methods | Authors should report how sex and gender were considered in the design of the study, whether they ensured adequate representation of males and females, and justify the reasons for any exclusion of males or females. | 6 |
| Results | Where appropriate, data should be routinely presented disaggregated by sex and gender. Sex- and gender-based analyses should be reported regardless of positive or negative outcome. In clinical trials, data on withdrawals and dropouts should also be reported disaggregated by sex. | 10 |
| Discussion | The potential implications of sex and gender on the study results and analyses should be discussed. If a sex and gender analysis was not conducted, the rationale should be given. Authors should further discuss the implications of the lack of such analysis on the interpretation of the results. | 22 |

From: (Heidari S, Babor TF, De Castro P, Tort S, Curno M. Sex and Gender Equity in Research: rationale for the SAGER guidelines and recommended use. Research Integrity and Peer Review. 2016;1: 2. doi:10.1186/s41073-016-0007-6)

# Section 3. Registered in PROSPERO (CRD42023485887)


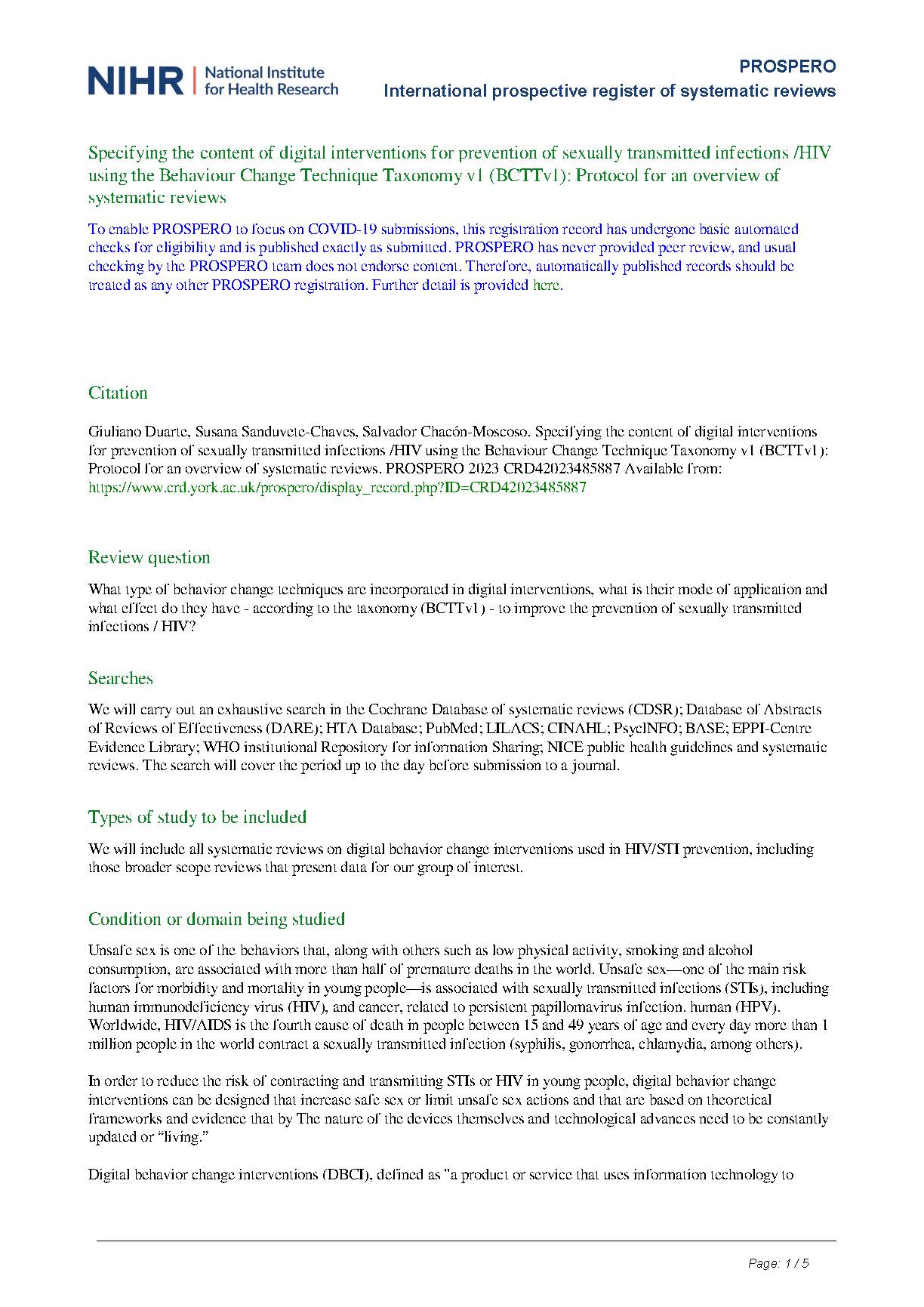


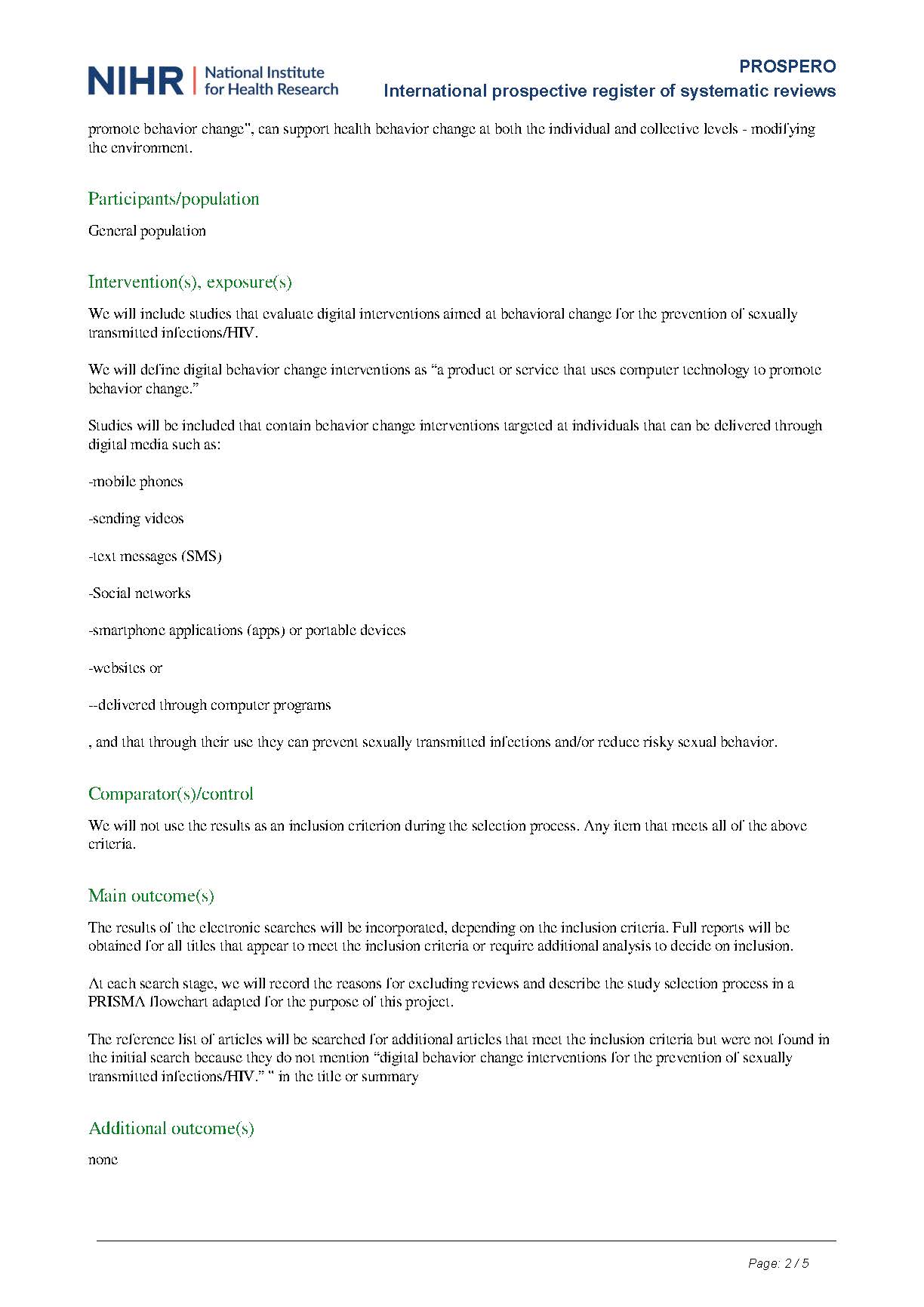


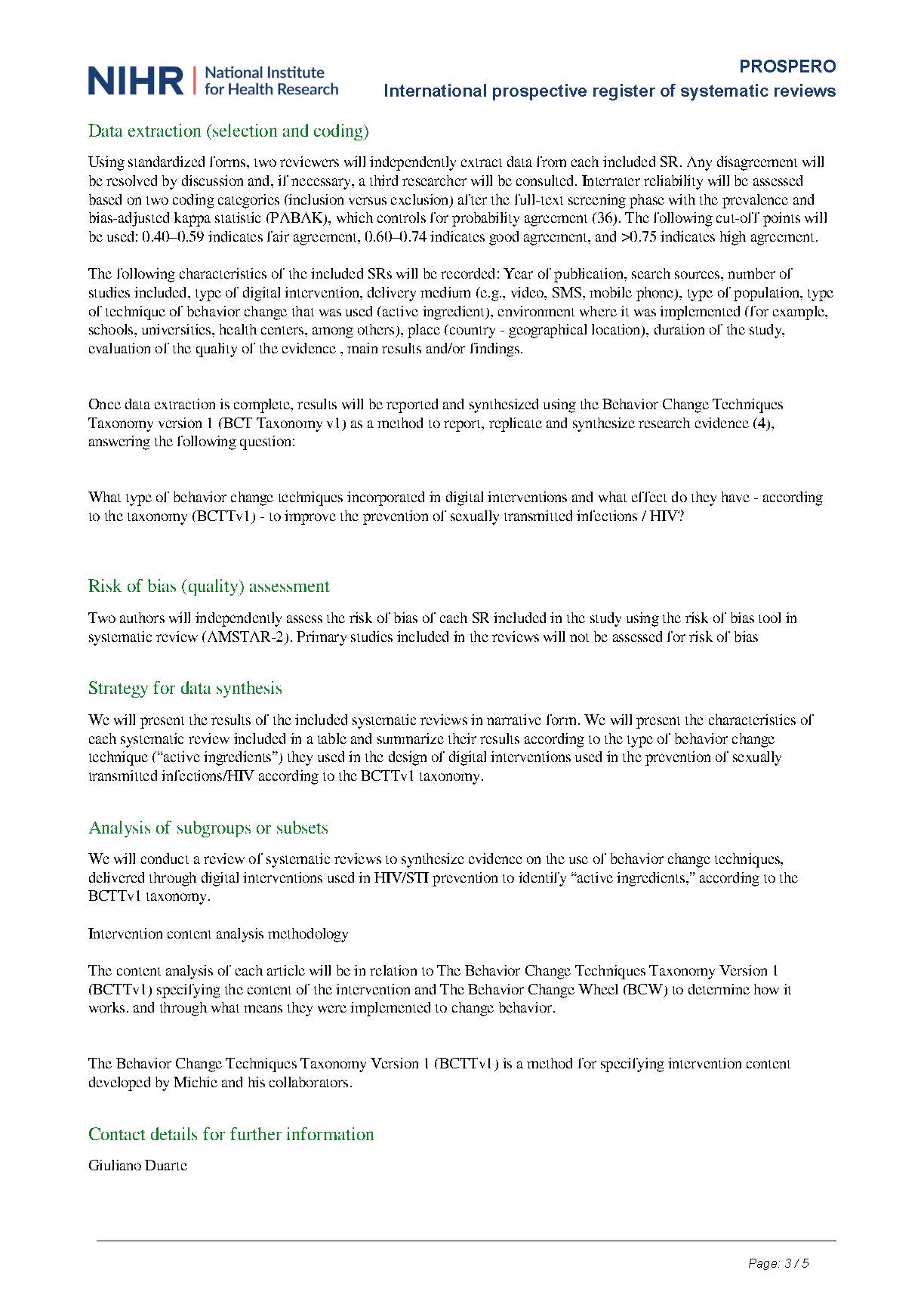


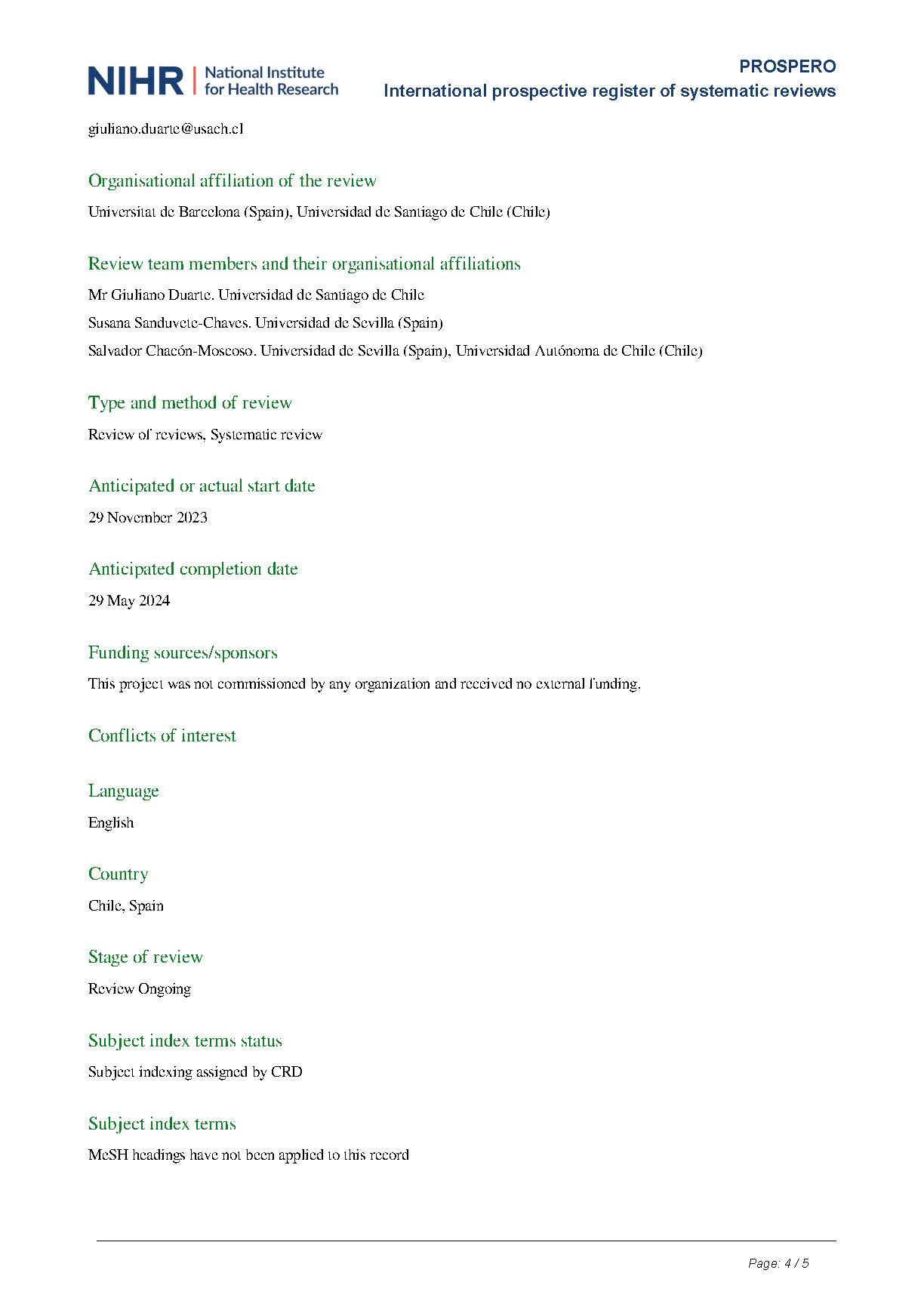

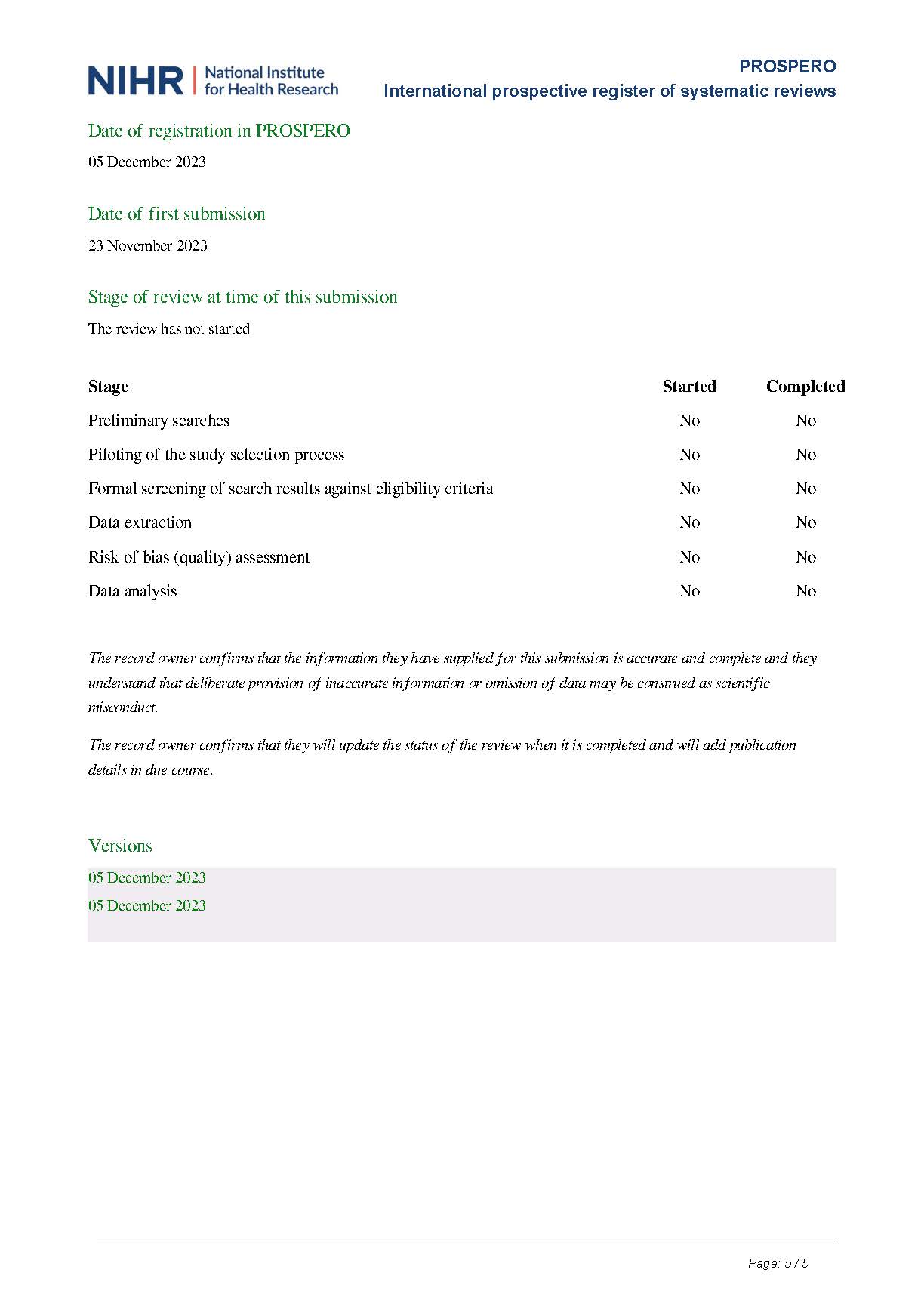


# Section 4 - Identification of studies via other methods (Grey Literature Sources)

In addition to the primary search strategy, supplementary efforts were undertaken to ensure comprehensive identification of relevant studies. These efforts are detailed below:

**1. Manual Review of References**

A manual review of references cited in the included studies was conducted to identify additional relevant literature that may not have been captured in the initial search.

**2. Examination of Related Systematic Reviews**

Systematic reviews that shared at least one study with the included reviews were examined to uncover further studies that could contribute valuable insights to this overview.

**3. Additional Records Identified**

Supplementary searches were performed across various platforms and methods, yielding the following:

Websites (n = 7):

1. National Institute for Health and Care Excellence (NICE): Guideline on reducing sexually transmitted infections. [Accessed from NICE website](https://www.nice.org.uk/guidance/ng221)
2. Google Scholar <https://scholar.google.es/>
3. County Health Rankings and Roadmaps: [Website](https://www.countyhealthrankings.org/)
4. The Lancet Digital Health <https://www.thelancet.com/journals/landig/home>
5. WHO: [http://www.who.int/en/](about:blank)
6. Health & Environmental Research Online (HERO) <https://hero.epa.gov/>
7. PROSPERO <https://www.crd.york.ac.uk/prospero/>

Organizations (n = 1):

-CDC HIV Prevention Research Synthesis Cumulative Database: Focused on digital health interventions. <https://www.cdc.gov/hiv/research/interventionresearch/prs/index.html>

References Provided by Authors (n = 1):

-Duarte et al. 2020: "Effectiveness of digital interventions based on mobile phones for the prevention of sexually transmitted infections: A systematic review."

Contacting Experts (n = 28): A list of experts was contacted for additional input and references, detailed as follows:

| **No.** | **Name** | **Institution/Area of Research** | **Email** |
| --- | --- | --- | --- |
| 1 | Susan Michie | University College of London | [s.michie@ucl.ac.uk](mailto:s.michie@ucl.ac.uk) |
| 2 | Marie Johnston | University of Aberdeen | [m.johnston@abdn.ac.uk](mailto:m.johnston@abdn.ac.uk) |
| 3 | Dominika Kwasnicka | Open Digital Health | [dkwasnicka@gmail.com](mailto:dkwasnicka@gmail.com) |
| 4 | Oscar Castro | Centre for Digital Health Interventions, Singapore-ETH Centre | [oscar.castro@sec.ethz.ch](mailto:oscar.castro@sec.ethz.ch) |
| 5 | Jacob J van den Berg | Brown University | [jacob_vandenberg@brown.edu](mailto:jacob_vandenberg@brown.edu) |
| 6 | WHO, Department of Reproductive Health and Research | Geneva, Switzerland | [anasofiadevasconcelos@gmail.com](mailto:anasofiadevasconcelos@gmail.com) |
| 7 | Sara Bru Garcia | University of Nottingham | [sbrugarcia@gmail.com](mailto:sbrugarcia@gmail.com) |
| 8 | Kelly J Thomas Craig | IBM Watson Health | [kelly.jean.craig@ibm.com](mailto:kelly.jean.craig@ibm.com) |
| 9 | Emmanuel Adebayo | University of Ibadan | [esadebayo@com.ui.edu.ng](mailto:esadebayo@com.ui.edu.ng) |
| 10 | Sareh Keshvardoost | Kerman University of Medical Sciences | [s.dkeshvardoost@kmu.ac.ir](mailto:s.dkeshvardoost@kmu.ac.ir) |
| 11 | Julia Samuelson | WHO, Geneva, Switzerland | [samuelsonj.@who.int](mailto:samuelsonj.@who.int) |
| 12 | Amy G. Cantor | Oregon Health & Science University | [cantor@ohsu.edu](mailto:cantor@ohsu.edu) |
| 13 | Caricia Catalani | University of California, Berkeley | [catalani@berkeley.edu](mailto:catalani@berkeley.edu) |
| 14 | Nicola Luigi Bragazzi | Department of Mathematics and Statistics | [robertobragazzi@gmail.com](mailto:robertobragazzi@gmail.com) |
| 15 | Jennifer Pharr | University of Nevada, Las Vegas | [jennifer.pharr@unlv.edu](mailto:jennifer.pharr@unlv.edu) |
| 16 | José A. Bauermeister | University of Pennsylvania | [bjose@upenn.edu](mailto:bjose@upenn.edu) |
| 17 | Ethan Trey Cardwell | University of Melbourne | [t.cardwell@unimelb.edu.au](mailto:t.cardwell@unimelb.edu.au) |
| 18 | Isaiane da Silva Carvalho | Universidade Federal de Pernambuco | [isaianekarvalho@hotmail.com](mailto:isaianekarvalho@hotmail.com) |
| 19 | Dr. Carole De’glise | Swiss Tropical and Public Health Institute | [cardeglise@yahoo.com](mailto:cardeglise@yahoo.com) |
| 20 | César G. Escobar-Viera | University of Pittsburgh | [escobar-viera@pitt.edu](mailto:escobar-viera@pitt.edu) |
| 21 | Jamie I. Forrest | University of British Columbia | [jforrest@geshealth.com](mailto:jforrest@geshealth.com) |
| 22 | Kate Winskell | Emory University | [swinske@emory.edu](mailto:swinske@emory.edu) |
| 23 | Kathryn Elizabeth Muessig | University of North Carolina | [kmuessig@med.unc.edu](mailto:kmuessig@med.unc.edu) |
| 24 | Galanis P. | National and Kapodistrian University of Athens | [pegalan@nurs.uoa.gr](mailto:pegalan@nurs.uoa.gr) |
| 25 | Bo Zhang | Peking University | [zhangbo0136@pku.edu.cn](mailto:zhangbo0136@pku.edu.cn) |
| 26 | Laura Whiteley | Brown University | [laura_whiteley@brown.edu](mailto:laura_whiteley@brown.edu) |
| 27 | Dr. Sima Berendes | London School of Hygiene & Tropical Medicine | [sima.berendes@lshtm.ac.uk](mailto:sima.berendes@lshtm.ac.uk) |
| 28 | Jennifer Pharr | University of Nevada, Las Vegas | [jennifer.pharr@unlv.edu](mailto:jennifer.pharr@unlv.edu) |

Citation Searching (n = 321): Citation searches were conducted to track references that cited key studies included in the overview.

| Citation Searching (n = 321): Citation searches were conducted to track references that cited key studies included in the overview. | | |
| --- | --- | --- |
| Nro | **Author/ Year** | **Reference** |
| 1 | Bauermeister 2015 | Bauermeister JA, Pingel ES, Jadwin-Cakmak L, Harper GW, Horvath K, Weiss G, Dittus P. Acceptability and preliminary efficacy of a tailored online HIV/STI testing intervention for young men who have sex with men: the Get Connected! program. AIDS Behav 2015; 19:1860–1874. |
| 2 | Billings 2015 | Billings DW, Leaf Sl Spencer J, Crenshaw T, Brockington S, Dalal RS. A randomized trial to evaluate the efficacy of a web-based HIV behavioral intervention for high-risk African American women. AIDS Behav 2015; 19:1263–1274. |
| 3 | Bowen 2007 | Bowen A, Horvath K, Williams M. A randomized control trial of Internet-delivered HIV prevention targeting rural MSM. Health Educ Res 2007; 22:120–127. |
| 4 | Bull 2009 | Bull SS, Pratte K, Whitesell N, Rietmeijer C, McFarlane M. Effects of an internet-based intervention for HIV prevention: the Youthnet trials. AIDS Behav 2009; 13:474–487. |
| 5 | Calderon 2013 | Calderon Y, Cowan E, Leu CS, Brusalis C, Rhee JY, Nickerson J, et al.. A human immunodeficiency virus posttest video to increase condom use among adolescent emergency department patients. J Adolesc Health 2013; 53:79–84. |
| 6 | Carpenter 2010 | Carpenter KM, Stoner SA, Mikko AN, Dhanak LP, Parsons JT. Efficacy of a web-based intervention to reduce sexual risk in men who have sex with men. AIDS Behav 2010; 14:549–557. |
| 7 | Christensen 2013 | Christensen JL, Miller LC, Appleby PR, Corsbie-Massay C, Godoy CG, Marsella SC, Read SJ. Reducing shame in a game that predicts HIV risk reduction for young adult MSM: a randomized trial delivered nationally over the Web. J Int AIDS Soc 2013; 16: Suppl 2: 18716. |
| 8 | Davidovich 2006 | Davidovich U, de Wit J, Stroebe W. Using the Internet to reduce risk of HIV-infection in steady relationships: A randomized controlled trial of a tailored intervention for gay men. In Liaisons dangereuses: HIV risk behavior prevention in steady gay relationships. Amsterdam: Roel & Uigeefprojecten. 2006:95–122. |
| 9 | Di Noia 2004 | Di Noia J, Schinke SP, Pena JB, Schwinn TM. Evaluation of a brief computer-mediated intervention to reduce HIV risk among early adolescent females. J Adolesc Health 2004; 35:62–64 |
| 10 | Evans 2000 | Evans AE, Edmundson-Drane EW, Harris KK. Computer-assisted instruction: an effective instructional method for HIV prevention education?. J Adolesc Health 2000; 26:244–251. |
| 11 | Festinger 2016 | Festinger DS, Dugosh Kl, Kurth Ae, Metzger DS. Examining the efficacy of a computer facilitated HIV prevention tool in drug court. Drug Alcohol Depend 2016; 162:44–50. |
| 12 | Fiellin 2017 | Fiellin LE, Hieftje KD, Pendergrass TM, Kyriakides TC, Duncan LR, Dziura JD, Sawyer BG. Video game intervention for sexual risk reduction in minority adolescents: randomized controlled trial. J Med Internet Res 2017; 19:e314. |
| 13 | Gilbert 2008 | Gilbert P, Ciccarone D, Gansky Sa, Bangsberg DR, Clanon K, McPhee SJ, et al.. Interactive ‘Video Doctor’ counseling reduces drug and sexual risk behaviors among HIV-positive patients in diverse outpatient settings. PLoS One 2008; 3:e1988. |
| 14 | Hightow-Weidman 2012 | Hightow-Weidman LB, Pike E, Fowler B, Matthews DM, Kibe J, McCoy R, Adimora AA. Feasibility and acceptability of delivering an internet intervention to young Black men who have sex with men. AIDS Care 2012; 24:910–920 |
| 15 | Ito 2008 | Ito KE, Kalyanaraman S, Ford CA, Brown JD, Miller WC. Let's talk about sex’: pilot study of an interactive CD-ROM to prevent HIV/STIS in female adolescents. AIDS Educ Prevent 2008; 1:78–89. |
| 16 | Jenkins 2000 | Jenkins PR, Jenkins RA, Nannis ED, McKee KT, Jr, Temoshok LR. Reducing risk of sexually transmitted disease (STD) and human immunodeficiency virus infection in a military STD clinic: evaluation of a randomized preventive intervention trial. Clin Infect Dis 2000; 30:730–735. |
| 17 | Kiene 2006 | Kiene SM, Barta WD. A brief individualized computer-delivered sexual risk reduction intervention increases HIV/AIDS preventive behavior. J Adolesc Health 2006; 39:404–410. |
| 18 | Klein 2013 | Klein CH, Lomonaco CG, Pavlescak R, Card JJ. WiLLOW: reaching HIV-positive African-American women through a computer-delivered intervention. AIDS Behav 2013; 17:3013–3023. |
| 19 | Kurth 2014 | Kurth AE, Spielberg F, Cleland CM, Lambdin B, Bangsberg DR, Frick PA, et al.. Computerized counseling reduces HIV-1 viral load and sexual transmission risk: findings from a randomized controlled trial. J Acquir Immune Defic Syndr 2014; 65:611–620. |
| 20 | Leiby 2016 | Leiby K, Connor A, Tsague L, et al.. The impact of SMS-based interventions on VMMC uptake in Lusaka province, Zambia: a randomized controlled trial. J Acquir Immune Defic Syndr 2016; 72: Suppl 4: S264–S272. |
| 21 | Marsch 2011 | Marsch LA, Grabinski MJ, Bickel WK, Desrosiers A, Guarino H, Muehlbach B, et al.. Computer-assisted HIV prevention for youth with substance use disorders. Subst Use Misuse 2011; |
| 22 | Marsch 2015 | Marsch LA, Guarino H, Grabinski MJ, Dillingham ET, Xie H, Crosier BS. Comparative effectiveness of web-based vs. educator-delivered HIV prevention for adolescent substance users: a randomized, controlled trial. J Subst Abuse Treat 2015; 59:30–37. |
| 23 | McKinstry 2017 | McKinstry LA, Zerbe A, Hanscom B, Farrior J, Kurth AE, Stanton J, et al.. A randomized-controlled trial of computer-based prevention counseling for HIV-positive persons. J AIDS Clin Res 2017; 8:714. |
| 24 | Merchant 2011 | Merchant RC, Clark MA, Langan TJ, Mayer KH, Seage GR, III, DeGruttola VG. Can computer-based feedback improve emergency department patient uptake of rapid HIV screening?. Ann Emerg Med 2011; 58: 1 Suppl 1: S114.e1–S119.e2. |
| 25 | Milam 2016 | Milam J, Morris S, Jain S, Sun X, Dubé MP, Daar ES, et al.. Randomized controlled trial of an internet application to reduce HIV transmission behavior among HIV infected men who have sex with men. AIDS Behav 2016; 20:1173–1181. |
| 26 | Perry 1991 | Perry S, Fishman B, Jacobsberg L, Young J, Frances A. Effectiveness of psychoeducational interventions in reducing emotional distress after human immunodeficiency virus antibody testing. Arch Gen Psychiatry 1991; 48:143–147. |
| 27 | Read 2006 | Read S, Miller L, Appleby P, Nwosu ME, Reynaldo S, Lauren A, Putcha A. Socially optimized learning in a virtual environment: reducing risky sexual behavior among men who have sex with men. Hum Comm Res 2006; 32:1–34. |
| 28 | Rosser 2010 | Rosser BR, Oakes JM, Konstan J, Hooper S, Horvath KJ, Danilenko G, et al.. Reducing HIV risk behavior of men who have sex with men through persuasive computing: results of the Men's INTernet Study-II. AIDS 2010; 24:2099–2107 |
| 29 | Schonnesson 2016 | Schonnesson LN, Bowen AM, Williams ML. Project SMART: preliminary results from a test of the efficacy of a Swedish internet-based HIV risk-reduction intervention for men who have sex with men. Arch Sex Behav 2016; 45:1501–1511. |
| 30 | Ybarra 2013 | Ybarra ML, Bull SS, Prescott TL, Korchmaros JD, Bangsberg DR, Kiwanuka JP, et al.. Adolescent abstinence and unprotected sex in CyberSenga, an Internet-Based HIV Prevention Program: randomized clinical trial of efficacy. PLOS One 2013; 8:e70083. |
| 31 | de Tolly 2012 | de Tolly K, Skinner D, Nembaware V, et al. Investigation into the use of short message services to expand uptake of human immunodeficiency virus testing, and whether content and dosage have impact. Telemedicine and e-Health 2012;18:18–23. |
| 32 | Delamere 2006 | Delamere S, Dooley S, Harrington L. P92 - Safer sex text messages: Evaluating a health education intervention in an adolescent population. Sexually transmitted infections 2006;82:A27. |
| 33 | Downing 2013 | Downing SG, Cashman C, McNamee H, et al. Increasing Chlamydia test of re-infection rates using SMS reminders and incentives. Sex Transm Infect 2013;89:16–19. |
| 34 | Free 2016 | Free C, McCarthy O, French RS, et al. Can text messages increase safer sex behaviours in young people? intervention development and pilot randomised controlled trial. Health Technol Assess 2016;20:1–82. |
| 35 | Gold 2011 | Gold J, Aitken CK, Dixon HG, et al. A randomised controlled trial using mobile advertising to promote safer sex and sun safety to young people. Health Educ Res 2011;26:782–94. |
| 36 | Govender 2019 | Govender K, Beckett S, Masebo W, et al. Effects of a short message service (SMS) intervention on reduction of HIV risk behaviours and improving HIV testing rates among populations located near roadside wellness clinics: a cluster randomised controlled trial in South Africa, Zimbabwe and Mozambique. AIDS Behav 2019;23:3119–28. |
| 37 | Kelvin 2019 | Kelvin EA, George G, Kinyanjui S, et al. Announcing the availability of oral HIV self-test kits via text message to increase HIV testing among hard-to-reach truckers in Kenya: a randomized controlled trial. BMC Public Health 2019;19:7. |
| 38 | Kelvin 2019 | Kelvin EA, George G, Mwai E, et al. A randomized controlled trial to increase HIV testing demand among female sex workers in Kenya through announcing the availability of HIV Self-testing via text message. AIDS Behav 2019;23:116–25. |
| 39 | Lim 2012 | Lim MSC, Hocking JS, Aitken CK, et al. Impact of text and email messaging on the sexual health of young people: a randomised controlled trial. J Epidemiol Community Health 2012;66:69–74. |
| 40 | Mimiaga 2017 | Mimiaga MJ, Thomas B, Biello K, et al. A pilot randomized controlled trial of an integrated In-person and mobile phone delivered counseling and text messaging intervention to reduce HIV transmission risk among male sex workers in Chennai, India. AIDS Behav 2017;21:3172–81. |
| 41 | Mugo 2016 | Mugo PM, Wahome EW, Gichuru EN, et al. Effect of text message, phone call, and In-Person appointment reminders on uptake of repeat HIV testing among outpatients screened for acute HIV infection in Kenya: a randomized controlled trial. PLoS One 2016;11:e0153612. |
| 42 | Nielsen 2021 | Nielsen AM, De Costa A, Gemzell-Danielsson K, et al. The MOSEXY trial: mobile phone intervention for sexual health in youth-a pragmatic randomised controlled trial to evaluate the effect of a smartphone application on sexual health in youth in Stockholm, Sweden. Sex Transm Infect 2021;97:141–6. |
| 43 | Parkes-Ratanshi 2018 | Parkes-Ratanshi R, Mbazira KJ, Nakku-Joloba E. Congenital syphilis prevention in Uganda; a randomised trial of three different approaches to partner notification for pregnant women: override. HIV Medicine 2018;19:S132. |
| 44 | Reback 2019 | Reback CJ, Fletcher JB, Swendeman DA, et al. Theory-Based Text-Messaging to reduce methamphetamine use and HIV sexual risk behaviors among men who have sex with men: automated unidirectional delivery outperforms bidirectional peer interactive delivery. AIDS Behav 2019;23:37–47. |
| 45 | Rokicki 2017 | Rokicki S, Cohen J, Salomon JA, et al. Impact of a Text-Messaging program on adolescent reproductive health: a Cluster–Randomized trial in Ghana. Am J Public Health 2017;107:298–305. |
| 46 | Suffoletto 2013 | Suffoletto B, Akers A, McGinnis KA, et al. A sex risk reduction text-message program for young adult females discharged from the emergency department. Journal of Adolescent Health 2013;53:387–93. |
| 47 | Tang 2018 | Tang W, Wei C, Cao B, et al. Crowdsourcing to expand HIV testing among men who have sex with men in China: a closed cohort stepped wedge cluster randomized controlled trial. PLoS Med 2018;15:e1002645. |
| 48 | Trent 2019 | Trent M, Perin J, Gaydos CA, et al. Efficacy of a Technology-Enhanced community health nursing intervention vs standard of care for female adolescents and young adults with pelvic inflammatory disease. JAMA Network Open 2019;2:e198652. |
| 49 | Ybarra 2017 | Ybarra ML, Prescott TL, Phillips GL, et al. Pilot RCT results of an mHealth HIV prevention program for sexual minority male adolescents. Pediatrics 2017;140:e20162999. |
| 50 | Young 2013 | Young SD, Cumberland WG, Lee SJ. Social networking technologies as an emerging tool for HIV prevention: a cluster randomized trial. Ann Intern Med 2013;159:318–24. |
| 51 | Zhu X 2019 | Zhu X, Zhang W, Operario D, et al. Effects of a mobile health intervention to promote HIV Self-testing with MSM in China: a randomized controlled trial. AIDS Behav 2019;23:3129–39. |
| 52 | Jones 2013 | Jones R, Hoover DR, Lacroix LJ. A randomized controlled trial of soap opera videos streamed to smartphones to reduce risk of sexually transmitted human immunodeficiency virus (HIV) in young urban African American women. Nurs Outlook. 2013;61(4):205–15. e203. |
| 53 | Lim 2010 | Lim MSC, Sacks-Davis R, Aitken CK, Hocking JS, Hellard ME. Randomised controlled trial of paper, online and SMS diaries for collecting sexual behaviour information from young people. J Epidemiol Community Health. 2010;64:885–9. doi:10.1136/jech.2008.085316. |
| 54 | Odeny 2012 | Odeny TA, Bailey RC, Bukusi EA, Simoni JM, Tapia KA, Yuhas K, et al. Text messaging to improve attendance at post-operative clinic visits after adult male circumcision for HIV prevention: a randomized controlled trial. PLoS One. 2012;7(9):e43832. doi:10.1371/journal.pone.0043832. |
| 55 | Odeny 2014 | Odeny TA, Bailey RC, Bukusi EA, Simoni JM, Tapia KA, Yuhas K, Holmes KK, McClelland RS. Effect of text messaging to deter early resumption of sexual activity after male circumcision for HIV prevention: a randomized controlled trial. J Acquir Immune Defic Syndr. 2014;65(2):e50–7. doi:10.1097/QAI. 0b013e3182a0a050. |
| 56 | Shahkolahi 2013 | Shahkolahi MM, Wilson A, Zucker L. Mobile-application-based HIV intervention: an opt-in approach to promote rapid HIV screening in an inner-city emergency department. Ann Emerg Med. 2013;1:S95. |
| 57 | Biggs 2016 | Biggs K, Walsh J, Ooi C. Deadly liver mob: opening the door improving sexual health pathways for Aboriginal people in Western Sydney. Sex Health 2016; 13(5): 457–64. doi:10.1071/ SH15176 |
| 58 | Bourne 2011 | Bourne C, Knight V, Guy R, Wand H, Lu H, McNulty A. Short message service reminder intervention doubles sexually transmitted infection/HIV re-testing rates among men who have sex with men. Sex Transm Infect 2011; 87(3): 229–31. doi:10.1136/sti.2010. 048397 |
| 59 | Burton 2014 | Burton J, Brook G, McSorley J, Murphy S. The utility of short message service (SMS) texts to remind patients at higher risk of STIs and HIV to reattend for testing: a controlled before and after study. Sex Transm Infect 2014; 90: 11–3. doi:10.1136/sextrans2013-051228 |
| 60 | Guy 2013 | Guy R, Wand H, Knight V, Keningsberg A, Read P, McNulty AM. SMS reminders improve re-screening in women and heterosexual men with chlamydia infection at Sydney Sexual Health Centre: a before-and-after study. Sex Transm Infect 2013; 89: 11–5. doi:10. 1136/sextrans-2011-050370 |
| 61 | Ingersoll 2015 | Ingersoll KS, Dillingham RA, Hettema JE, Conaway M, Freeman J, Reynolds G, Hosseinbor S. Pilot RCT of bidirectional text messaging for ART adherence among nonurban substance users with HIV. Health Psychol 2015; 34: 1305–15. doi:10.1037/hea0000295 |
| 62 | Malotte 2004 | Malotte CK, Ledsky R, Hogben M, Larro M, Middlestadt S, St Lawrence JS, Olthoff G, Settlage RH, Van Devanter NL, GCAP Study Group. Comparison of methods to increase repeat testing in persons treated for gonorrhoea and/or chlamydia at public sexually transmitted disease clinics. Sex Transm Dis 2004; 31(11): 637–42. doi:10.1097/01.olq.0000143083.38684.9d |
| 63 | Norton 2014 | Norton BL, Person AK, Castillo C, Pastrana C, Subramanian M, Stout JE. Barriers to using text message appointment reminders in an HIV clinic. Telemed J E Health 2014; 20(1): 86–9. doi:10.1089/tmj.2012. 0275 |
| 64 | Nyatsanza 2016 | Nyatsanza F, McSorley J, Murphy S, Brook G. ‘It’s all in the message’: the utility of personalised short message service (SMS) texts to remind patients at higher risk of STIs and HIV to reattend for testing – a repeat before and after study. Sex Transm Infect 2016; 92: 393–5. doi:10.1136/sextrans-2015-052216 |
| 65 | Rana 2016 | Rana AI, van den Berg JJ, Lamy E, Beckwith CG. Using a mobile health intervention to support HIV treatment adherence and retention among patients at risk for disengaging with care. AIDS Patient Care STDS 2016; 30(4): 178–84. doi:10.1089/apc.2016. 0025 |
| 66 | Rutland 2012 | Rutland E, Roe H, Weaver A. O11 Health promotional messages in short message service (SMS) follow-up of GU medicine clinic defaulters; a tool to improve subsequent attendance rates? Sex Transm Infect 2012; 88: A4–5. doi:10.1136/sextrans-2012050601a.11 |
| 67 | Tanner 2018 | Tanner AE, Song EY, Mann-Jackson L, Alonzo J, Schafer K, Ware S, Garcia JM, Arellano Hall E, Bell JC, Van Dam CN, Rhodes SD. Preliminary impact of the weCare Social Media intervention to support health for young men who have sex with men and transgender women with HIV. AIDS Patient Care STDS 2018; 32(11): 450–8. doi:10.1089/apc.2018.0060 |
| 68 | Zou 2013 | Zou H, Fairley CK, Guy R, Bilardi J, Bradshaw CS, Garland SM, Sze JK, Afrizal A, Chen MY. Automated, computer generated reminders and increased detection of gonorrhoea, chlamydia and syphilis in men who have sex with men. PLoS One 2013; 8(4): e61972. doi:10.1371/journal.pone.0061972 |
| 69 | Rinehart 2020 | Rinehart DJ, Leslie S, Durfee MJ, et al. Acceptability and efficacy of a sexual health Texting intervention designed to support adolescent females. Acad Pediatr 2020;20:475–84. |
| 70 | Agarwal 2015 | Agarwal A, Hamdallah M, Swain SN, Mukherjee S, Singh N, Mahapatra S, et al. Implementation of a Confidential Helpline for Men Having Sex With Men in India. JMIR mHealth and uHealth. 2015; 3(1) |
| 71 | Odeny 2014 | Odeny TA, Bukusi EA, Cohen CR, Yuhas K, Camlin CS, McClelland RS. Texting improves testing: a randomized trial of two-way SMS to increase postpartum prevention of mother-to-child transmission retention and infant HIV testing. AIDS. 2014; 28(15):2307–12. [PubMed: 25313586] |
| 72 | Udeagu 2014 | Udeagu C-CN, Bocour A, Shah S, Ramos Y, Gutierrez R, Shepard CW. Bringing HIV Partner Services Into the Age of Social Media and Mobile Connectivity. Sexually transmitted diseases. 2014; 41(10):631–6. [PubMed: 25211262] |
| 73 | Zou 2013 | Zou H, Wu Z, Yu J, Li M, Ablimit M, Li F, et al. Internet-facilitated, voluntary counseling and testing (VCT) clinic-based HIV testing among men who have sex with men in China. PLoS One. 2013; 8(2):e51919. [PubMed: 23418417 |
| 74 | Bertozzi 2018 | Bertozzi E, Bertozzi-Villa A, Kulkarni P, Sridhar A. Collecting family planning intentions and providing reproductive health information using a tablet-based video game in India. Gates Open Res. 2018;2:20. doi: 10.12688/gatesopenres.12818.2. |
| 75 | Chib 2011 | Chib A. Handbook of Research On Improving Learning And Motivation Through Educational Games. Hershey, Pennsylvania: IGI Global; 2011. Promoting sexual health education via gaming: evidence from the barrios of Lima, Peru; pp. 895–912. |
| 76 | Chu 2015 | Chu SKW, Kwan AC, Reynolds R, Mellecker RR, Tam F, Lee G, Hong A, Leung CY. Promoting Sex Education Among Teenagers Through an Interactive Game: Reasons for Success and Implications. Games Health J. 2015 Jun;4(3):168–74. doi: 10.1089/g4h.2014.0059. |
| 77 | Escobar-Chaves 2011 | Escobar-Chaves S, Shegog R, Moscoso-Alvarez M, Markham C, Tortolero-Luna G, Peskin M, Tortolero S. Cultural tailoring and feasibility assessment of a sexual health middle school curriculum: a pilot test in Puerto Rico. J Sch Health. 2011 Aug;81(8):477–84. doi: 10.1111/j.1746-1561.2011.00617.x. |
| 78 | Gariepy 2018 | Gariepy AM, Hieftje K, Pendergrass T, Miller E, Dziura JD, Fiellin LE. Development and Feasibility Testing of a Videogame Intervention to Reduce High-Risk Sexual Behavior in Black and Hispanic Adolescents. Games Health J. 2018 Dec;7(6):393–400. doi: 10.1089/g4h.2017.0142. |
| 79 | Haruna 2018 | Haruna H, Hu X, Chu S, Mellecker R, Gabriel G, Ndekao P. Improving Sexual Health Education Programs for Adolescent Students through Game-Based Learning and Gamification. IJERPH. 2018 Sep 17;15(9):2027. doi: 10.3390/ijerph15092027. |
| 80 | Markham 2012 | Markham CM, Tortolero SR, Peskin MF, Shegog R, Thiel M, Baumler ER, Addy RC, Escobar-Chaves SL, Reininger B, Robin L. Sexual risk avoidance and sexual risk reduction interventions for middle school youth: a randomized controlled trial. J Adolesc Health. 2012 Mar;50(3):279–88. doi: 10.1016/j.jadohealth.2011.07.010. |
| 81 | Oliveira 2016 | Oliveira RNGD, Gessner R, Souza VD, Fonseca RMGSD. Limits and possibilities of an online game for building adolescents' knowledge of sexuality. Cien Saude Colet. 2016 Aug;21(8):2383–92. doi: 10.1590/1413-81232015218.04572016. |
| 82 | Peskin 2015 | Peskin MF, Shegog R, Markham CM, Thiel M, Baumler ER, Addy RC, Gabay EK, Emery ST. Efficacy of It's Your Game-Tech: A Computer-Based Sexual Health Education Program for Middle School Youth. J Adolesc Health. 2015 May;56(5):515–21. doi: 10.1016/j.jadohealth.2015.01.001 |
| 83 | Peskin 2019 | Peskin MF, Coyle KK, Anderson PM, Laris BA, Glassman JR, Franks HM, Thiel MA, Potter SC, Unti T, Edwards S, Johnson-Baker K, Cuccaro PM, Diamond P, Markham CM, Shegog R, Baumler ER, Gabay EK, Emery ST. Replication of It's Your Game…Keep It Real! in Southeast Texas. J Prim Prev. 2019 Jun;40(3):297–323. doi: 10.1007/s10935-019-00549-0.10.1007/s10935-019-00549-0 |
| 84 | Potter 2016 | Potter SC, Coyle KK, Glassman JR, Kershner S, Prince MS. in South Carolina: A Group Randomized Trial Evaluating the Replication of an Evidence-Based Adolescent Pregnancy and Sexually Transmitted Infection Prevention Program. Am J Public Health. 2016 Sep;106(S1):S60–S69. doi: 10.2105/ajph.2016.303419 |
| 85 | Rohrbach 2019 | Rohrbach LA, Donatello RA, Moulton BD, Afifi AA, Meyer KI, De Rosa CJ. Effectiveness Evaluation of It's Your Game: Keep It Real, a Middle School HIV/Sexually Transmitted Infection/Pregnancy Prevention Program. J Adolesc Health. 2019 Mar;64(3):382–389. doi: 10.1016/j.jadohealth.2018.09.021.S1054-139X(18)30436-1 |
| 86 | Shegog 2014 | Shegog R, Peskin MF, Markham C, Thiel M, Karny E, Addy RC, Johnson KA, Tortolero S. It's Your Game-Tech: Toward Sexual Health in the Digital Age. Creat Educ. 2014 Aug;5(15):1428–1447. doi: 10.4236/ce.2014.515161. |
| 87 | Shegog 2017 | Shegog R, Craig Rushing S, Gorman G, Jessen C, Torres J, Lane TL, Gaston A, Revels TK, Williamson J, Peskin MF, D'Cruz Jina, Tortolero S, Markham CM. NATIVE-It's Your Game: Adapting a Technology-Based Sexual Health Curriculum for American Indian and Alaska Native youth. J Prim Prev. 2017 Apr;38(1-2):27–48. doi: 10.1007/s10935-016-0440-9.10.1007/s10935-016-0440-9 |
| 88 | Shegog 2021 | Shegog R, Armistead L, Markham C, Dube S, Song H, Chaudhary P, Spencer A, Peskin M, Santa Maria D, Wilkerson JM, Addy R, Tortolero Emery S, McLaughlin J. A Web-Based Game for Young Adolescents to Improve Parental Communication and Prevent Unintended Pregnancy and Sexually Transmitted Infections (The Secret of Seven Stones): Development and Feasibility Study. JMIR Serious Games. 2021 Jan 27;9(1):e23088. doi: 10.2196/23088. |
| 89 | Tortolero 2010 | Tortolero SR, Markham CM, Peskin MF, Shegog R, Addy RC, Escobar-Chaves SL, Baumler ER. It's Your Game: Keep It Real: delaying sexual behavior with an effective middle school program. J Adolesc Health. 2010 Feb;46(2):169–79. doi: 10.1016/j.jadohealth.2009.06.008. |
| 90 | Winskell 2018 | Winskell K, Sabben G, Akelo V, Ondeng'e K, Obong'o C, Stephenson R, Warhol D, Mudhune V. A Smartphone Game-Based Intervention (Tumaini) to Prevent HIV Among Young Africans: Pilot Randomized Controlled Trial. JMIR Mhealth Uhealth. 2018 Aug 01;6(8):e10482. doi: 10.2196/10482. |
| 91 | Bull 2012 | Bull S, Levine D, Black S, et al. Social mediaYdelivered sexual health intervention: A cluster randomized controlled trial. Am J Prev Med 2012; 43:467Y474. |
| 92 | Danielson 2013 | Danielson C, McCauley J, Jones A, et al. Feasibility of delivering evidenced-based HIV/STI prevention programming to a community sample of African American teen girls via the Internet. AIDS Educ Prev 2013; 25:394Y404. |
| 93 | Gold 2011 | Gold J, Lim M, Hocking J, et al. Determining the impact of text messaging for sexual health promotion to young people. Sex Transm Dis 2011; 38:247Y252. |
| 94 | Huang and Hung 2009 | Huang Y, Hung C. The effect of health education through the Internet on university female students’ hepatitis B knowledge and cognition. J Clin Nurs 2009; 18:3342Y3348. |
| 95 | Jones 2012 | Jones K, Baldwin K, Lewis P. The potential influence of a social media intervention on risky sexual behavior and chlamydia incidence. J Community Health Nurs 2012; 29:106Y120. |
| 96 | Juzang 2011 | Juzang I, Fortune T, Black S, et al. A pilot programme using mobile phones for HIV prevention. J Telemed Telecare 2011; 17:150Y153. |
| 97 | Markham 2014 | Markham C, Shegog R, Leonard A. +CLICK: Harnessing Web-based training to reduce secondary transmission among HIV-positive youth. AIDS Care 2009; 21:622Y631 |
| 98 | Roberto 2007 | Roberto A, Zimmerman R, Carlyle K, et al. A computer-based approach to preventing pregnancy, STD, and HIV in rural adolescents. J Health Commun 2007; 12:53Y76. |
| 99 | Van den Elshout MAM 2021 | van den Elshout MAM, Hoornenborg E, Achterbergh RCA, et al. Improving adherence to daily preexposure prophylaxis among MSM in Amsterdam by providing feedback via a mobile application. AIDS. 2021;35(11):1823–1834. |
| 100 | Colson 2020 | Colson PW, Franks J, Wu Y, et al. Adherence to pre-exposure prophylaxis in black men who have sex with men and transgender women in a community setting in Harlem, NY. AIDS Behav. 2020;24(12):34363455. |
| 101 | Moore Dj 2018 | Moore DJ, Jain S, Dub e MP, et al. Randomized controlled trial of daily text messages to support adherence to preexposure prophylaxis in individuals at risk for human immunodeficiency virus: the TAPIR study. Clin Infect Dis. 2018;66(10):1566–1572. |
| 102 | Fuchs JD 2018 | Fuchs JD, Stojanovski K, Vittinghoff E, et al. A mobile health strategy to support adherence to antiretroviral preexposure prophylaxis. AIDS Patient Care STDS. 2018;32(3):104–111. |
| 103 | Mitchell JT 2018 | Mitchell JT, LeGrand S, Hightow-Weidman LB, et al. Smartphonebased contingency management intervention to improve pre-exposure prophylaxis adherence: pilot trial. JMIR Mhealth Uhealth. 2018;6(9): e10456. |
| 104 | Liu 2019 | Liu AY, Vittinghoff E, von Felten P, et al. Randomized controlled trial of a mobile health intervention to promote retention and adherence to preexposure prophylaxis among young people at risk for human immunodeficiency virus: the EPIC study. Clin Infect Dis. 2019;68 (12):2010–2017. |
| 105 | Whiteley 2021 | Whiteley L, Craker L, Haubrick KK, et al. The impact of a mobile gaming intervention to increase adherence to pre-exposure prophylaxis. AIDS Behav. 2021;25(6):1884–1889. |
| 106 | Mitchel JT 2022 | Mitchell JT, Burns CM, Atkinson B, et al. Feasibility, acceptability, and preliminary effficacy of a gamified mobile health contingency management intervention for PrEP adherence among Black MSM. AIDS Behav. 2022;26(10):3311–3324. |
| 107 | Weitzman PF 2021 | Weitzman PF, Zhou Y, Kogelman L, Rodarte S, Vicente SR, Levkoff SE. mHealth for pre-exposure prophylaxis adherence by young adult men who have sex with men. Mhealth. 2021;7:44. |
| 108 | Gerend 2021 | Gerend, M. A., Madkins, K., Crosby, S., Korpak, A. K., Phillips, G. L., Bass, M., ... Mustanski, B. (2021). Evaluation of a text messaging-based human papillomavirus vaccination intervention for young sexual minority men: Results from a pilot randomized controlled trial. Annals of Behavioral Medicine, 55, 321–332. |
| 109 | Tull 2019 | Tull, F., Borg, K., Knott, C., Beasley, M., Halliday, J., Faulkner, N., ... Bragge, P. (2019). Short message service reminders to parents for increasing adolescent human papillomavirus vaccination rates in a secondary school vaccine program: A randomized control trial. Journal of Adolescent Health, 65, 116–123. |
| 110 | Keeshin & Feinberg 2017 | Keeshin, S. W., & Feinberg, J. (2017). Text message reminder-recall to increase HPV immunization in young HIV-1-infected patients. Journal of the International Association of Providers of AIDS Care, 16, 110–113. |
| 111 | Rand 2017 | Rand, C. M., Vincelli, P., Goldstein, N. P. N., Blumkin, A., & Szilagyi, P. G. (2017). Effects of phone and text message reminders on completion of the human papillomavirus vaccine series. Journal of Adolescent Health, 60, 113–119. |
| 112 | Rand 2015 | Rand, C. M., Brill, H., Albertin, C., Humiston, S. G., Schaffer, S., Shone, L. P., ...Szilagyi, P. G. (2015). Effectiveness of centralized text message reminders on human papillomavirus immunization coverage for publicly insured adolescents. Journal of Adolescent Health, 56, S17–S20. |
| 113 | Matheson 2014 | Matheson, E. C., Derouin, A., Gagliano, M., Thompson, J. A., & Blood-Siegfried, J. (2014). Increasing HPV vaccination series completion rates via text message reminders. Journal of Pediatric Health Care, 28, e35–e39. |
| 114 | Kharbanda 2011 | Kharbanda, E. O., Stockwell, M. S., Fox, H. W., Andres, R., Lara, M., & Rickert, V. I. (2011). Text message reminders to promote human papillomavirus vaccination. Vaccine, 29, 2537–2541. |
| 115 | Bowen 2008 | Bowen AM, Williams ML, Daniel CM, Clayton S. Internet based HIV prevention research targeting rural MSM: feasibility, acceptability, and preliminary efficacy. J Behav Med. 2008;31(6):463–77. |
| 116 | Lau 2008 | Lau JTF, Lau M, Cheung A, Tsui HY. A randomized controlled study to evaluate the efficacy of an Internet-based intervention in reducing HIV risk behaviors among men who have sex with men in Hong Kong. AIDS Care. 2008;20 (7):820–8. |
| 117 | Blas 2010 | Blas MM, Alva IE, Carcamo CP, Cabello R, Goodreau SM, Kimball AM, et al. Effect of an online video-based intervention to increase HIV testing in men who have sex with men in Peru. PLoS One. 2010;5(5):e10448. |
| 118 | Mustanski 2013 | Mustanski B, Garofalo R, Monahan C, Gratzer B, Andrews R. Feasibility, acceptability, and preliminary efficacy of an online HIV prevention program for diverse young men who have sex with men: the keep it up! intervention. AIDS Behav. 2013;17(9):2999–3012. |
| 119 | Kasatpibal 2014 | Kasatpibal N, Viseskul N, Srikantha W, Fongkaew W, Surapagdee N, Grimes RM. Effects of Internet-based instruction on HIV-prevention knowledge and practices among men who have sex with men. Nurs Health Sci. 2014;16(4):514–20. |
| 120 | Mustanski 2014 | Mustanski B, Ryan DT, Sanchez T, Sineath C, Macapagal K, Sullivan PS. Effects of messaging about multiple biomedical and behavioral HIV prevention methods on intentions to use among US MSM: results of an experimental messaging study. AIDS Behav. 2014;18(9):1651–60. |
| 121 | Lelutiu-Weinberger 2015 | Lelutiu-Weinberger C, Pachankis JE, Gamarel KE, Surace A, Golub SA, Parsons JT. Feasibility, acceptability, and preliminary efficacy of a live-chat social media intervention to reduce HIV risk among young men who have sex with men. AIDS Behav. 2015;19(7):1214–27. |
| 122 | Mustanski 2015 | Mustanski B, Greene GJ, Ryan D, Whitton SW. Feasibility, acceptability, and initial efficacy of an online sexual health promotion program for LGBT youth: the Queer Sex Ed intervention. J Sex Res. 2015;52(2):220–30. |
| 123 | Young SD 2015 | Young SD, Cumberland WG, Nianogo R, Menacho LA, Galea JT, Coates T. The HOPE social media intervention for global HIV prevention in Peru: a cluster randomised controlled trial. Lancet HIV. 2015;2(1):e27–32. |
| 124 | Huang 2016 | Huang E, Marlin RW, Young SD, Medline A, Klausner JD. Using Grindr, a smartphone social-networking application, to increase HIV self-testing among Black and Latino men who have sex with men in Los Angeles, 2014. AIDS Educ Prev. 2016;28(4):341–50 |
| 125 | Lau 2016 | Lau J, Lee A, Tse W, Mo P, Fong F, Wang Z, et al. A randomized control trial for evaluating efficacies of two online cognitive interventions with and without fear-appeal imagery approaches in preventing unprotected anal sex among Chinese men who have sex with men. AIDS Behav. 2016;20(9):1851–62. |
| 126 | Solorio 2016 | Solorio R, Norton-Shelpuk P, Forehand M, Monta~ no D, Stern J, Aguirre J, et al. Tu Amigo Pepe: evaluation of a multi-media marketing campaign that targets young Latino immigrant MSM with HIV testing messages. AIDS Behav. 2016;20(9):1973–88. |
| 127 | Barnabas 2016 | Barnabas RV, van Rooyen H, Tumwesigye E, et al. Uptake of antiretroviral therapy and male circumcision after communitybased HIV testing and strategies for linkage to care versus standard clinic referral: a multisite, open-label, randomised controlled trial in South Africa and Uganda. Lancet HIV. 2016;3(5):e212–20. |
| 128 | Haberer 2016 | aberer JE, Musiimenta A, Atukunda EC, et al. Short message service (SMS) reminders and real-time adherence monitoring improve antiretroviral therapy adherence in rural Uganda. AIDS (Lond Engl). 2016;30(8):1295–300. |
| 129 | Harder 2019 | Harder VS, Musau AM, Musyimi CW, Ndetei DM, Mutiso VN. A randomized clinical trial of mobile phone motivational interviewing for alcohol use problems in Kenya. Addiction. 2020;115(6):1050–60. |
| 130 | Joseph Davey 2016 | oseph Davey D, Nhavoto JA, Augusto O, et al. SMSaúde: evaluating mobile phone text reminders to improve retention in HIV care for patients on antiretroviral therapy in Mozambique. J Acquir Immune Defic Syndr (1999). 2016;73(2):e23–30. |
| 131 | Kalichman 2019 | Kalichman SC, Mathews C, Banas E, Kalichman MO. Stigma management intervention to improve antiretroviral therapy adherence: Phase-I test of concept trial, Cape Town, South Africa. Glob Public Health. 2019;14(8):1059–74. |
| 132 | Kiwanuka 2018 | Kiwanuka N, Mpendo J, Asiimwe S, et al. A randomized trial to assess retention rates using mobile phone reminders versus physical contact tracing in a potential HIV vaccine efficacy population of fishing communities around Lake Victoria, Uganda. BMC Infect Dis. 2018;18(1):591. |
| 133 | Kurth 2019 | Kurth AE, Sidle JE, Chhun N, et al. Computer-based counseling program (CARE+ Kenya) to promote prevention and HIV health for people living with HIV/AIDS: a randomized controlled trial. AIDS Educ Prev Off Publ Int Soc AIDS Educ. 2019;31(5):395–406. |
| 134 | Lapinski 2008 | apinski MK, Nwulu P. Can a short film impact HIV-related risk and stigma perceptions? Results from an experiment in Abuja, Nigeria. Health Commun. 2008;23(5):403–12. |
| 135 | Lester 2010 | Lester RT, Ritvo P, Mills EJ, et al. Effects of a mobile phone short message service on antiretroviral treatment adherence in Kenya (WelTel Kenya1): a randomised trial. Lancet. 2010;376(9755):1838–45. |
| 136 | Linnemayr 2017 | innemayr S, Huang H, Luoto J, et al. Text messaging for improv ing antiretroviral therapy adherence: no effects after 1 year in a randomized controlled trial among adolescents and young adults. Am J Public Health. 2017;107(12):1944–50. |
| 137 | MacCarthy 2020 | MacCarthy S, Wagner Z, Mendoza-Graf A, et al. A randomized controlled trial study of the acceptability, feasibility, and preliminary impact of SITA (SMS as an Incentive To Adhere): a mobile technology-based intervention informed by behavioral economics to improve ART adherence among youth in Uganda. BMC Infect Dis. 2020;20(1):173. |
| 138 | Mbuagbaw 2012 | Mbuagbaw L, Thabane L, Ongolo-Zogo P, et al. The Cameroon Mobile Phone SMS (CAMPS) Trial: a randomized trial of text messaging versus usual care for adherence to antiretroviral therapy. PLoS ONE. 2012;7(12):e46909. |
| 139 | Nsagha 2016 | Nsagha DS, Lange I, Fon PN, Nguedia Assob JC, Tanue EA. A randomized controlled trial on the usefulness of mobile text phone messages to improve the quality of care of HIV and AIDS patients in Cameroon. Open AIDS J. 2016;10:93–103. |
| 140 | Pop-Eleches 2011 | op-Eleches C, Thirumurthy H, Habyarimana JP, et al. Mobile phone technologies improve adherence to antiretroviral treatment in a resource-limited setting: a randomized controlled trial of text message reminders. AIDS (Lond Engl). 2011;25(6):825–34 |
| 141 | Reid 2017 | Reid MJA, Steenhoff AP, Thompson J, et al. Evaluation of the effect of cellular SMS reminders on consistency of antiretroviral therapy pharmacy pickups in HIV-infected adults in Botswana: a randomized controlled trial. Health Psychol Behav Med. 2017;5(1):101–9. |
| 142 | Van der Kop 2018 | Van der Kop ML, Muhula S, Nagide PI, et  al. Effect of an interactive text-messaging service on patient retention during the first year of HIV care in Kenya (WelTel Retain): an openlabel, randomised parallel-group study. Lancet Public Health. 2018;3(3):e143–52. |
| 143 | Venter 2019 | Venter WDF, Fischer A, Lalla-Edward ST, et al. Improving linkage to and retention in care in newly diagnosed HIV-positive patients using smartphones in South Africa: randomized controlled trial. JMIR Mhealth Uhealth. 2019;7(4):e12652. |
| 144 | Ybarra 2015 | Ybarra ML, Korchmaros JD, Prescott TL, Birungi R. A randomized controlled trial to increase HIV preventive information, motivation, and behavioral skills in Ugandan adolescents. Ann Behav Med Publ Soc Behav Med. 2015;49(3):473–85. |
| 145 | DiClemente 2015 | DiClemente RJ, Murray CC, Graham T, Still J. Overcoming barriers to HPV vaccination: a randomized clinical trial of a culturally-tailored, media intervention among African American girls. Hum Vaccin Immunother 2015 Sep 17;11(12):2883-2894 |
| 146 | Chen 2017 | Chen AC, Todd M, Amresh A, Menon U, Szalacha L. A pilot study of computerized, tailored intervention to promote HPV vaccination in Mexican-heritage adolescents. In: Proceedings of the 5th Annual Worldwide Nursing Conference (WNC 2017). 2017 Presented at: 5th Annual Worldwide Nursing Conference (WNC 2017); July 24-25, 2017; Singapore, Singapore |
| 147 | Pot 2017 | Pot M, Paulussen TG, Ruiter RA, Eekhout I, de Melker HE, Spoelstra ME, et al. Effectiveness of a web-based tailored intervention with virtual assistants promoting the acceptability of HPV vaccination among mothers of invited girls: randomized controlled trial. J Med Internet Res 2017 Sep 06;19(9):e312 |
| 148 | Hofstetter 2017 | Hofstetter AM, Barrett A, Camargo S, Rosenthal SL, Stockwell MS. Text messagereminders for vaccination of adolescents with chronic medical conditions: a randomized clinical trial. Vaccine 2017 Aug 16;35(35 Pt B):4554-4560 |
| 149 | Mohanty 2018 | Mohanty S, Leader AE, Gibeau E, Johnson C. Using Facebook to reach adolescents for human papillomavirus (HPV) vaccination. Vaccine 2018 Sep 25;36(40):5955-5961 |
| 150 | Ortiz 2018 | Ortiz RR, Shafer A, Cates J, Coyne-Beasley T. Development and evaluation of a social media health intervention to improve adolescents’ knowledge about and vaccination against the human papillomavirus. Glob Pediatr Health 2018 May 30;5 |
| 151 | Chen, Todd et al., 2019 | Chen AC, Kim W, Larkey L. Developing and pilot testing a digital storytelling intervention to promote HPV vaccination among Vietnamese American adolescents. GSTF J Nurs Health Care 2020 May 15:1974 |
| 152 | Chen 2019 | Chen AC, Todd M, Amresh A, Castro FG. Tailored intervention for parents to promote their adolescents’ vaccination against HPV. GSTF J Nurs Health Care 2019 May;4(1) |
| 153 | Dempsey 2019 | Dempsey AF, Maertens J, Sevick C, Jimenez-Zambrano A, Juarez-Colunga E. A randomized, controlled, pragmatic trial of an iPad-based, tailored messaging intervention to increase human papillomavirus vaccination among Latinos. Hum Vaccin Immunother 2019 Feb 20;15(7-8):1577-1584 |
| 154 | Dixon 2019 | Dixon BE, Zimet GD, Xiao S, Tu W, LindsayB, Church A, et al. An educational intervention to improve HPV vaccination: a cluster randomized trial. Pediatrics 2019 Jan 10;143(1):e20181457 |
| 155 | Panozzo 2020 | Panozzo CA, Head KJ, Kornides ML, Feemster KA, Zimet GD. Tailored messages addressing human papillomavirus vaccination concerns improves behavioral intent among mothers: a randomized controlled trial. J Adolesc Health 2020 Aug;67(2):253-261 |
| 156 | Szilagyi 2020 | Szilagyi P, Albertin C, Gurfinkel D, Beaty B, Zhou X, Vangala S, et al. Effect of state immunization information system centralized reminder and recall on HPV vaccination rates. Pediatrics 2020 May;145(5):e20192689 |
| 157 | Suzuki 2021 | Suzuki Y, Sukegawa A, Ueda Y, Sekine M, Enomoto T, Miyagi E. Effect of a brief web-based educational intervention on willingness to consider human papillomavirus vaccination for children in Japan: randomized controlled trial. J Med Internet Res 2021 Sep 27;23(9):e28355 |
| 158 | Becker 2022 | Becker ER, Shegog R, Savas LS, Frost EL, Coan SP, Healy CM, et al. Parents' experience with a mobile health intervention to influence human papillomavirus vaccination decision making: mixed methods study. JMIR Pediatr Parent 2022 Feb 21;5(1):e30340 |
| 159 | Shegog 2022 | Shegog R, Savas LS, Healy CM, Frost EL, Coan SP, Gabay EK, et al. AVPCancerFree:impact of a digital behavior change intervention on parental HPV vaccine -related perceptions and behaviors. Hum Vaccin Immunother 2022 Nov 30;18(5):2087430 |
| 160 | Chiasson 2009 | Chiasson MA, Shaw FS, Humberstone M, Hirshfield S, Hartel D. Increased HIV disclosure three months after an online video intervention for men who have sex with men (MSM). AIDS Care. 2009;21(9):1081–9. |
| 161 | Rhodes 2011 | Rhodes SD, Vissman AT, Stowers J, et al. A CBPR partner ship increases HIV testing among men who have sex with men (MSM): outcome findings from a pilot test of the CyBER/testing internet intervention. Health Educ Behav. 2011;38(3):311–20. |
| 162 | Sullivan 2017 | Sullivan PS, Driggers R, Stekler JD, et al. Usability and acceptability of a mobile comprehensive HIV prevention app for men who have sex with men: a pilot study. JMIR mHealth uHealth. 2017;5(3):e26. |
| 163 | Reback 2012 | Reback CJ, Grant DL, Fletcher JB, et al. Text messaging reduces HIV risk behaviors among methamphetamine-using men who have sex with men. AIDS Behav. 2012;16(7):1993–2002. |
| 164 | Reiter 2018 | Reiter PL, Katz ML, Bauermeister JA, Shoben AB, Paskett ED, McRee AL. Increasing human papillomavirus vaccination among young gay and bisexual men: a randomized pilot trial of the outsmart HPV intervention. LGBT Health. 2018;5(5):325–9. |
| 165 | Wray 2019 | Wray TB, Kahler CW, Simpanen EM, Operario D. A preliminary randomized controlled trial of game plan, a web application to help men who have sex with Men reduce their HIV risk and alcohol use. AIDS Behav. 2019;23(6):1668–79. |
| 166 | Bauermeister 2019 | Bauermeister JA, Tingler RC, Demers M, et al. Acceptability and preliminary efficacy of an online HIV prevention intervention for single young men who have sex with men seeking partners online: the myDEx project. AIDS Behav. 2019. |
| 167 | Starks 2019 | Starks TJ, Dellucci TV, Gupta S, et al. A pilot randomized trial of intervention components addressing drug use in couples HIV testing and counseling (CHTC) with male couples. AIDS Behav. 2019. |
| 168 | Clark 2018 | Clark JL, Segura ER, Oldenburg CE, et al. Traditional and webbased technologies to improve partner notification following syphilis diagnosis among men who have sex with men in Lima, Peru: Pilot Randomized Controlled Trial. J Med Internet Res. 2018;20(7):e232. |
| 169 | McRee 2018 | McRee AL, Shoben A, Bauermeister JA, Katz ML, Paskett ED, Reiter PL. Outsmart HPV: acceptability and short-term effects of a web-based HPV vaccination intervention for young adult gay and bisexual men. Vaccine. 2018;36(52):8158–64. |
| 170 | Adam 2011 | Adam BD, Murray J, Ross S, Oliver J, Lincoln SG, Rynard V. hivstigma.com, an innovative web-supported stigma reduction intervention for gay and bisexual men. Health Educ Res. 2011;26(5):795–807. |
| 171 | Greene 2016 | Greene GJ, Madkins K, Andrews K, Dispenza J, Mustanski B. Implementation and evaluation of the Keep It Up! Online HIV prevention intervention in a community-based setting. AIDS Educ Prev. 2016;28(3):231–45. |
| 172 | Bachmann 2013 | Bachmann LH, Grimley DM, Gao H, et al. Impact of a computerassisted, provider-delivered intervention on sexual risk behaviors in HIV-positive men who have sex with men (MSM) in a primary care setting. AIDS Educ Prev. 2013;25(2):87–101. |
| 173 | Prati 2016 | Prati G, Mazzoni D, Cicognani E, Albanesi C, Zani B. Evaluating the persuasiveness of an HIV mass communication campaign using gain-framed messages and aimed at creating a superordinate identity. Health Commun. 2016;31(9):1097–104. |
| 174 | Ko 2013 | Ko N-Y, Hsieh C-H, Wang M-C, et al. Effects of Internet popular opinion leaders (iPOL) among Internet-using men who have sex with men. J Med Internet Res. 2013;15(2):112–20. |
| 175 | Klein 2017 | Klein CH, Kuhn T, Huxley D, Kennel J, Withers E, Lomonaco CG. Preliminary findings of a technology-delivered sexual health promotion program for black men who have sex with men: quasi-experimental outcome study. JMIR Public Health Surveill. 2017;3(4):e78. |
| 176 | Mi 2015 | Mi G, Wu Z, Wang X, et al. Effects of a quasi-randomized webbased intervention on risk behaviors and treatment seeking among HIV-positive men who have sex with men in Chengdu, China. Curr HIV Res. 2017;13(6):490–6. |
| 177 | Mclver 2016 | McIver R, Dyda A, McNulty AM, Knight V, Wand HC, Guy RJ. Text message reminders do not improve hepatitis B vaccination rates in an Australian sexual health setting. J Am Med Inf Assoc (JAMIA). 2016;23(e1):e88–92. |
| 178 | Rhodes 2016 | Rhodes S, McCoy T, Tanner A, et  al. Using social media to increase HIV testing among gay and bisexual men, other men who have sex with men, and transgender persons: outcomes from a randomized community trial. Clin Infect Dis. 2016;62(11):1450–3. |
| 179 | Lin 2012 | Lin YJ, Israel T. A computer-based intervention to reduce internalized heterosexism in men. J Counsel Psychol. 2012;59(3):458–64. |
| 180 | Fleming 2017 | Fleming JB, Burns MN. Online evaluative conditioning did not alter internalized homonegativity or self-esteem in gay men. J Clin Psychol. 2017;73(9):1013–26. |
| 181 | Noslinger 2016 | Nostlinger C, Platteau T, Bogner J, et al. Computer-assisted inter vention for safer sex in HIV-positive men having sex with men: findings of a European randomized multi-center trial. J Acquir Immune Defic Syndr. 2016;71(3):e63–72. |
| 182 | Hirshfield 2012 | Hirshfield S, Chiasson MA, Joseph H, et al. An online randomized controlled trial evaluating HIV prevention digital media interventions for men who have sex with men. PLoS ONE. 2012;7(10):e46252. |
| 183 | Hightow-Weidman 2019 | Hightow-Weidman LB, LeGrand S, Muessig KE, et al. A randomized trial of an online risk reduction intervention for young black MSM. AIDS Behav. 2019;23(5):1166–77. |
| 184 | Millard 2016 | Millard T, Agius PA, McDonald K, Slavin S, Girdler S, Elliott JH. The positive outlook study: a randomised controlled trial evaluating online self-management for HIV positive gay men. AIDS Behav. 2016;20(9):1907–18. |
| 185 | Tang 2016 | Tang W, Han L, Best J, et al. Crowdsourcing HIV test promotion videos: a noninferiority randomized controlled trial in China. Clin Infect Dis. 2016;62(11):1436–42 |
| 186 | Young 2014 | Young S, Holloway I, Jaganath D, Rice E, Westmoreland D, Coates T. Project HOPE: online social network changes in an HIV prevention randomized controlled trial for African American and Latino men who have sex with men. Am J Public Health. 2014;104(9):1707–12. |
| 187 | Fernandez 2016 | Fernandez MI, Hosek SG, Hotton AL, et al. A randomized controlled trial of POWER: an Internet-based HIV prevention intervention for black bisexual men. AIDS Behav. 2016;20(9):1951–60. |
| 188 | Kerani 2011 | Kerani RP, Fleming M, DeYoung B, Golden MR. A randomized, controlled trial of inSPOT and patient-delivered partner therapy for gonorrhea and chlamydial infection among men who have sex with men. Sex Transm Dis. 2011;38(10):941–6. |
| 189 | Wang 2017 | Wang Z, Lau JTF, Ip M, et al. A randomized controlled trial evaluating efficacy of promoting a home-based HIV self-testing with online counseling on increasing hiv testing among men who have sex with men. AIDS Behav. 2017;22(1):190–201. |
| 190 | Smith 2015 | Smith KS, Hocking JS, Chen MY, et al. Dual intervention to increase chlamydia retesting: a randomized controlled trial in three populations. Am J Prev Med. 2015;49(1):1–11. |
| 191 | Cruess 2018 | Cruess DG, Burnham KE, Finitsis DJ, et al. A randomized clinical trial of a brief internet-based group intervention to reduce sexual transmission risk behavior among HIV-positive gay and bisexual men. Ann Behav Med. 2018;52(2):116–29. |
| 192 | Mustanski 2018 | Mustanski B, Parsons JT, Sullivan PS, Madkins K, Rosenberg E, Swann G. Biomedical and behavioral outcomes of keep it up! An eHealth HIV Prevention Program RCT. Am J Prev Med. 2018;55(2):151–8. |
| 193 | Abdul Rashid 2013 | Abdul Rashid RM, Mohamed M, Hamid ZA, Dahlui M. Is the phone call the most effective method for recall in cervical cancer screening?--results from a randomised control trial. Asian Pacific Journal of Cancer Prevention 2013;14(10):5901-4. |
| 194 | Belzer 2015 | Belzer ME, Kolmodin M, Clark LF, Huang J, Olson J, Kahana SY, et al. Acceptability and Feasibility of a Cell Phone Support Intervention for Youth Living with HIV with Nonadherence to Antiretroviral Therapy. AIDS Patient Care and STDs 2015;29(6):338-45. |
| 195 | Bull 2016 | Bull S, Devine S, Schmiege SJ, Pickard L, Campbell J, Shlay JC, et al. Text messaging, teen outreach program, and sexual health behavior: a cluster randomized trial. American Journal of Public Health 2016;106(S1):S117-S124. |
| 196 | Castano 2012 | Castano PM, Bynum JY, Andres R, Lara M, Westhoff C. Effect of daily text messages on oral contraceptive continuation: a randomized controlled trial. Obstetrics and Gynecology 2012;119(1):14-20. |
| 197 | Constant 2014 | Constant D, Tolly K, Harries J, Myer L. Mobile phone messages to provide support to women during the home phase of medical abortion in South Africa: a randomised controlled trial. Contraception 2014;90(3):226-33. |
| 198 | Cook 2015 | Cook PF, Carrington JM, Schmiege SJ, Starr W, Reeder B. A counselor in your pocket: feasibility of mobile health tailored messages to support HIV medication adherence. Patient Preference and Adherence 2015;9:1353-66. |
| 199 | da Costa 2012 | da Costa TM, Barbosa BJ, Gomes e Costa DA, Sigulem D, Fátima Marin H, Filho AC, et al. Results of a randomized controlled trial to assess the effects of a mobile SMS-based intervention on treatment adherence in HIV/AIDS-infected Brazilian women and impressions and satisfaction with respect to incoming messages. International Journal of Medical Informatics 2012;81(4):257-69. |
| 200 | Garofalo 2016 | Garofalo R, Kuhns LM, Hotton A, Johnson A, Muldoon A, Rice D. A randomized controlled trial of personalized text message reminders to promote medication adherence among HIV-positive adolescents and young adults. AIDS and Behavior 2016;20(5):1049-59. |
| 201 | Gerdts 2015 | Gerdts C, Moseson H, Mora M, DePineres T. Alternative follow-up options for medical abortion in Colombia: A pilot randomized controlled trial testing the feasibility of text-messages. Contraception 2015;92(4):373. |
| 202 | Hou 2010 | Hou MY, Hurwitz S, Kavanagh E, Fortin J, Goldberg AB. Using daily text-message reminders to improve adherence with oral contraceptives: A randomized controlled trial. Obstetrics and Gynecology 2010;116(3):633-40. |
| 203 | Huang 2013 | Huang D, Sangthong R, McNeil E, Chongsuvivatwong V, Zheng W, Yang X. Effects of a phone call intervention to promote adherence to antiretroviral therapy and quality of life of HIV/AIDS patients in Baoshan, China: a randomized controlled trial. AIDS Research and Treatment 2013;2013:580974. |
| 204 | Jeffries 2016 | Jeffries C, Ross P, Matoff-Stepp S, Thompson R, Harris JL, Uhrig JD, et al. Ucare4life: mobile texting to improve HIV care continuum outcomes for minority youth. In: Conference on Retroviruses and Opportunistic Infections, February 22-25, 2016, Boston, Massachussetts, United States. Vol. 24. 2016:427. |
| 205 | Lee 2016 | Lee HY, Le C, Ghebre R, Yee D. Mobile phone multimedia messaging intervention for breast cancer screening. Cancer Research 2016;76(4):Abstract P3-08-03. |
| 206 | McCarthy 2016 | McCarthy OL, French RS, Baraitser P, Roberts I, Rathod SD, Devries K, et al. Safetxt: a pilot randomised controlled trial of an intervention delivered by mobile phone to increase safer sex behaviours in young people. BMJ Open 2016;6(12):e013045. |
| 207 | Pop‐Eleches 2011 | Pop-Eleches C, Thirumurthy H, Habyarimana JP, Zivin JG, Goldstein MP, Walque D, et al. Mobile phone technologies improve adherence to antiretroviral treatment in a resource-limited setting: a randomized controlled trial of text message reminders. AIDS 2011;25(6):825-34. |
| 208 | Reed 2014 | Reed JL, Huppert JS, Taylor RG, Gillespie GL, Byczkowski TL, Kahn JA, et al. Improving sexually transmitted infection results notification via mobile phone technology. Journal of Adolescent Health 2014;55(5):690-7. |
| 209 | Ruan 2017 | Ruan Y, Xiao X, Chen J, Li X, Williams AB, Wang H. Acceptability and efficacy of interactive short message service intervention in improving HIV medication adherence in Chinese antiretroviral treatment-naive individuals. Patient Preference and Adherence 2017;11:221-8. |
| 210 | Russell 2012 | Russell SL. Effectiveness of text message reminders for improving vaccination appointment attendance and series completion among adolescents and adults. Value in Health 2012;15(4):A248. |
| 211 | Shet 2014 | Shet A, De Costa A, Kumarasamy N, Rodrigues R, Rewari BB, Ashorn P, et al. Effect of mobile telephone reminders on treatment outcome in HIV: evidence from a randomised controlled trial in India. British Medical Journal 2014;349:g5978. |
| 212 | Smith 2015 | Smith C, Ngo TD, Gold J, Edwards P, Vannak U, Sokhey L, et al. Effect of a mobile phone-based intervention on post-abortion contraception: a randomized controlled trial in Cambodia. Bulletin of the World Health Organization 2015;93(12):842-50A. |
| 213 | Bannink 2014 | Bannink R, Broeren S, Joosten-van Zwanenburg E, et al. Effectiveness of a web-based tailored intervention (E-health4Uth) and consultation to promote adolescents’ health: Randomized controlled trial. J Med Internet Res 2014; 16: e143–e143. |
| 214 | Cordova 2020 | Cordova D, Munoz-Velazquez J, Mendoza Lua F, et al. Pilot study of a multilevel mobile health app for substance use, sexual risk behaviors, and testing for sexually transmitted infections and HIV among youth: Randomized controlled trial. JMIR Mhealth Uhealth 2020; 8: e16251. Randomized Controlled Trial Research Support, N.I.H., Extramural Research Support, Non-U.S. Gov’t. |
| 215 | McCarthy 2020 | McCarthy OL, Aliaga C, Torrico Palacios ME, et al. An intervention delivered by mobile phone instant messaging to increase acceptability and use of effective contraception among young women in Bolivia: Randomized controlled trial. J Med Internet Res 2020; 22: e14073. |
| 216 | McCarthy 2019 | McCarthy OL, Zghayyer H, Stavridis A, et al. A randomized controlled trial of an intervention delivered by mobile phone text message to increase the acceptability of effective contraception among young women in Palestine. Trials 2019; 20: 228. doi:10.1186/s13063-019-3297-4. |
| 217 | Saberi 2021 | Saberi P, McCuistian C, Agnew E, et al. Video-counseling intervention to address HIV care engagement, mental health, and substance use challenges: A pilot randomized clinical trial for youth and young adults living with HIV. Telemed Rep 2021; 2: 14–25. |
| 218 | Scull TM 2018 | Scull TM, Kupersmidt JB, Malik CV, et al. Examining the efficacy of an mHealth media literacy education program for sexual health promotion in older adolescents attending community college. J Am Coll Health 2018; 66: 165–177. |
| 219 | Starosta 2016 | Starosta AJ, Cranston E and Earleywine M. Safer sex in a digital world: a web-based motivational enhancement intervention to increase condom use among college women. J Am Coll Health 2016; 64: 184–193. |
| 220 | Stephenson 2020 | Stephenson R, Todd K, Kahle E, et al. Project moxie: results of a feasibility study of a telehealth intervention to increase HIV testing among binary and nonbinary transgender youth. AIDS Behav 2020; 24: 1517–1530 |
| 221 | Widman 2018 | Widman L, Golin CE, Kamke K, et al. Sexual assertiveness skills and sexual decision-making in adolescent girls: Randomized controlled trial of an online program. Am J Public Health 2018; 108: 96–102 |
| 222 | Wong 2021 | Wong JY, Zhang W, Wu Y, et al. An Interactive Web-Based Sexual Health Literacy Program for Safe Sex Practice for Female Chinese University Students: Multicenter Randomized Controlled Trial. J Med Internet Res 2021; 23: e22564. 2021/03/13. doi:10.2196/22564. |
| 223 | Belzer 2014 | Belzer, M. E., Naar-King, S., Olson, J., Sarr, M., Thornton, S., Kahana, S. Y. et al. .. (2014) The use of cell phone support for non-adherent HIV-infected youth and young adults: An initial randomized and controlled intervention trial. AIDS and Behavior, 18, 686–696, doi |
| 224 | Bull 2017 | Bull, S., Devine, S., Schmiege, S. J., Hammes, A., Pickard, L. and Shlay, J. C. (2017) Text messaging and teen sexual health behavior: Long-term follow-up of a cluster-randomized trial. CIN: Computers, Informatics. Nursing, 35, 549–553, doi:10.1097/CIN.0000000000000404. |
| 225 | Hacking 2019 | Hacking, D., Mgengwana-Mbakaza, Z., Cassidy, T., Runeyi, P., Duran, L. T., Mathys, R. H. et al. . (2019) Peer mentorship via mobile phones for newly diagnosed HIV-positive youths in-clinic care in Khayelitsha, South Africa: Mixed methods study. Journal of Medical Internet Research, 21, e14012, doi:10.2196/14012. |
| 226 | John 2016 | John, M. E., Samson-Akpan, P. E., Etowa, J. B., Akpabio, I. I. and John, E. E. (2016) Enhancing self-care, adjustment, and engagement through mobile phones in youth with HIV. International Nursing Review, 63, 555–561, doi:10.1111/inr.12313. |
| 227 | Merril 2018 | Merrill, K. G., Merrill, J. C., Hershow, R. B., Barkley, C., Rakosa, B., DeCellesa, J. et al. . (2018) Linking at-risk South African girls to sexual violence and reproductive health services: A mixed-methods assessment of a soccer-based HIV prevention program and pilot SMS campaign. Evaluation and Program Planning, 70, 12–24, doi:10.1016/j.evalprogplan.2018.04.010. |
| 228 | Rockicki 2017 | Rokicki, S. P., Cohen, J. P., Salomon, J. A. P. and Fink, G. P. (2017) Impact of a text-messaging program on adolescent reproductive health: A cluster-randomized trial in Ghana. American Journal of Public Health, 107, 298–305, doi:10.2105/AJPH.2016.303562M. |
| 229 | Stankievich 2018 | Stankievich, E., Malanca, A., Foradori, I., Ivalo, S. and Losso, M. (2018) The utility of mobile communication devices as a tool to improve adherence to antiretroviral treatment in HIV-infected children and young adults in Argentina. Pediatric Infectious Disease Journal, 37, 345–348, doi:10.1097/inf.0000000000001807. |
| 230 | Yao 2018 | Yao, P., Fu, R., Craig Rushing, S., Stephens, D., Ash, J. S. and Eden, K. B. (2018) Texting 4 sexual health: Improving attitudes, intention, and behavior among American Indian and Alaska Native youth. Health Promotion Practice, 19, 833–843, doi:10.1177/1524839918761872. |
| 231 | Ybarra 2018 | Ybarra, M. L., Liu, W., Prescott, T. L., Phillips, G., 2nd and Mustanski, B. (2018) The effect of a text messaging-based HIV prevention program on sexual minority male youths: A national evaluation of information, motivation, and behavioral skills in a randomized controlled trial of Guy2Guy. AIDS and Behavior, 22, 3335–3344, doi:10.1007/s10461-018-2118-1. |
| 232 | Brayboy 2017 | Brayboy, L. M., Sepolen, A., Mezoian, T., Schultz, L., Landgren-Mills, B. S., Spencer, N. et al. . (2017) Girl talk: A smartphone application to teach sexual health education to adolescent girls. Journal of Pediatric and Adolescent Gynecology, 30, 23–28, doi:10.1016/j.jpag.2016.06.011. |
| 233 | Dehghani 2016 | Dehghani, E., Erfanian, F., Khadivzadeh, T. and Shakeri, M. T. (2019) The impact of a high-risk sexual behaviour prevention program via mobile application on sexual knowledge and attitude of female students. Journal of Midwifery & Reproductive Health, 7, 1491–1498. doi:10.22038/jmrh.2018.21382.1224. |
| 234 | Jeong 2017 | Jeong, S., Cha, C. and Lee, J. (2017) The effects of STI education on Korean adolescents using smartphone applications. Health Education Journal, 76, 775–786, doi:10.1177/0017896917714288. |
| 235 | Manlove 2020 | Manlove, J., Cook, E., Whitfield, B., Johnson, M., Martinez-Garcia, G. and Garrido, M. (2020) Short-term impacts of pulse: An app-based teen pregnancy prevention program for black and Latinx women. Journal of Adolescent Health, 66, 224–232, doi:10.1016/j.jadohealth.2019.08.017. |
| 236 | Mesheriakova 2017 | Mesheriakova, V. V. and Tebb, K. P. (2017) Effect of an iPad-based intervention to improve sexual health knowledge and intentions for contraceptive use among adolescent females at school-based health centers. Clinical Pediatrics, 56, 1227–1234, doi:10.1177/0009922816681135. |
| 237 | Nielsen 2019 | Nielsen, A. M., De Costa, A., Gemzell-Danielsson, K., Marrone, G., Boman, J., Salazar, M. et al. . (2019) The MOSEXY trial: Mobile phone intervention for sexual health in youth – A pragmatic randomised controlled trial to evaluate the effect of a smartphone application on sexual health in youth in Stockholm, Sweden. Sexually Transmitted Infections, 0, 1–6, doi:10.1136/sextrans-2019-054027. |
| 238 | Bailey 2013 | Bailey, J. V., Pavlou, M., Copas, A., McCarthy, O., Carswell, K., Rait, G. et al. . (2013) The Sexunzipped trial: Optimizing the design of online randomized controlled trials. Journal of Medical Internet Research, 15, e278. |
| 239 | Ballester-Arnal 2015 | Ballester-Arnal, R., Gil-Llario, M. D., Giménez-García, C. and Kalichman, S. C. (2015) What works well in HIV prevention among Spanish young people? An analysis of differential effectiveness among six intervention techniques. AIDS and Behavior, 19, 1157–1169. |
| 240 | Brown 2016 | Brown, K. E., Newby, K., Caley, M., Danahay, A. and Kehal, I. (2016) Pilot evaluation of a web-based intervention targeting sexual health service access. Health Education Research, 31, 273–82, doi:10.1093/her/cyw003. |
| 241 | Doubova 2017 | Doubova, S. V., Martinez-Vega, I. P., Infante-Casta°eda, C. and P°rez-Cuevas, R. (2017) Effects of an internet-based educational intervention to prevent high-risk sexual behavior in Mexican adolescents. Health Education Research, 32, 487–498, doi:10.1093/her/cyx074. |
| 242 | Gottvall 2010 | Gottvall Rn, M., Tydén, T., Höglund, A. T. and Larsson, M. (2010) Knowledge of human papillomavirus among high school students can be increased by an educational intervention. International Journal of STD and AIDS, 21, 558–562, doi:10.1258/ijsa.2010.010063. |
| 243 | Horvath 2017 | Horvath, K. J. and Bauermeister, J. A. (2017) Ehealth literacy and intervention tailoring impacts the acceptability of a HIV STI testing intervention and sexual decision making among young gay and bisexual men. AIDS Education and Prevention, 29, 14–23. |
| 244 | Lustria 2016 | Lustria, M. L. A., Cortese, J., Gerend, M. A., Schmitt, K., Kung, Y. M. and McLaughlin, C. (2016) A model of tailoring effects: A randomized controlled trial examining the mechanisms of tailoring in a web-based std screening intervention. Health Psychology, 35, 1214–1224, doi:10.1037/hea0000399. |
| 245 | Massey 2013 | Massey, P., Prelip, M., Rideau, A. and Glik, D. C. (2013) School-based HIV prevention in Dakar, Senegal: Findings from a peer-led program. International Quarterly in Community Health Education, 33, 129–141. |
| 246 | Mevissen 2011 | Mevissen, F. E., Ruiter, R. A., Meertens, R. M., Zimbile, F. and Schaalma, H. P. (2011) Justify your love: Testing an online STI-risk communication intervention designed to promote condom use and STI-testing. Psychology & Health, 26, 205–221, doi:10.1080/08870446.2011.531575. |
| 247 | Mortimer 2015 | Mortimer, N. J., Rhee, J., Guy, R., Hayen, A. and Lau, A. Y. S. (2015) A web-based personally controlled health management system increases sexually transmitted infection screening rates in young people: A randomized controlled trial. Journal of the American Medical Informatics Association, 22, 805–814, doi:10.1093/jamia/ocu052. |
| 248 | Naar-King 2013 | Naar-King, S., Outlaw, A. Y., Sarr, M., Parsons, J. T., Belzer, M., MacDonell, K. et al. . (2013) Motivational Enhancement System for Adherence (MESA): Pilot randomized trial of a brief computer-delivered prevention intervention for youth initiating antiretroviral treatment. Journal of Pediatric Psychology, 38, 638–648, doi:10.1093/jpepsy/jss132. |
| 249 | Spielberg 2014 | Spielberg, F., Levy, V., Lensing, S., Chattopadhyay, I., Venkatasubramanian, L., Acevedo, N. et al. . (2014) Fully integrated e-services for prevention, diagnosis, and treatment of sexually transmitted infections: Results of a 4-county study in California. American Journal of Public Health, 104, 2313–20, doi:10.2105/AJPH.2014.302302. |
| 250 | Starling 2014 | Starling, R., Helme, D., Nodulman, J. A., Bryan, A. D., Buller, D. B., Donohew, R. L. et al. . (2014) Testing a risky sex behavior intervention pilot website for adolescents. Californian Journal of Health Promotion, 12, 24–34, doi:10.32398/cjhp.v12i3.1578. |
| 251 | Villegas 2015 | Villegas, N., Santisteban, D., Cianelli, R., Ferrer, L., Ambrosia, T., Peragallo, N. et al. . (2015) Pilot testing an internet-based STI and HIV prevention intervention with Chilean women. Journal of Nursing Scholarship, 47, 106–116, doi:10.1111/jnu.12114. |
| 252 | Carvalho 2016 | Carvalho, T., Alvarez, M., Pereira, C. and Schwarzer, R. (2016) Stage-based computer-delivered interventions to increase condom use in young men. International Journal of Sexual Health, 28, 176–186, doi:10.1080/19317611.2016.1158764. |
| 253 | Castillo-Arcos 2016 | Castillo-Arcos Ldel, C., Benavides-Torres, R. A., Lopez-Rosales, F., Onofre-Rodriguez, D. J., Valdez-Montero, C. and Maas-Gongora, L. (2016) The effect of an internet-based intervention designed to reduce HIV/aids sexual risk among Mexican adolescents. AIDS Care, 28, 191–196, doi:10.1080/09540121.2015.1073663. |
| 254 | Chong 2020 | Chong, A., Gonzalez-Navarro, M., Karlan, D. and Valdivia, M. (2020) Do information technologies improve teenagers’ sexual education? Evidence from a randomized evaluation in Colombia. The World Bank Economic Review, 34, 371–392, doi:10.1093/wber/lhy031. |
| 255 | Kaufman 2018 | Kaufman, C. E., Schwinn, T. M., Black, K., Keane, E. M., Big Crow, C. K., Shangreau, C. et al. . (2018) Impacting precursors to sexual behavior among young American Indian adolescents of the northern plains: A cluster randomized controlled trial. Journal of Early Adolescence, 38, 988–1007, doi:10.1177/0272431617708055. |
| 256 | Klein 2017 | Klein, C. H., Kuhn, T., Altamirano, M. and Lomonaco, C. (2017) C-safe: A computer-delivered sexual health promotion program for Latinas. Health Promotion Practice, 18, 516–525, doi:10.11771524839917707791. |
| 257 | Shafii 2019 | Shafii, T., Benson, S. K., Morrison, D. M., Hughes, J. P., Golden, M. R. and Holmes, K. K. (2019) Results from e-KISS: Electronic-KIOSK Intervention for Safer Sex: A pilot randomized controlled trial of an interactive computer-based intervention for sexual health in adolescents and young adults. PLoS One, 14, e0209064, doi:10.1371/journal.pone.0209064. |
| 258 | Markham 2020 | Markham, C. M., Peskin, M. F., Baumler, E. R., Addy, R. C., Thiel, M. A., Laris, B. A. et al. . (2020) Socio-ecological factors associated with students’ perceived impact of an evidence-based sexual health education curriculum. Journal of School Health, 90, 604–617, doi:10.1111/josh.12908. |
| 259 | Dulli 2020 | Dulli, L., Ridgeway, K., Packer, C., Murray, K. R., Mumuni, T., Plourde, K. F. et al. . (2020) A social media-based support group for youth living with HIV in Nigeria (smart connections): Randomized controlled trial. Journal of Medical Internet Research, 22, e18343, doi:10.2196/18343. |
| 260 | Whiteley 2018 | Whiteley, L., Brown, L. K., Mena, L., Craker, L. and Arnold, T. (2018) Enhancing health among youth living with HIV using an iPhone game. AIDS Care, 30, 21–33, doi:10.1080/09540121.2018.1503224. |
| 261 | Ezegbe 2018 | Ezegbe B, Eseadi C, Ede MO, Igbo JN, Aneke A, Mezieobi D, Ugwu GC, Ugwoezuonu AU, Elizabeth E, Ede KR, Ede AO, Ifelunni CO, Amoke C, Eneogu ND, Effanga OA. Efficacy of rational emotive digital storytelling intervention on knowledge and risk perception of HIV/AIDS among schoolchildren in Nigeria. Medicine (Baltimore). 2018 Nov;97(47):e12910. doi: 10.1097/MD.0000000000012910. Erratum in: Medicine (Baltimore). 2018 Dec;97(51):e13848. doi: 10.1097/MD.0000000000013848. PMID: 30461604; PMCID: PMC6393154. |
| 262 | Sznitman 2010 | Sznitman, S., Vanable, P. A., Carey, M. P., Hennessy, M., Brown, L. K., Valois, R. F. et al. . (2011) Using culturally sensitive media messages to reduce HIV-associated sexual behavior in high-risk African American adolescents: Results from a randomized trial. Journal of Adolescent Health, 49, 244–251, doi:10.1016/j.jadohealth.2010.12.007. |
| 263 | Blas 2014 | Blas M, Menacho L, Alva I. Randomized Controlled Trial to Evaluate the Effect of a Novel Web-based Intervention to Increase HIV Testing in Men Who Have Sex With Men in Lima-Peru. ICH GCP: Good Clinical Practice. 2014. |
| 264 | Patel 2016 | Patel V, Rawat S, Dange A, Lelutiu-Weinberger C, Golub SA. An internet-based, peer-delivered messaging intervention for hiv testing and condom use among men who have sex with men in India (CHALO!): pilot randomized comparative trial. JMIR Public Health Surveill 2020 Apr 16;6(2):e16494 |
| 265 | Washington 2017 | Washington TA, Applewhite S, Glenn W. Using Facebook as a platform to direct young black men who have sex with men to a video-based HIV testing intervention: a feasibility study. Urban Soc Work 2017;1(1):36-52 |
| 266 | Anand 2018 | Anand T, Nitpolprasert C, Jantarapakde J, Meksena R, Phomthong S, Phoseeta P, Phanuphak P, Phanuphak N. Implementation and impact of a technology-based HIV risk-reduction intervention among Thai men who have sex with men using 'Vialogues': a randomized controlled trial. AIDS Care. 2020 Mar;32(3):394–405. doi: 10.1080/09540121.2019.1622638. |
| 267 | Christensen 2007 | Christensen JL. CORE – Aggregating the world's open access research papers. Ann Arbor: University of Southern California; 2007. [2018-09-01]. When It's Good to Feel Bad: How Responses to Virtual Environments Predict Real-Life Sexual Risk-reduction |
| 268 | Desai 2014 | Desai M, Nardone A, Burns F, Mercey D, Gilson R, Muniina P, Sharp T, Wayal S. Active recall of men who have sex with men (MSM) for an HIV/STI testing: a feasible and effective strategy?. Abstracts of the Third Joint Conference of the British HIV Association (BHIVA) with the British Association for Sexual Health and HIV (BASHH); Third Joint Conference of the BHIVA and the BASHH; April 1-4, 2014; Liverpool. 2014. p. 109. |
| 269 | Habarta 2017 | Habarta N, Boudewyns V, Badal H, Johnston J, Uhrig J, Green D, Ruddle P, Rosenthal J, Stryker JE. CDC'S Testing Makes Us Stronger (TMUS) Campaign: Was Campaign Exposure Associated With HIV Testing Behavior Among Black Gay and Bisexual Men? AIDS Educ Prev. 2017 Jun;29(3):228–40. doi: 10.1521/aeap.2017.29.3.228. |
| 270 | Hilliam 2011 | Hilliam A, Fraser L, Turner L. NHS Health Scotland. Scotland: NHS Health Scotland; 2011. Feb, [2018-09-01]. HIV Wake-Up Campaign Evaluation http://www.healthscotland.com/uploads/documents/15963-HIV%20Wake%20Up%20Campaign%20Evaluation.pdf. [Google Scholar] |
| 271 | Lelutiu-Weinberger 2018 | Leluțiu-Weinberger C, Manu M, Ionescu F, Dogaru B, Kovacs T, Dorobănțescu C, Predescu M, Surace A, Pachankis JE. An mHealth intervention to improve young gay and bisexual men's sexual, behavioral, and mental health in a structurally stigmatizing national context. JMIR Mhealth Uhealth. 2018 Nov 14;6(11):e183. doi: 10.2196/mhealth.9283. |
| 272 | Mikolajczak 2013 | Mikolajczak J, Breukelen van G, Kok G, Hospers H. Evaluation of an online HIV-prevention intervention to promote HIV-testing among men who have sex with men: a randomised controlled trial. Neth J Psychol. 2012;67(2):21–35. |
| 273 | Nöstlinger 2016 | Nöstlinger C, Platteau T, Bogner J, Buyze J, Dec-Pietrowska J, Dias S, Newbury-Helps J, Kocsis A, Mueller M, Rojas D, Stanekova D, van Lankveld J, Colebunders R, Eurosupport Study Group Implementation and operational research: computer-assisted intervention for safer sex in HIV-positive men having sex with men: findings of a European randomized multi-center trial. J Acquir Immune Defic Syndr. 2016 Mar 1;71(3):e63–72. doi: 10.1097/QAI.0000000000000882 |
| 274 | Uhrig 2012 | Uhrig JD, Lewis MA, Bann CM, Harris JL, Furberg RD, Coomes CM, Kuhns LM. Addressing HIV knowledge, risk reduction, social support, and patient involvement using SMS: results of a proof-of-concept study. J Health Commun. 2012;17(Suppl 1):128–45. doi: 10.1080/10810730.2011.649156. |
| 275 | Buchbinder 2023 | Buchbinder SP, Siegler AJ, Coleman K, et al. Randomized controlled trial of automated directly observed therapy for measurement and support of PrEP adherence among young men who have sex with men. AIDS Behav. 2023;27(2):719–732. |
| 276 | Erenrich 2024 | Erenrich RK, Braun RA, Torres-Mendoza DM, et al. Effectiveness of PrEPTECH: findings from a 180-day randomized controlled trial of a pre-exposure prophylaxis telehealth intervention. J Acquir Immune Defic Syndr. 2024;95(5):463–469. |
| 277 | Liu 2021 | Liu Z, Bao R, Zhang X, et al. A brief smartphone-based intervention significantly improved pre-exposure prophylaxis adherence among Chinese men who have sex with men: findings of a randomized controlled trial. J Infect. 2022;84(2):248–288. |
| 278 | Schnall 2022 | Schnall R, Kuhns LM, Pearson C, et al. Efficacy of MyPEEPS Mobile, an HIV prevention intervention using mobile technology, on reducing sexual risk among same-sex attracted adolescent males: a randomized clinical trial. JAMA Netw Open. 2022;5(9):e2231853. |
| 279 | Schneider 2021 | Schneider JA, Young L, Ramachandran A, et al. A pragmatic randomized controlled trial to increase PrEP uptake for HIV prevention: 55-week results from PrEPChicago. J Acquir Immune Defic Syndr. 2021;86(1):31–37. |
| 280 | Serrano 2023 | Serrano VB, Moore DJ, Morris S, et al. Efficacy of daily text messaging to support adherence to HIV pre-exposure prophylaxis (PrEP) among stimulant-using men who have sex with men. Subst Use Misuse. 2023;58(3):465–469. |
| 281 | Sullivan 2022 | Sullivan PS, Stephenson R, Hirshfield S, et al. Behavioral efficacy of a sexual health mobile app for men who have sex with men: randomized controlled trial of Mobile Messaging for Men (M-cubed). J Med Internet Res. 2022;24(2):e34574. |
| 282 | Biello 2022 | Biello KB, Daddario SR, Hill-Rorie J, et al. Uptake and acceptability of MyChoices: results of a pilot RCT of a mobile app designed to increase HIV testing and PrEP uptake among young American MSM. AIDS Behav. 2022;26(12):3981–3990. |
| 283 | Li 2023 | Li C, Xiong Y, Maman S, et al. An instant messaging mobile phone application for promoting HIV pre-exposure prophylaxis uptake among Chinese gay, bisexual and other men who have sex with men: a mixed methods feasibility and piloting randomized controlled trial study. PLoS One. 2023;18(11):e0285036. |
| 284 | Wray 2024 | Wray TB, Chan PA, Kahler CW, et al. Pilot randomized controlled trial of Game Plan for PrEP: a brief, web and text message intervention to help sexual minority men adhere to PrEP and reduce their alcohol use. AIDS Behav. 2024;28(4):1356–1369. |
| 285 | Cheng 2019 | Cheng W, Xu H, Tang W, Zhong F, Meng G, Han Z, et al. Online HIV prevention intervention on condomless sex among men who have sex with men: a web-based randomized controlled trial. BMC Infect Dis. 2019;19(1):644. |
| 286 | Chiou 2020 | Chiou PY, Liao PH, Liu CY, Hsu YT. Effects of mobile health on HIV risk reduction for men who have sex with men. AIDS Care. 2020;32(3):316–324. |
| 287 | Yun 2021 | Yun K, Chu Z, Zhang J, Geng W, Jiang Y, Dong W, et al. Mobile phone intervention based on an HIV risk prediction tool for HIV prevention among men who have sex with men in China: randomized controlled trial. JMIR Mhealth Uhealth. 2021;9(4):e19511. |
| 288 | Li 2020 | Li XX, Zhao J, Liu Q, Xie XQ, Liu HW, Long QP, et al. A comparative study on the effects of “Internet+” AIDS network intervention and field intervention for men in contact groups. Systems Medicine. 2020;5(12):39–41. |
| 289 | Xiao 2020 | Xiao ST, Chen P, Zhu LD, Jin YZ, Xin X. Effect evaluation of precise AIDS intervention on MSM by using WeChat official account. Chin J AIDS STD. 2020;26(6):611–614. |
| 290 | Luo 2023 | Luo Q, Wu Z, Mi G, Xu J, Scott SR. Using HIV risk self-assessment tools to increase HIV testing in men who have sex with men in Beijing, China: app-based randomized controlled trial. J Med Internet Res. 2023;25:e45262. |
| 291 | Tang 2019 | Tang W, Mao J, Liu C, Mollan K, Zhang Y, Tang S, et al. Reimagining health communication: a noninferiority randomized controlled trial of crowdsourced intervention in China. Sex Transm Dis. 2019;46(3):172–178. |
| 292 | Liu 2014 | Liu LQ, Li P, Li J. Analysis on effects of the AIDS infections intervention for MSM using internet. Chin J Dis Control Prev. 2014;18(12):1232–1234. |
| 293 | Song 2017 | Song LP, Tang J, Zhang YL, Zhang ZK, Lan GH, Liu YL. Impact of text message intervention on HIV-related high risk sexual behavior of MSM. Chin J Dis Control Prev. 2017;23(10):932–934. |
| 294 | Tao 2020 | Tao JH, Fang YR, Lu QL, Yang ZK. Effect evaluation of follow-up intervention among internet-based self-testing for men having sex with men in Shaoxing. Chin J AIDS STD. 2020;26(9):958–961. |
| 295 | Yan 2013 | Yan HM, Gao C, Li Y, Tong X, Hui S, Yu L. Evaluation of QQ-based HIV high-risk behavior interventions for MSM. Chin J AIDS STD. 2013;(3):174–176. |
| 296 | Zhang 2014 | Zhang TL, Liu Z, Sun SY. Evaluation of the effectiveness of network intervention on HIV/AIDS prevention and control among gay men. Prev Med Trib. 2014;20(8):609–610. |
| 297 | Liu 2012 | Liu GW, Lu HY, Wang J, Cao XB. Evaluation of web-based HIV/AIDS interventions among men who have sex with men. Chin J AIDS STD. 2012;18(9):578–580. |
| 298 | Wang FX 2011 | Wang FX, Huang YL. Analysis on effect of AIDS related high risk behavior intervention among MSM in Yingtan city. Prev Med Trib. 2011;17(6):518–519. |
| 299 | Wang XD 2014 | Wang XD, Yang D, Ma X, Fan G, Xu H, Lin H, et al. An initial explore of multidimensional and full coverage intervention mode for MSM. Pract J Clin Med. 2014;11(3):62–64. |
| 300 | Wang Y 2009 | Wang Y, Zhang HB, Li ZJ, Xu J, Zhang GG, Dou Z, et al. Analysis of AIDS prevention services for MSM group and promotion effectiveness of standardize treatment for STD. Occup Health. 2009;25(15):1586–1588. |
| 301 | Xie 2018 | Xie YL, Chen BF, Zhang QL, Pan YJ, Luo HJ, Zhuo BG, et al. Evaluation of internet-based HIV/AIDS interventions among men who have sex with men in Dongguan. J Prev Med. 2018;34(3):273–277. |
| 302 | Moore 2018 | Moore DJ, Jain S, Dubé MP, Daar ES, Sun X, Young J, et al. Randomized controlled trial of daily text messages to support adherence to pre-exposure prophylaxis in individuals at risk for human immunodeficiency virus: The TAPIR Study. Clinical Infectious Diseases. 2018;66(10):1566-1572. doi:10.1093/cid/cix1055. |
| 303 | Bauermeister 2019 | Bauermeister JA, Tingler RC, Demers M, Connochie D, Gillard G, Shaver J, et al. Acceptability and preliminary efficacy of an online HIV prevention intervention for single young men who have sex with men seeking partners online: The myDEx project. AIDS and Behavior. 2019;23(11):3064-3077. doi:10.1007/s10461-019-02426-7. |
| 304 | Njuguna 2019 | Njuguna N, Ngure K, Mugo N, Wamoni E, Casmir E, Beima-Sofie K, et al. The Effect of text-message–based HIV prevention on HIV testing among young women in Kenya: A randomized controlled trial. Sexually Transmitted Diseases. 2019;46(6):353-359. doi:10.1097/OLQ.0000000000000987. |
| 305 | Songtaweesin 2020 | Songtaweesin WN, Bermudez LG, Jantarapakde J, Pengnonyang S, Kawichai S, Trachunthong D, et al. Youth-friendly services and a mobile phone application to promote adherence to pre-exposure prophylaxis among adolescent men who have sex with men and transgender women at risk for HIV in Thailand: A randomized controlled trial. Journal of the International AIDS Society. 2020;23(5):e25564. doi:10.1002/jia2.25564. |
| 306 | Haberer 2021 | Haberer JE, Patel R, Baeten JM, Bekker L-G, Bukusi E, Donnell D, et al. SMS reminders for adherence in young Kenyan women receiving pre-exposure prophylaxis for HIV prevention (MPYA Study): A randomized controlled trial. The Lancet HIV. 2021;8(3):e130-e137. doi:10.1016/S2352-3018(20)30315-0. |
| 307 | Lin 2023 | Lin Y, Zhang W, Tang W, Li H, Chen W, Fu R, et al. Effectiveness of a digital, crowdsourced, multilevel intervention on HIV testing among men who have sex with men in China: Randomized controlled trial. JMIR (Journal of Medical Internet Research). 2023;25:e46890. doi:10.2196/46890. |
| 308 | Wray 2022 | Wray TB, Chan PA, Kahler CW, Simpanen EM, Suffoletto B, Fortenberry JD, et al. Pilot randomized controlled trial of Game Plan: A web-based intervention with text-messaging to increase HIV pre-exposure prophylaxis uptake and reduce sexual risk among sexual minority men. JMIR Formative Research. 2022;6(4):e30408. doi:10.2196/30408. |
| 309 | Horvath 2024 | Horvath KJ, Noor SW, Erickson D, Lammert S, Stojanovski K, Brown W, et al. A smartphone-based program (PrEP iT) to improve adherence to HIV pre-exposure prophylaxis among men who have sex with men: Randomized controlled trial. AIDS and Behavior. 2024;28(10):2804-2820. doi:10.1007/s10461-023-04233-1. |
| 310 | Biello 2025 | Biello KB, Hill-Rorie J, Valente P, Marrow E, Mayer KH, Mimiaga MJ, et al. Effectiveness of the MyChoices and LYNX mobile applications to promote HIV testing and pre-exposure prophylaxis among young men who have sex with men: A randomized controlled trial. JMIR Public Health and Surveillance. 2025;11:e63428. doi:10.2196/63428. |
| 311 | Brody 2022 | Brody C, Chhoun P, Tuot S, Fehrenbacher AE, Moran A, Swendeman D, et al. A mobile intervention to link young female entertainment workers in Cambodia to health and gender-based violence services: randomized controlled trial. J Med Internet Res. 2022;24(1):e27696. doi:10.2196/27696. |
| 312 | Levy 2021 | Levy E, Warner LM, Fleig L, Kaufman MR, Deschepper R, Gidron Y. The effects of psychological inoculation on condom use tendencies and barriers: a randomized controlled trial. Psychol Health. 2021;36(5):575–592. doi:10.1080/08870446.2020.1775832. |
| 313 | Logie 2023 | Logie CH, Okumu M, Berry I, Hakiza R, Baral SD, Musoke DK, et al. Findings from the Tushirikiane mobile health (mHealth) HIV self-testing pragmatic trial with refugee adolescents and youth living in informal settlements in Kampala, Uganda. J Int AIDS Soc. 2023;26(10):e26185. doi:10.1002/jia2.26185. |
| 314 | McCrimmon 2024 | McCrimmon J, Widman L, Javidi H, Bransletter J, Hurst J, Lavery J, et al. Evaluation of a brief online sexual health program for adolescents: randomized controlled trial. Health Promot Pract. 2024;25(4):? (paginación en el pdf: 649–663 aprox.) doi:10.1177/15248399231126379. |
| 315 | Nelson 2022 | Nelson KM, Perry NS, Stout CD, Dunsiger SI, Carey MP. The Young Men and Media study: a pilot randomized controlled trial of a community-informed, online HIV prevention intervention for 14-17-year-old sexual minority males. AIDS Behav. 2022;26(2):569–583. doi:10.1007/s10461-021-03412-8. |
| 316 | Newcomb 2022 | Newcomb ME, Swann G, Macapagal K, Sarno EL, Whitton SW, Mustanski B. Biomedical and behavioral outcomes of 2GETHER: a randomized controlled trial of a telehealth HIV prevention program for young male couples. J Consult Clin Psychol. 2023;91(9):505–520. doi:10.1037/ccp0000823. |
| 317 | Santa Maria 2021 | Santa Maria D, Padhye N, Bushelle M, Yang Y, Jones S, Jims A, et al. Efficacy of a just-in-time adaptive intervention to promote HIV risk reduction behaviors among young adults experiencing homelessness: pilot randomized controlled trial. J Med Internet Res. 2021;23(7):e27674. doi:10.2196/27674. |
| 318 | Swendeman 2024 | Swendeman D, Rotheram-Borus MJ, Arnold E, Fernandez M, Comulada W, Lee SJ, et al. Optimal strategies to improve uptake and adherence to HIV prevention among young people: results of the ATN 149 randomized factorial trial in the USA. Lancet Digit Health. 2024;6(3):e187–e200. doi:10.1016/S2589-7500(23)00205-2. |
| 319 | Widman 2020 | Widman L, Kamke K, Evans R, Fernandez MJ, Comulada WS, Lee SJ, et al. Feasibility, acceptability, and preliminary efficacy of a brief online sexual health program for adolescents. J Sex Res. 2020;57(2):145–154. doi:10.1080/00224499.2019.1630801. |
| 320 | Yi 2024 | Yi M, Li X, Chiaramonte D, Sun S, Pan S, Soulliard Z, et al. Guided internet-based LGBTQ-affirmative cognitive-behavioral therapy: a randomized controlled trial among sexual minority men in China. Behav Res Ther. 2024;181:104605. doi:10.1016/j.brat.2024.104605. |
| 321 | Zhang 2024 | Zhang K, Cao B, Fang Y, Liang X, Ye D, Chen YQ, et al. Comparing the efficacy of 2 WeChat mini programs in reducing nonmarital heterosexual contact by male factory workers: randomized controlled trial. J Med Internet Res. 2024;26:e49362. |

Section 5 – Search strategy in line-by-line format

The following search strategy was developed and reported according to the PRISMA-S (Preferred Reporting Items for Systematic Reviews and Meta-Analyses Literature Search Extension) checklist to ensure transparency and reproducibility.

The strategy was initially executed on August 31, 2024, and rerun on November 12, 2025, without modifications to databases or syntax. The full search was designed and peer-reviewed by an experienced librarian.

| **Term** | **#** | **Boolean** |
| --- | --- | --- |
| *Sexually transmitted infections* | 1 | (sexual* AND transmit* ) OR "sexually-transmitted" |
|  | 2 | infection* OR disease |
|  | 3 | #1 AND #2 |
|  | 4 | stis OR stds OR sti OR std OR venereal* |
|  | **5** | **#3 OR #4** |
| *Pelvic Inflammatory Disease (PID)* | 6 | pelvic* AND inflammatory* |
|  | 7 | upper* AND genital* AND tract |
|  | 8 | #2 AND #7 |
|  | 9 | PID |
|  | **10** | **#6 OR #8 OR #9** |
| *Gonorrhea* | 11 | gonococcal* OR gonorrh* |
|  | 12 | #5 AND #11 |
|  | 13 | gono* OR neisseria |
|  | 14 | #10 AND #13 |
|  | 15 | gonorrhea* |
|  | **16** | **#12 OR #14 OR #15** |
| *Herpes* | 17 | herpe* |
|  | 18 | genital* |
|  | **19** | **#17 AND (#5 OR #18)** |
| *Chlamydia* | 20 | chlamydia* |
|  | 21 | trachomatis* |
|  | 22 | salpingitis |
|  | 23 | #20 AND (#21 OR #22) |
|  | 24 | #10 AND (#20 OR #21) |
|  | 25 | lymphogranuloma* AND venereum* |
|  | **26** | **#23 OR #24 OR #25** |
| *Syphilis* | 27 | chancroid* OR "soft chancre" |
|  | 28 | "ulcus molle" |
|  | 29 | syphili* |
|  | 30 | "treponema pallidum" OR "T. pallidum" OR "T pallidum" |
|  | **31** | **#27 OR #28 OR #29 OR #30** |
| *HIV* | 32 | human |
|  | 33 | virus OR syndrome |
|  | 34 | #32 AND #33 |
|  | 35 | acquired |
|  | 36 | #34 OR #35 |
|  | 37 | immunodeficiency OR "immuno-deficiency" OR "immune-deficiency" OR immunedeficiency OR "immune deficiency" |
|  | 38 | #36 AND #37 |
|  | 39 | AIDS* |
|  | 40 | infection OR epidemic OR pandemic |
|  | 41 | #39 AND (#37 OR #40) |
|  | 42 | PLHIV* OR PLWHIV* OR HIV* |
|  | 43 | "tb-hiv" OR tbhiv OR "aids-virus" |
|  | **44** | **#38 OR #41 OR #42 OR #43** |
|  | **45** | **#5 OR #10 OR #16 OR #19 OR #26 OR #31 OR #44** |
| *Use barrier device* | 46 | use OR user* OR usage |
|  | 47 | wear* |
|  | 48 | utiliz* OR utilis* OR applicat* |
|  | 49 | #46 OR #47 OR #48 |
|  | 50 | barrier* OR device* |
|  | 51 | sex* |
|  | 52 | #49 AND #50 AND #51 |
| *sexual behavior* | 53 | "sexual behavior" OR "sexual behaviour" OR "sexual-behavior" OR "sexual-behaviour" |
|  | 54 | "safe sex" OR "safer sex" OR "unsafe sex" OR "responsible sex" |
|  | 55 | "high risk" AND behavio* AND sex* |
|  | 56 | #53 OR #54 OR #55 |
|  | **57** | **#52 OR #56** |
| *Digital interventions and techniques* | 58 | digital* OR online OR "web based" OR "web-based" OR (web AND portal) |
|  | 59 | mhealth* OR "m-health" OR "m health" OR ehealth* OR "e-health" OR "e health" OR "e-mental" |
|  | 60 | mobile* OR computer* OR "social media" |
|  | 61 | apps OR app OR "app-based" OR interactive* |
|  | 62 | #58 OR #59 OR #60 OR #61 |
|  | 63 | (text* OR short*) AND messag* |
|  | 64 | SMS* |
|  | 65 | #63 OR #64 |
|  | **66** | **#62 OR #65** |
| *Telehealth* | 67 | tele* OR videoconf* OR (video* AND conferenc*) |
|  | 68 | remote* OR (distance* AND manage*) OR "internet-delivered" |
|  | 69 | #67 OR #68 |
|  | 70 | tablets OR handheld* |
|  | 71 | device* OR computer* |
|  | 72 | #70 AND #71 |
|  | **73** | **#69 AND #71** |
|  | **74** | **#66 OR #73** |
| *Systematic review* | 75 | "critical review" OR "systematic quantitative review" OR "systematically searched" OR "systemic review" OR (review AND randomized) OR (systematic AND review) |
|  | 76 | "evidence-based analysis" OR "evidence-based review" |
|  | 77 | "electronic search" OR "literature search" OR MEDLINE OR "literature review" OR PubMed |
|  | 78 | "meta analysis" OR "meta synthesis" OR "meta-analyse" OR "meta-analytic review" OR "meta-study" OR "meta-synthesis" OR "metaanalysis" OR "metasynthesis" OR "meta-analysis" |
|  | 79 | "pooled effect" OR "random-effects model" |
|  | **80** | **#75 OR #76 OR #77 OR #78 OR #79** |
| **Query** | **81** | **(#45 OR #57) AND #74 AND #80** |

Note: Search strategies were adapted for each database (MEDLINE via PubMed, Cochrane Database of Systematic Reviews, Epistemonikos, and PsycINFO) using appropriate field tags and syntax. The full line-by-line strategies and database-specific modifications are available upon request or in Section 6.

Section 6 – Strategy in block format

Search strategies were developed and reported according to the PRISMA-S checklist to ensure transparency and reproducibility.

The initial search was executed on August 31, 2024, and rerun on November 12, 2025, following PRISMA-S recommendations for updated searches.

Strategies were adapted to the syntax and field tags of each database (PubMed/MEDLINE, Cochrane Library, PsycINFO, and Epistemonikos) while maintaining identical Boolean logic.

(((((((sexual* AND transmit) OR "sexually-transmitted") AND (infection OR disease)) OR stis OR stds OR sti OR std OR venereal) AND (gonococcal OR gonorrh)) OR gonorrhea OR ((gono* OR neisseria) AND ((pelvic* AND inflammatory) OR (upper AND genital* AND tract AND (infection* OR disease)) OR PID)) OR (chlamydia AND trachomatis* AND (infection* OR disease)) OR (lymphogranuloma AND venereum) OR (chlamyd AND salpingitis) OR ((chlamydia* OR trachomatis) AND ((pelvic AND inflammatory) OR (upper AND genital* AND tract AND (infection* OR disease)) OR PID)) OR chancroid OR "soft chancre" OR "ulcus molle" OR syphili* OR "treponema pallidum" OR "T. pallidum" OR "T pallidum" OR HIV* OR (((human AND (virus OR syndrome)) OR acquired) AND (immunodeficiency OR "immuno-deficiency" OR "immune-deficiency" OR immunedeficiency OR "immune deficiency")) OR (AIDS* AND (immunodeficiency OR "immuno-deficiency" OR "immune-deficiency" OR immunedeficiency OR "immune deficiency" OR infection OR epidemic OR pandemic)) OR PLWHIV OR "tb-hiv" OR tbhiv OR (genital* AND herpe) OR (herpes AND ((((sexual* AND transmit) OR "sexually-transmitted") AND (infection OR disease)) OR stis OR stds OR sti OR std OR venereal)) OR (((sexual AND transmit) OR "sexually-transmitted") AND (infection OR disease)) OR stis OR stds OR sti OR std OR venereal) AND (digital OR mhealth* OR "m-health" OR "m health" OR ehealth* OR "e-health" OR "e health" OR mobile* OR "web based" OR "web-based" OR "e-mental" OR (web AND portal) OR apps OR app OR "app-based" OR (text* AND messag) OR SMS OR (short* AND messag) OR "social media" OR computer OR online* OR interactive* OR tele* OR videoconf* OR (video* AND conferenc) OR remote OR (distance* AND manage) OR "internet-delivered" OR mobile OR phone* OR "cellular phone" OR "cell phone" OR "cell-phone" OR handphone* OR "hand phone" OR "hand-phone" OR "pocket phone" OR "pocket-phone" OR mhealth* OR "m-health" OR ((short OR multimedia) AND messag AND service) OR SMS OR MMS OR (smart AND phone) OR smartphone* OR "smart-phone" OR "smart phone" OR telephone* OR ((tablets OR handheld) AND (device OR computer)) OR tablets OR (tablet AND comput) OR iPad OR iOS* OR Proloquo2Go* OR iPod* OR (game* AND (video* OR serious)) OR DBCI)) AND ("critical review" OR "electronic search" OR "evidence-based analysis" OR "evidence-based review" OR "literature search" OR "meta analysis" OR "meta synthesis" OR "meta-analyse" OR "meta-analytic review" OR "meta-study" OR "meta-synthesis" OR "metaanalysis" OR "metasynthesis" OR "meta-analysis" OR "pooled effect" OR "random-effects model" OR "systematic quantitative review" OR "systematically searched" OR "systemic review" OR (review AND randomized) OR (systematic AND review) OR MEDLINE OR "literature review" OR PubMed)

**Strategy for Pubmed/MEDLINE**

#1113

**Strategy for Cochrane Library**

#148

**Strategy for PsycInfo**

#161

**Strategy for Epistemonikos** #1513

Note: Database-specific records retrieved were #1113 for PubMed/MEDLINE, #148 for the Cochrane Library, #161 for PsycINFO, and #1513 for Epistemonikos. No limits were applied regarding publication date, language, or country of origin.

Rerun on November 12, 2025

**Strategy for Epistemonikos** #378

#

# Section 7 – List of excluded studies

|  | **Author and year** | **Title** | **Reason** | **Justified Reason*** | **Comments** |
| --- | --- | --- | --- | --- | --- |
| 1 | Adebayo E, 2023 | Scalability of mobile technology interventions in the prevention and management of HIV among adolescents in low- and middle-income countries: protocol for a systematic review. | Wrong Design | 1 | Not a systematic review; it is a protocol for a systematic review. |
| 2 | Ameri A, 2020 | Impact of Mobile Phone-Based Interventions on Methamphetamine Use and High-risk Sexual Behaviors in Men Who Have Sex with Men (MSM): A Systematic Review. | Wrong Outcome | 4 | Does not focus on reducing sexual behaviors or does not inform on behavior change techniques. |
| 3 | Ana Carolina Maria Araújo Chagas Costa Lima, 2018 | Tecnologias e práticas educativas para prevenção da transmissão vertical do HIV. | Wrong Outcome | 4 | Focuses on vertical transmission, not on reducing sexual risk behaviors or relevant outcomes. |
| 4 | Atkins K, 2020 | Service delivery interventions to increase uptake of voluntary medical male circumcision for HIV prevention: A systematic review. | Wrong Outcome | 4 | Does not report on outcomes related to reducing sexual risk behaviors or behavior change techniques. |
| 5 | Bauermeister JA, 2017 | Addressing engagement in technology-based behavioural HIV interventions through paradata metrics. | Wrong Outcome | 4 | Focuses on engagement metrics rather than on reducing sexual behaviors or behavior change techniques. |
| 6 | Bragazzi N, 2023 | Queering Artificial Intelligence: The Impact of Generative Conversational AI on the Queer Community. A Scoping Review. | Wrong Outcome | 4 | The study does not focus on reducing sexual risk behaviors or behavior change techniques related to HIV/STI prevention. |
| 7 | Brown, William III, 2011 | New media interventions in youth sexual health promotion and HIV/STI prevention. | Wrong Design | 1 | Not a systematic review; appears to be more of a narrative or commentary on interventions. |
| 8 | Bumgarner KF, 2017 | Interventions that increase the intention to seek voluntary HIV testing in young people: a review. | Wrong Intervention | 3 | Does not report on digital behavior change interventions or techniques. |
| 9 | C. Catalani, 2013 | mHealth for HIV Treatment & prevention: a systematic review of the literature | Wrong Outcome | 4 | Focuses on treatment and care rather than on prevention and behavior change. |
| 10 | Cantor AG, 2023 | Telehealth for Women's Preventive Services for Reproductive Health and Intimate Partner Violence: A Comparative Effectiveness Review. | Wrong Outcome | 4 | Focuses on telehealth outcomes related to reproductive health but not specifically on reducing sexual risk behaviors or behavior change. |
| 11 | Cao B, 2017 | Social Media Interventions to Promote HIV Testing, Linkage, Adherence, and Retention: Systematic Review and Meta-Analysis. | Wrong Outcome | 4 and 2 | Primarily focuses on testing and treatment linkage rather than on prevention or behavior change related to sexual behaviors. |
| 12 | Cardwell ET, 2023 | Web-Based STI/HIV Testing Services Available for Access in Australia: Systematic Search and Analysis. | Wrong Outcome | 4 | Focuses on access to testing services, not on behavior change interventions aimed at reducing sexual risk behaviors. |
| 13 | Carvalho, Isaiane da Silva, 2020 | Educational technologies on sexually transmitted infections for incarcerated women | Wrong Outcome | 4 | Does not focus on digital behavior change interventions or outcomes related to reducing sexual risk behaviors. |
| 14 | Chavez N.R., 2013 | Use of digital media technology for primary prevention of STIS/HIV in adolescents and young adults: A systematic review of the literature | Wrong Design | 1 | Not a systematic review; the design does not fit the criteria for inclusion. |
| 15 | Choi SK, 2023 | Paradata: Measuring Engagement in Digital HIV Interventions for Sexual and Gender Minorities. | Wrong Outcome | 4 | Focuses on engagement data rather than on reducing sexual risk behaviors or behavior change techniques. |
| 16 | Craig Rushing, Stephanie Nicole, 2011 | Use of media technologies by Native American teens and young adults: Evaluating their utility for designing culturally-appropriate sexual health interventions targeting Native youth in the Pacific Northwest. | Wrong Design | 1 | Not a systematic review; more of a narrative exploration of media technologies. |
| 17 | Daher J, 2017 | Do digital innovations for HIV and sexually transmitted infections work? Results from a systematic review (1996-2017). | Wrong Outcome | 4 | Focuses on innovation in digital tools rather than on behavior change or reduction in sexual risk behaviors. |
| 18 | Daniel-Ulloa J, 2016 | Behavioral HIV Prevention Interventions Among Latinas in the US: A Systematic Review of the Evidence. | Wrong Outcome | 4 | Does not focus on digital interventions or outcomes related to behavior change. |
| 19 | Déglise C, 2012 | SMS for disease control in developing countries: a systematic review of mobile health applications. | Wrong Outcome | 4 | Focuses on disease control in general, not on sexual risk behaviors or behavior change interventions. |
| 20 | Duarte G, 2019 | Effectiveness of digital interventions based on mobile phones for the prevention of sexually transmitted infections: A systematic review protocol | Wrong Design | 1 | Not a systematic review; it is a protocol for a systematic review. |
| 21 | Escarfuller SG, 2023 | HIV Prevention Intervention-related Research with Adult, Sexual Minority Hispanic Men in the United States: A Systematic Review. | Wrong Intervention | 3 | Does not report on digital behavior change interventions; focuses on other types of interventions. |
| 22 | Escobar-Viera CG, 2021 | A systematic review of the engagement with social media-delivered interventions for improving health outcomes among sexual and gender minorities. | Wrong Intervention | 3 | Focuses on engagement rather than on behavior change or outcomes related to sexual risk behaviors. |
| 23 | Eshun-Wilson I, 2021 | A Systematic Review and Network Meta-analyses to Assess the Effectiveness of Human Immunodeficiency Virus (HIV) Self-testing Distribution Strategies. | Wrong Intervention | 3 | Focuses on self-testing distribution strategies rather than on behavior change interventions. |
| 24 | Forrest JI, 2015 | Mobile health applications for HIV prevention and care in Africa. | Wrong Intervention | 3 | Focuses on care rather than on prevention or behavior change interventions related to sexual behaviors. |
| 25 | Franco Vega I, 2022 | Using Video Games to Improve the Sexual Health of Young People Aged 15 to 25 Years: Rapid Review. | Wrong Intervention | 3 | Does not specify the use of digital behavior change techniques; focuses on gaming as a general tool. |
| 26 | Gabarron E, 2016 | Use of social media for sexual health promotion: a scoping review. | Wrong Intervention | 3 | Does not provide sufficient detail on behavior change techniques or their effectiveness in sexual health promotion. |
| 27 | Garrison LE, 2021 | Pre-exposure Prophylaxis Uptake, Adherence, and Persistence: A Narrative Review of Interventions in the U.S. | Wrong Intervention | 3 | Focuses on PrEP adherence rather than on behavior change interventions for sexual risk reduction. |
| 28 | Geldsetzer P, 2023 | Healthcare provider-targeted mobile applications to diagnose, screen, or monitor communicable diseases of public health importance in low- and middle-income countries: A systematic review. | Wrong Intervention | 3 | Focuses on diagnostic and screening tools rather than on behavior change interventions. |
| 29 | Gilbey D, 2020 | Effectiveness, Acceptability, and Feasibility of Digital Health Interventions for LGBTIQ+ Young People: Systematic Review. | Wrong Outcome | 4 | Does not report on outcomes related to sexual behavior change or STI prevention. |
| 30 | Goldstein M, 2023 | Systematic Review of mHealth Interventions for Adolescent and Young Adult HIV Prevention and the Adolescent HIV Continuum of Care in Low to Middle Income Countries. | Wrong Intervention | 3 | Focuses on general mHealth interventions without specific emphasis on behavior change techniques. |
| 31 | Hengel B, 2013 | Outreach for chlamydia and gonorrhoea screening: a systematic review of strategies and outcomes. | Wrong Intervention | 3 | Focuses on screening strategies rather than on behavior change interventions for sexual risk reduction. |
| 32 | Hightow-Weidman LB, 2020 | Engagement in mHealth behavioral interventions for HIV prevention and care: making sense of the metrics. | Wrong Intervention | 3 | Focuses on engagement metrics without specific emphasis on behavior change techniques or outcomes. |
| 33 | Hong C, 2022 | Technology-Based Interventions to Promote the HIV Preexposure Prophylaxis (PrEP) Care Continuum: Protocol for a Systematic Review. | Wrong Design | 1 | Not a systematic review; it is a protocol for a systematic review. |
| 34 | Horvath KJ, 2020 | A Systematic Review of Technology-Assisted HIV Testing Interventions. | Wrong Outcome | 4 | Focuses on testing interventions rather than on sexual behavior change outcomes. |
| 35 | Hou SI, 2014 | Systematic literature review of Internet interventions across health behaviors. | Wrong Outcome | 4 | Broad focus on various health behaviors, not specifically on sexual risk reduction or behavior change related to STIs/HIV. |
| 36 | Iyamu I, 2023 | Differential uptake and effects of digital sexually transmitted and bloodborne infection testing interventions among equity-seeking groups: a scoping review. | Wrong Intervention | 3 | Focuses on implementation outcomes rather than on behavior change or sexual risk reduction interventions. |
| 37 | Kamitani E, 2023 | Systematic review of alternative HIV pre-exposure prophylaxis (PrEP) care delivery models to improve PrEP services. | Wrong Intervention | 3 | Focuses on care delivery models rather than on behavior change interventions for sexual risk reduction. |
| 38 | Kaur P, 2021 | Use of eHealth for HIV Medical Education: A Narrative Review. | Wrong Intervention | 3 | Focuses on medical education rather than on behavior change or sexual risk reduction interventions. |
| 39 | Ko JS, 2020 | Mobile health promotion of human immunodeficiency virus self-testing in the United States. | Wrong Intervention | 3 | Focuses on promoting self-testing rather than on behavior change interventions for sexual risk reduction. |
| 40 | Kocur W, 2024 | Improving digital partner notification for sexually transmitted infections and HIV through a systematic review and application of the Behaviour Change Wheel approach. | Wrong Intervention | 3 | Focuses on partner notification rather than on behavior change interventions or outcomes related to sexual risk behaviors. |
| 41 | Kudrati SZ, 2021 | Social Media & PrEP: A Systematic Review of Social Media Campaigns to Increase PrEP Awareness & Uptake Among Young Black and Latinx MSM and Women. | Wrong Intervention | 3 | Focuses on PrEP awareness campaigns rather than on behavior change interventions. |
| 42 | L'Engle KL, 2016 | Mobile Phone Interventions for Adolescent Sexual and Reproductive Health: A Systematic Review. | Wrong Intervention | 3 | Focuses on reproductive health rather than on sexual risk reduction or behavior change interventions. |
| 43 | Lameiras-Fernandez M., 2021 | Sex education in the spotlight: What is working? systematic review | Wrong Design | 1 | Not a systematic review of digital interventions; it is an overview of systematic reviews and meta-analyses. |
| 44 | Long L, 2016 | Brief interventions to prevent sexually transmitted infections suitable for in-service use: A systematic review. | Wrong Intervention | 3 | Focuses on brief interventions without specific emphasis on digital behavior change interventions. |
| 45 | Lunny C, 2014 | Short message service (SMS) interventions for the prevention and treatment of sexually transmitted infections: a systematic review protocol. | Wrong Design | 1 | Not a systematic review; it is a protocol. The final review is included in Taylor et al., 2019. |
| 46 | Maksud I, 2015 | Technologies for HIV prevention and care: challenges for health services. | Wrong Design | 1 | Scoping review; reflects on the use of health technologies for HIV prevention and care without a systematic evaluation of effectiveness. |
| 47 | Maloney KM, 2020 | Electronic and other new media technology interventions for HIV care and prevention: a systematic review. | Wrong Intervention | 3 | Describes the use of digital interventions without assessing their effectiveness or behavior change techniques. |
| 48 | Marzan-Rodriguez M, 2021 | Recommendations for the Development of HIV Prevention Interventions Among Latino Young Sexual Minority Groups. | Wrong Intervention | 3 | Focuses on general recommendations for HIV prevention rather than on specific digital behavior change interventions. |
| 49 | McGuire M, 2021 | HIV self-testing with digital supports as the new paradigm: A systematic review of global evidence (2010-2021). | Wrong Intervention | 3 | Focuses on self-testing rather than on behavior change interventions for sexual risk reduction. |
| 50 | Meiksin R, 2021 | Theories of change for e-health interventions targeting HIV/STIs and sexual risk, substance use and mental ill health amongst men who have sex with men: systematic review and synthesis. | Wrong Intervention | 3 | Focuses on theories of change without providing detailed evaluation of the effectiveness of interventions. |
| 51 | Meiksin R, 2022 | E-health interventions targeting STIs, sexual risk, substance use and mental health among men who have sex with men: four systematic reviews | Wrong Intervention | 3 | Book that summarizes 4 systematic reviews. Chapter 7 discusses outcomes; three studies could be included in a synthesis. |
| 52 | Melendez-Torres GJ, 2022 | eHealth Interventions to Address HIV and Other Sexually Transmitted Infections, Sexual Risk Behavior, Substance Use, and Mental Ill-health in Men Who Have Sex with Men: Systematic Review and Meta-analysis. | Wrong Intervention | 3 | Focuses broadly on eHealth interventions without specific emphasis on digital behavior change techniques for sexual risk reduction. |
| 53 | van‐Velthoven, 2013 | Telephone delivered interventions for preventing HIV infection in HIV‐negative persons | Wrong Outcome | 4 | Focuses on telephone-based interventions without addressing sexual behavior change outcomes. |
| 54 | Mitchell JW, 2015 | The Use of Technology to Advance HIV Prevention for Couples. | Wrong Intervention | 5 | Scoping review; does not address the effectiveness or specific behavior change techniques within digital interventions. |
| 55 | Mora C, 2018 | HIV Testing Among “MSM”: Prevention Technologies, Sexual Moralities and Serologic Self-surveillance | Wrong Intervention | 5 | Does not focus on digital behavior change techniques or their effectiveness in sexual health promotion. |
| 56 | Mude W, 2023 | HIV Testing Disruptions and Service Adaptations During the COVID-19 Pandemic: A Systematic Literature Review. | Wrong Intervention | 3 | Focuses on service adaptations rather than on digital behavior change interventions for sexual risk reduction. |
| 57 | Mudhune V, 2022 | The Efficacy of a Smartphone Game to Prevent HIV Among Young Africans: Protocol for a Randomized Controlled Trial in the Context of COVID-19. | Wrong Design | 1 | Not a systematic review; it is a protocol for a randomized controlled trial (RCT). |
| 58 | Muessig K.E., 2015 | A Systematic Review of Recent Smartphone, Internet and Web 2.0 Interventions to Address the HIV Continuum of Care | Wrong Outcome | 4 | Focuses on treatment (TAR); same as Cao B., 2017. Muessig is a coauthor of that study. |
| 59 | Muessig KE, 2020 | Increasing HIV Testing and Viral Suppression via Stigma Reduction in a Social Networking Mobile Health Intervention Among Black and Latinx Young Men and Transgender Women Who Have Sex with Men (HealthMpowerment): Protocol for a Randomized Controlled Trial. | Wrong Design | 1 | Not a systematic review; it is a protocol for a randomized controlled trial. |
| 60 | Mukund Bahadur KC, 2010 | Cell phone short messaging service (SMS) for HIV/AIDS in South Africa: a literature review. | Wrong Outcome | 4 | Focuses on treatment (TAR); similar to Cao B., 2017. Muessig is a coauthor. |
| 61 | Musindo O, 2023 | Mental health and psychosocial interventions integrating sexual and reproductive rights and health, and HIV care and prevention for adolescents and young people (10-24 years) in sub-Saharan Africa: a systematic scoping review. | Wrong Intervention | 3 | Focuses on mental health and sexual and reproductive health (SRH) rather than on digital behavior change interventions. |
| 62 | Mustanski B, 2024 | A Systematic Review of Implementation Research on Determinants and Strategies of Effective HIV Interventions for Men Who Have Sex with Men in the United States. | Wrong Intervention | 3 | Important for discussion on the implementation of strategies in medical settings. |
| 63 | Nelson KM, 2020 | A systematic review of mHealth interventions for HIV prevention and treatment among gay, bisexual, and other men who have sex with men. | Wrong Outcome | 4 | Focuses on both treatment and prevention, but the emphasis on treatment disqualifies it for inclusion in studies focused on behavior change. |
| 64 | Niakan S, 2017 | Web and Mobile Based HIV Prevention and Intervention Programs Pros and Cons - A Review. | Wrong Intervention | 3 | Important for introduction and discussion, especially in justifying the use of eHealth in HIV/STD prevention. |
| 65 | Nourimand F, 2022 | A systematic review of eHealth modes in preventing sexually transmitted infections. | Wrong Intervention | 3 | Scoping review; describes the use of eHealth without focusing on effectiveness or behavior change techniques used in interventions. |
| 66 | Nwaozuru U, 2021 | Mobile health interventions for HIV/STI prevention among youth in low- and middle-income countries (LMICs): a systematic review of studies reporting implementation outcomes. | Wrong Intervention | 3 | Focuses on implementation outcomes (acceptability, adoption) rather than on behavior change or sexual risk reduction. |
| 67 | Onukwugha FI, 2022 | The effectiveness and characteristics of mHealth interventions to increase adolescent's use of Sexual and Reproductive Health services in Sub-Saharan Africa: A systematic review. | Wrong Intervention | 3 | Includes studies on breastfeeding, pregnancy, and TAR treatment; relevant studies on sexual behavior can be separated for reporting. |
| 68 | Orta Portillo GA, 2023 | Virtual avatars as a new tool for human immunodeficiency virus prevention among men who have sex with men: a narrative review. | Wrong Intervention | 3 | Does not describe effects or specify content based on theoretical frameworks or behavior change techniques (BCTs). |
| 69 | Paschen-Wolff MM, 2019 | A Systematic Review of Interventions that Promote Frequent HIV Testing. | Wrong Intervention | 3 | Focuses on testing frequency rather than on behavior change interventions for sexual risk reduction. |
| 70 | Patel P, 2022 | Public health implications of adapting HIV pre-exposure prophylaxis programs for virtual service delivery in the context of the COVID-19 pandemic: a systematic review. | Wrong Intervention | 3 | Focuses on virtual service delivery without specific emphasis on digital behavior change interventions for sexual risk reduction. |
| 71 | Queiroz AAFLN, 2021 | mHealth Strategies Related to HIV Postexposure Prophylaxis Knowledge and Access: Systematic Literature Review, Technology Prospecting of Patent Databases, and Systematic Search on App Stores. | Wrong Intervention | 3 | Focuses on post-exposure prophylaxis (PEP) rather than on behavior change interventions for sexual risk reduction. |
| 72 | Ritchwood TD, 2019 | "Getting to Zero" Among Men Who Have Sex with Men in China: A Review of the HIV Care Continuum. | Wrong Intervention | 3 | Focuses on the HIV care continuum rather than on behavior change interventions for sexual risk reduction. |
| 73 | Rodrigues J., 2021 | Starting and staying on PrEP: A scoping review of strategies for supporting and improving effective use of PrEP | Wrong Design | 1 | Not a systematic review; it is a scoping review without a focus on digital behavior change interventions. |
| 74 | Ronen K, 2020 | Peer Group Focused eHealth Strategies to Promote HIV Prevention, Testing, and Care Engagement. | Wrong Intervention | 3 | Focuses on peer group strategies without specific emphasis on digital behavior change techniques. |
| 75 | Rosen AO, 2024 | Efficacy of behavioral interventions to increase engagement in sexual health services among LatinX youth in the United States: A meta-analysis for post-pandemic implications. | Wrong Intervention | 3 | Does not report on the use of digital interventions specifically. |
| 76 | Saif-Ur-Rahman KM, 2023 | Artificial intelligence and digital health in improving primary health care service delivery in LMICs: A systematic review. | Wrong Intervention | 3 | Only 1 out of 48 studies included focuses on HIV prevention; primarily addresses general health care services with AI. |
| 77 | Sales, Rafaela Oliveira de, 2020 | mHealth in the prevention of sexually transmitted infections STIs | Wrong Intervention | 3 | Focuses broadly on mHealth without specific emphasis on digital behavior change techniques for STI prevention. |
| 78 | Sanz-Lorente M, 2018 | Web 2.0 Tools in the Prevention of Curable Sexually Transmitted Diseases: Scoping Review. | Wrong Intervention | 3 | Interesting for discussion on the use of social media platforms like Facebook, Instagram, etc., but does not focus on behavior change techniques. |
| 79 | Silverman T, 2018 | Quality of HIV Websites with Information About Pre-Exposure Prophylaxis or Treatment as Prevention for Men Who Have Sex with Men: Systematic Evaluation. | Wrong Intervention | 4 | Focuses on quality assessment of information rather than on behavior change or sexual risk reduction outcomes. |
| 80 | Simoni JM, 2018 | Health Behavior Theory to Enhance eHealth Intervention Research in HIV: Rationale and Review. | Wrong Intervention | 3 | Critical for the discussion on theoretical foundations but does not evaluate specific behavior change techniques. |
| 81 | Skeen SJ, 2021 | mHealth for transgender and gender-expansive youth: harnessing gender-affirmative cross-disciplinary innovations to advance HIV prevention and care interventions. | Wrong Intervention | 3 | Scoping review; discusses theories broadly without evaluating the effects of each intervention separately. |
| 82 | Smith AU, 2023 | A Review of Serious Gaming as an Intervention for HIV Prevention. | Wrong Intervention | 3 | Does not specify behavior change techniques used in the serious games; useful for discussion. |
| 83 | Taggart T, 2015 | Social media and HIV: A systematic review of uses of social media in HIV communication. | Wrong Intervention | 3 | Focuses on the use of social media in communication rather than on behavior change interventions. |
| 84 | Taylor D, 2019 | Effectiveness of text messaging interventions on prevention, detection, treatment, and knowledge outcomes for sexually transmitted infections (STIs)/HIV: a systematic review and meta-analysis. | Wrong Intervention | 3 | Does not evaluate behavior change techniques; however, it is a strong study for discussion and meta-analysis. |
| 85 | Teadt S, 2020 | African American Adolescents and Young Adults, New Media, and Sexual Health: Scoping Review. | Wrong Intervention | 3 | Scoping review; discusses the potential of new media but does not evaluate behavior change techniques. |
| 86 | Vanhamel J, 2020 | The current landscape of pre-exposure prophylaxis service delivery models for HIV prevention: a scoping review. | Wrong Intervention | 3 | Reviews existing service models for PrEP delivery, with less emphasis on behavior change interventions. |
| 87 | Velthoven MH, 2012 | Telephone consultation for improving health of people living with or at risk of HIV: a systematic review (Structured abstract) | Wrong Design | 1 | Abstract; insufficient detail on intervention design or outcomes. |
| 88 | Velthoven MH, 2013 | Scope and effectiveness of mobile phone messaging for HIV/AIDS care: A systematic review. | Wrong Intervention | 3 | Broad focus on the scope and effectiveness of messaging without specific evaluation of behavior change techniques. |
| 89 | Versluis A, 2022 | Direct Access for Patients to Diagnostic Testing and Results Using eHealth: Systematic Review on eHealth and Diagnostics. | Wrong Intervention | 5 | Focuses on direct access to diagnostics rather than on interventions related to sexual risk behavior change. |
| 90 | Vialard F, 2023 | Self-sampling strategies (with/without digital innovations) in populations at risk of Chlamydia trachomatis and Neisseria gonorrhoeae: a systematic review and meta-analyses. | Wrong Intervention | 5 | Includes both digital and non-digital interventions; evaluates acceptability without focusing on behavior change techniques. |
| 91 | Wadham E, 2019 | New digital media interventions for sexual health promotion among young people: a systematic review. | Wrong Intervention | 3 | Focuses on the use of digital media for promotion without evaluating specific behavior change techniques or outcomes. |
| 92 | Wagner AD, 2023 | Demand creation for HIV testing services: A systematic review and meta-analysis. | Wrong Intervention | 5 | Focuses on demand creation for testing rather than on behavior change interventions for sexual risk reduction. |
| 93 | Wang L, 2023 | Effect of e-health interventions on HIV prevention: a protocol of systematic review and meta-analysis. | Wrong Design | 1 | Not a systematic review; it is a protocol for a systematic review. |
| 94 | Wang Y, 2022 | Evidence and implication of interventions across various socioecological levels to address pre-exposure prophylaxis uptake and adherence among men who have sex with men in the United States: a systematic review. | Wrong Outcome | 4 | Focuses on improving PrEP uptake rather than on sexual risk behavior change; does not inform on behavior change techniques. |
| 95 | Wang Y, 2022 | Evidence and implication of interventions across various socioecological levels to address HIV testing uptake among men who have sex with men in the United States: A systematic review. | Wrong Outcome | 4 | Focuses on testing uptake rather than on sexual risk behavior change or behavior change techniques. |
| 96 | Wei, Chongyi, 2011 | Social marketing interventions to increase HIV/STI testing uptake among men who have sex with men and male‐to‐female transgender women | Wrong Outcome | 4 | Evaluates the impact of social marketing interventions on testing uptake without addressing behavior change techniques. |
| 97 | Whiteley L, 2020 | A Mobile Gaming Intervention for Persons on Pre-Exposure Prophylaxis: Protocol for Intervention Development and Randomized Controlled Trial. | Wrong Design | 1 | Not a systematic review; it is a protocol for a randomized controlled trial. |
| 98 | Ybarra ML, 2007 | Current trends in Internet- and cell phone-based HIV prevention and intervention programs. | Wrong Intervention | 3 | Focuses on trends without specific evaluation of behavior change techniques or their effectiveness. |
| 99 | Yeh PT, 2022 | Web-Based Service Provision of HIV, Viral Hepatitis, and Sexually Transmitted Infection Prevention, Testing, Linkage, and Treatment for Key Populations: Systematic Review and Meta-analysis. | Wrong Intervention | 3 | Focuses on service provision rather than on behavior change interventions. |
| 100 | Zanoni BC, 2018 | Screening for HIV and linkage to care in adolescents: insights from a systematic review of recent interventions in high- versus low- and middle-income settings. | Wrong Intervention | 3 | Focuses on screening and linkage to care rather than on behavior change interventions for sexual risk reduction. |
| 101 | Zou H, 2012 | The efficacy of clinic-based interventions aimed at increasing screening for bacterial sexually transmitted infections among men who have sex with men: a systematic review. | Wrong Intervention | 3 | Focuses on screening interventions without specific emphasis on behavior change techniques. |
| 102 | Παπαευθυμίου, Ευθύμιος, 2018 | Use of social media for sexual health promotion / Χρήση των μέσων κοινωνικής δικτύωσης για την προαγωγή της σεξουαλικής υγείας. | Wrong Intervention | 3 | Second version of another document. |
| 103 | Παπαευθυμίου, Ευθύμιος, 2018 | Χρήση των μέσων κοινωνικής δικτύωσης για την προαγωγή της σεξουαλικής υγείας. | Wrong Intervention | 3 | Article not available in English (in Greek). |
| 104 | Nicely p, 2025 | Approaches and impacts of digital health in HIV self-testing uptake & use among populations from low- and middle-income countries: a descriptive systematic review and meta-analysis | Wrong Outcome | 4 | Does not focus on reducing sexual behaviors or does not inform on behavior change techniques. |
| 105 | Vaikla O, 2025 | Digital Innovations for Youth Sexual and Reproductive Health Knowledge and Behaviour: Evidence from a Systematic Review | Wrong Design | 1 | Not a systematic review; it is a protocol for a systematic review. |
| 106 | Fischer AE, 2025 | Mobile Health Intervention Tools Promoting HIV Pre-Exposure Prophylaxis Among Adolescent Girls and Young Women in Sub-Saharan Africa: Scoping Review. | Wrong Design | 2 | Not a systematic review; it is a protocol for a systematic review. |
| *Justified Reason: 1. Not a systematic review (Commentary articles, conference articles, primary studies, others); 2. Not the population of interest (reviews focused on treatment and adherence to antiretroviral therapy (ART) and self-management of people living with HIV, other); 3- Not the intervention of interest ( Articles that do not report use of digital behavior change intervention);4. Not the outcome of interest (Reducing the risk of sexual behaviors, for example; Use of condoms, increase the use of STI testing or does not Inform on behavior change techniques or theory). The final three records (Nicely 2025; Vaikla 2025; Fischer 2025) were excluded after full-text assessment based on predefined eligibility criteria. Nicely (2025) was excluded due to focusing on outcomes unrelated to sexual risk reduction or behavior change techniques (Reason 4). Vaikla (2025) and Fischer (2025) were excluded because they did not meet the study design criterion, as both publications were protocols rather than completed systematic reviews (Reason 1). These exclusion decisions align with the justified reasons outlined below the table, which specify non-eligible study designs, populations, interventions, and outcomes. | | | | | |

# Section 8 – Intercoder reliability calculation report

Intercoder reliability was calculated only for the initial full-text screening set (n = 122), during which both reviewers independently assessed all articles.

The 7 additional records identified in the updated search were screened subsequently and resolved through discussion; therefore, they were not included in the Kappa calculation to preserve the assumption of independent ratings.

Data Overview:

The data used for the analysis is summarized in the following table:

|  | Coder 2 - Yes | Coder 2- No | Total |
| --- | --- | --- | --- |
| Coder 1- Yes | 19 | 2 | 21 |
| Coder 1- No | 2 | 99 | 101 |
| Total | 21 | 101 | 122 |

Calculation of Observed Agreement (Po):

The observed agreement (Po) is the proportion of cases where both coders agreed. The formula used is:
Po = (a + d) / N
Where:
- a = 19 (cases where both coders said "Yes")
- d = 99 (cases where both coders said "No")
- N = 122 (total cases)

The observed agreement is:
Po = 118 / 122 = 0.967
This indicates that the coders agreed in 96.7% of the cases.

Calculation of Expected Agreement (Pe):

The expected agreement (Pe) accounts for the agreement that could occur by chance. The formula used is:
Pe = [(a+b) * (a+c) + (c+d) * (b+d)] / N^2
Where:
- (a + b) = 21 (Coder 1 - Yes)
- (a + c) = 21 (Coder 2 - Yes)
- (c + d) = 101 (Coder 2 - No)
- (b + d) = 101 (Coder 1 - No)

The expected agreement is:
Pe = 0.7151

Calculation of Cohen's Kappa (K):

Cohen's Kappa is calculated to determine the agreement beyond chance. The formula used is:
K = (Po - Pe) / (1 - Pe)
Substituting the calculated values:
K = (0.967 - 0.7151) / 0.2849 = 0.8851

The interpretation of kappa values followed these ranges: < 0.00: Concordancia pobre (Poor agreement), 0.00–0.20: Concordancia ligera (Slight agreement), 0.21–0.40: Concordancia aceptable (Fair agreement), 0.41–0.60: Concordancia moderada (Moderate agreement), 0.61–0.80: Concordancia sustancial (Substantial agreement), 0.81–1.00: Concordancia casi perfecta (Almost perfect agreement).

Interpretation:

The Kappa value of 0.8851 suggests an almost perfect agreement between the coders. According to the standard interpretation of Kappa values, this level of agreement indicates that the coders' decisions are highly reliable and consistent.

# Section 9– Data extraction form

| **Excel Column** | **Description** |
| --- | --- |
| RefID | Assign a unique identifier for each systematic review. |
| Reviewer | Enter the name of the person reviewing the data. |
| Author and Year | Enter the author(s) and year of the study. Example: Bailey JV, 2021. |
| Title | Enter the title of the systematic review. Example: Interactive digital interventions for prevention of sexually transmitted HIV. |
| Aim of the SR | Summarize the aim of the systematic review. Example: To assess the effectiveness of interactive digital interventions (IDIs) for HIV prevention. |
| Number of Studies Included | Enter the total number of studies included in the systematic review. |
| Participants (Total Number) | Enter the total number of participants across all included studies. |
| Year of Most Recent Study | Enter the publication year of the most recent study included in the review. Example: 2017. |
| Search Period/Date | Provide the range of dates during which the literature was searched. Example: From 2014 to June 2017. |
| Geographic Location (Country) | List the countries where the studies were conducted. Example: USA, Netherlands, Uganda. |
| Only RCT | Select Yes or No depending on whether the review includes only RCTs. |
| Only non-RCT | Select Yes or No if the review includes only non-RCT studies (e.g., non-randomized, observational). |
| RCT and non-RCT | Select Yes or No if the review includes both RCT and non-RCT studies. |
| Number of RCTs | Enter the number of RCTs included in the review. Example: 31. |
| Population Type (Age Group) | Specify the age groups included, e.g., Adolescents, Youth, Adults. |
| Specific Populations | Indicate any specific populations targeted, such as MSM, LGTBIQ+, people with STDs, etc. |
| Target Behavior | Describe the behaviors targeted by the intervention, e.g., Prevention of STI/HIV, Promotion of safe sex, Diagnosis, Treatment adherence. |
| Intervention Environment | Specify the settings where interventions were implemented, e.g., Schools, Universities, Health Centers. |
| Behavior Change Theoretical Framework | Indicate whether an explicit behavior change framework was used. Select Yes or No. |
| Behavior Change Techniques (BCTs) | Describe the behavior change techniques used according to the BCTTv1 taxonomy. |
| Behavioral Outcomes | List the behavioral outcomes measured, such as condom use, frequency of unprotected intercourse, number of sexual partners, etc. |
| Cognitive Outcomes | Indicate any cognitive outcomes measured, such as self-efficacy, knowledge, attitudes towards condom use, etc. |
| Biological Outcomes | List any biological outcomes measured, such as HIV or STI acquisition. |
| APEASE Criteria | Evaluate the intervention based on the APEASE criteria (Acceptability, Practicability, Effectiveness, Affordability, Spill-over effects, Equity). |
| Technology Delivered | Specify the type of technology used, e.g., Mobile devices, Desktop computers, Text messaging, Video call, etc. |
| Digital Content Type | Describe the type of digital content used, e.g., Website, App, Video Game, Virtual Reality, AI-based Chatbots. |
| Risk of Bias Tool | Indicate the tool used for assessing the risk of bias, e.g., Cochrane risk of bias tool. |
| Meta-analysis Conducted | Select Yes or No if a meta-analysis was conducted. |
| Certainty of Evidence | Indicate the certainty of evidence, possibly using GRADE or other evaluation criteria. |
| Other Outcomes or Descriptions | Provide any other relevant outcomes or additional descriptions specific to the study. |

# Section 10 – Coding Manual for Study 1. Project Title: Digital Behavior Change Interventions to Prevent Sexually Transmitted Infections (STIs) Including HIV: Evidence Reviews and Integrated Report on the Quantitative and Qualitative Evidence.

*English version*

**1. Objective:**

This manual provides guidelines for coding data extraction from the studies included in this systematic review. It defines the variables to be coded, the different categories for each variable, and specifies which categorized variables remain open (expecting to identify additional categories not yet included in the manual) to support the work of both coders. Finally, this manual will be included as supplementary material in the publication.

**2. Description of Variables for Data Extraction**

**Identification and General Characteristics**

- **Episte ID:** Unique identifier assigned to each systematic review in the Epistemonikos database.
- **Author and Year:** Name of the first author and year of publication of the systematic review.
- **Aim of the SR:** The stated purpose of the systematic review, describing its focus or research question.
- **Title:** Full title of the systematic review.

**Systematic Review Design**

- **Number of Studies Included:** Total number of primary studies included in the systematic review.
- **Year of the Most Recent Study:** Year of publication of the most recent primary study included in the systematic review.
- **Place (Country - Geographic Location):** Country or geographic location where the included primary studies were conducted.
- **Included Designs:** Types of primary study designs included in the systematic review (e.g., randomized controlled trials (RCTs), observational studies, qualitative studies, etc.).
- **Number of RCTs:** Total number of randomized controlled trials included in the systematic review.

**Population Type**

- **Definition:** Characteristics of the population studied in the systematic review.
- **Possible Categories:** Adolescents, Young Adults, Adults, Men Who Have Sex with Men (MSM), LGBTQ+ Individuals, People with an STI Diagnosis, Other (describe).
- **Adolescent (Population Age):** Whether the population of interest includes adolescents, typically aged 10-19 years.
- **Young (Population Age):** Whether the population of interest includes young adults, typically aged 18-24 years.
- **Adults (Population Age):** Whether the population of interest includes adults, typically aged 18 and older.
- **Men Who Have Sex with Men (MSM):** Whether the systematic review focuses on men who have sex with other men.
- **LGBTQ+ Groups:** Whether the systematic review includes individuals identifying as LGBTQ+ (Lesbian, Gay, Bisexual, Transgender, Intersex, Queer, etc.).
- **People with an STI Diagnosis:** Whether the population of interest includes individuals diagnosed with sexually transmitted infections (STIs).
- **HIV:** Whether the population of interest includes people living with HIV.

**Intervention Characteristics**

- **Prevention of STIs:** Whether the systematic review focuses on STI prevention.
- **Promotion of Safe Sex Conducts:** Whether the systematic review promotes safe sexual behaviors.
- **Type and Characteristics of the Digital Intervention:** Description of the types and characteristics of digital interventions evaluated.
- **Environment Where Implemented:** Locations where the digital interventions were carried out (e.g., schools, universities, healthcare centers, etc.).
- **Mode of Delivery:** How the digital interventions were delivered (e.g., social media, videos, SMS, mobile phones, etc.).
- **Provision of an Explicit Behaviour Change Theoretical Domains Framework:** Whether the systematic review includes an explicit theoretical framework for behavior change.
- **Description of Framework or Theory as Reported by Authors:** Details on the theoretical framework or theory used in the systematic review.
- **BCTTv1:** Coding and mapping of behavior change techniques according to the Behaviour Change Techniques Taxonomy version 1.
- **TDF:** Data extraction related to the Theoretical Domains Framework (TDF).
- **Coding and Mapping According to BCW:** Coding and mapping of interventions according to the Behaviour Change Wheel (BCW).

**Behavioral Outcomes**

- **Condom Use (Internal or External):** Promotion and evaluation of condom use (male and female condoms).
- **Frequency of Unprotected Sexual Intercourse:** Frequency of unprotected sexual intercourse among participants.
- **Number of Sexual Partners:** Number of sexual partners within a given period.
- **STD/HIV Testing:** Whether interventions promote or provide access to STI/HIV testing.
- **Uptake of Medical Male Circumcision:** Acceptance or uptake of medical male circumcision.
- **HIV Counselling:** Whether interventions include counseling related to HIV prevention, testing, or management.
- **Vaccination Against HPV, Hepatitis A & B:** Whether interventions promote or facilitate vaccinations for HPV, Hepatitis A, or B.
- **Other Cognitive Outcomes (Description):** Any additional cognitive outcomes assessed.

**Cognitive Outcomes (Mediators of Prevention)**

- **Self-Efficacy:** The extent to which individuals feel capable of engaging in preventive behaviors (e.g., condom use, HIV testing).
- **STD/HIV-Related Knowledge:** Level of knowledge regarding STI/HIV transmission, prevention, and treatment.
- **Attitudes Towards Condom Use:** Attitudes and perceptions toward condom use as a preventive measure.
- **PrEP/PEP Awareness:** Awareness of and access to pre-exposure prophylaxis (PrEP) and post-exposure prophylaxis (PEP) for HIV prevention.
- **Other Cognitive Outcomes (Description):** Any additional cognitive outcomes assessed.

**Biological Outcomes**

- **HIV or STI Acquisition:** Evaluation of incidence or prevalence of HIV or STI acquisition.
- **Other Biological Outcomes (Description):** Any additional biological outcomes assessed.

**APEASE (Acceptability, Practicability, Effectiveness, Affordability, Spill-over Effects, and Equity)**

- **Acceptability:** Degree to which behavior change interventions are accepted by users and healthcare providers.
- **Practicability:** Feasibility and ease of implementation in real-world settings.
- **Effectiveness:** Extent to which interventions achieve their objectives and lead to behavior change.
- **Affordability:** Economic viability and accessibility of interventions.
- **Spill-Over Effects:** Unintended effects (both positive and negative) of interventions.
- **Equity:** Assessment of whether interventions promote health equity and address disparities.

**Technology Delivered**

- **Mobile Device:** Use of mobile phones, tablets, or portable devices.
- **Desktop Computer:** Use of desktop computers.
- **Digital Billboard:** Use of digital billboards for intervention delivery.
- **Wearable Accessory (Clothing/Accessory):** Use of wearable devices for intervention delivery.
- **Digital Object:** Use of physical objects embedded with digital technology.
- **Projection/Hologram:** Use of projected images or holograms.

**Digital Content Type**

- **Audio Call/Message:** Use of audio calls or voice messages.
- **Video Call/Message:** Use of video calls or video messages.
- **Text/Instant Message:** Use of text messages or instant messaging.
- **Email:** Use of email communication.
- **Video Game:** Use of serious games for health education.
- **Audio Broadcast/Podcast:** Use of podcasts or audio broadcasts.
- **Website/Computer Program/App:** Use of websites, computer programs, or mobile applications.
- **eBook:** Use of eBooks or digital reading materials.
- **Virtual or Augmented Reality:** Use of immersive experiences for education and intervention.
- **AI-Based Chatbots for Promoting Safe Sex:** Use of AI-driven chatbots for promoting safe sexual behaviors.

**Risk of Bias and Certainty of Evidence**

- **Risk of Bias Tool:** Tool used to assess bias in included studies.
- **Meta-Analysis:** Whether the review includes a meta-analysis.
- **Certainty of Evidence (GRADE):** Assessment of evidence quality using the GRADE approach (Grading of Recommendations, Assessment, Development, and Evaluations).

*Spanish version*

**Manual de Codificación para el Estudio 1**

**Título del Proyecto:** Intervenciones Digitales de Cambio de Comportamiento para la Prevención de Infecciones de Transmisión Sexual (ITS), Incluyendo VIH: Revisiones de Evidencia e Informe Integrado sobre la Evidencia Cuantitativa y Cualitativa

**1. Objetivo:**

Este manual proporciona directrices para la codificación de la extracción de datos de los estudios incluidos en esta revisión sistemática. Define las variables a codificar, las diferentes categorías de cada variable y especifica qué variables categorizadas permanecen abiertas (se espera identificar categorías adicionales aún no incluidas en el manual) para apoyar el trabajo de ambos codificadores. Finalmente, este manual será incluido como material suplementario en la publicación.

**2. Descripción de Variables para la Extracción de Datos**

**Identificación y Características Generales**

- **Episte ID:** Identificador único asignado a cada revisión sistemática en la base de datos Epistemonikos.
- **Autor y Año:** Nombre del primer autor y año de publicación de la revisión sistemática.
- **Objetivo de la RS:** Propósito declarado de la revisión sistemática, describiendo su enfoque o pregunta de investigación.
- **Título:** Título completo de la revisión sistemática.

**Diseño de la Revisión Sistemática**

- **Número de Estudios Incluidos:** Cantidad total de estudios primarios incluidos en la revisión sistemática.
- **Año del Estudio Más Reciente:** Año de publicación del estudio primario más reciente incluido en la revisión sistemática.
- **Lugar (País - Ubicación Geográfica):** País o ubicación geográfica donde se realizaron los estudios primarios incluidos en la revisión sistemática.
- **Diseños Incluidos:** Tipos de diseños de estudios primarios incluidos en la revisión sistemática (por ejemplo, ensayos controlados aleatorizados (ECA), estudios observacionales, estudios cualitativos, etc.).
- **Número de ECA:** Número total de ensayos controlados aleatorizados incluidos en la revisión sistemática.

**Tipo de Población**

- **Definición:** Características de la población estudiada en la revisión sistemática.
- **Posibles Categorías:** Adolescentes, Jóvenes, Adultos, Hombres que tienen sexo con hombres (HSH), Personas LGBTQ+, Personas con diagnóstico de ITS, Otros (describir).
- **Adolescentes (Edad de la Población):** Si la población de interés incluye adolescentes, generalmente definidos como personas entre 10 y 19 años.
- **Jóvenes (Edad de la Población):** Si la población de interés incluye adultos jóvenes, generalmente definidos como personas entre 18 y 24 años.
- **Adultos (Edad de la Población):** Si la población de interés incluye adultos, generalmente definidos como personas mayores de 18 años.
- **Hombres que tienen Sexo con Hombres (HSH):** Si la revisión sistemática se enfoca en hombres que tienen relaciones sexuales con otros hombres.
- **Grupos LGBTQ+:** Si la revisión sistemática incluye personas que se identifican como LGBTQ+ (Lesbianas, Gays, Bisexuales, Transgénero, Intersexuales, Queer, etc.).
- **Personas con Diagnóstico de ITS:** Si la población de interés incluye personas diagnosticadas con infecciones de transmisión sexual (ITS).
- **VIH:** Si la población de interés incluye personas que viven con VIH.

**Tipo de Intervención**

- **Prevención de ITS:** Si la revisión sistemática aborda la prevención de infecciones de transmisión sexual.
- **Promoción de Conductas Sexuales Seguras:** Si la revisión sistemática promueve comportamientos sexuales seguros.
- **Tipo y Características de la Intervención Digital:** Descripción de los tipos y características de las intervenciones digitales evaluadas.
- **Entorno de Implementación:** Lugares donde se realizaron las intervenciones digitales (por ejemplo, escuelas, universidades, centros de salud, etc.).
- **Modo de Entrega:** Forma en que se entregaron las intervenciones digitales (por ejemplo, redes sociales, videos, SMS, teléfonos móviles, etc.).
- **Provisión de un Marco Teórico Explícito de Cambio de Comportamiento:** Si la revisión sistemática incluye un marco teórico explícito sobre el cambio de comportamiento.
- **Descripción del Marco o Teoría según los Autores:** Detalles sobre el marco teórico o teoría utilizado en la revisión sistemática.
- **BCTTv1:** Codificación y mapeo de técnicas de cambio de comportamiento según la Taxonomía de Técnicas de Cambio de Comportamiento versión 1.
- **TDF:** Extracción de datos basada en los Dominios Teóricos del Cambio de Comportamiento (TDF).
- **Codificación y Mapeo según el BCW:** Codificación y mapeo de intervenciones según las funciones de intervención del BCW (Behavior Change Wheel).

**Resultados del Comportamiento**

- **Uso del Condón (Interno o Externo):** Promoción y evaluación del uso del condón (masculino y femenino).
- **Frecuencia de Relaciones Sexuales sin Protección:** Frecuencia de relaciones sexuales sin protección entre los participantes.
- **Número de Parejas Sexuales:** Número de parejas sexuales en un período determinado.
- **Pruebas de ITS/VIH:** Si las intervenciones promueven o facilitan pruebas de ITS/VIH.
- **Aceptación de la Circuncisión Médica Masculina:** Evaluación de la disposición hacia la circuncisión médica masculina.
- **Asesoramiento sobre VIH:** Si las intervenciones incluyen asesoramiento relacionado con la prevención, detección o manejo del VIH.
- **Vacunación Contra VPH, Hepatitis A y B:** Si las intervenciones promueven o facilitan la vacunación contra el VPH, Hepatitis A o B.
- **Otros Resultados Cognitivos (Descripción):** Otros resultados cognitivos evaluados.

**Evaluación del Riesgo de Sesgo y Certeza de la Evidencia**

- **Herramienta de Riesgo de Sesgo:** Herramienta utilizada para evaluar el sesgo en los estudios incluidos.
- **Metaanálisis:** Si la revisión incluye un metaanálisis.
- **Certeza de la Evidencia (GRADE):** Evaluación de la calidad y certeza de la evidencia utilizando el enfoque GRADE (Grading of Recommendations, Assessment, Development and Evaluations).

# Section 11 – Risk of Bias Assessment (Critical Appraisal of Systematic Reviews)

This is an example of using an Excel spreadsheet (version 10; Microsoft Inc.) for Risk of Bias Assessment (Critical Appraisal of Systematic Reviews) based on AMSTAR 2.

Reference:

Shea BJ, Reeves BC, Wells G, Thuku M, Hamel C, Moran J, Moher D, Tugwell P, Welch V, Kristjansson E, Henry DA. AMSTAR 2: a critical appraisal tool for systematic reviews that include randomised or non-randomised studies of healthcare interventions, or both. BMJ. 2017 Sep 21;358

|  | **A M S T A R 2** |  |  |  |  |
| --- | --- | --- | --- | --- | --- |
|  |  |  | **Author Year** | **Bailey JV, 2021** | |
| **Criteria** | **Question** | **Description** | **Description** | **Vote** | **Quotes** |
| 1. | Did the research questions and inclusion criteria for the review include the components of PICO? | **For yes:   - Population  - Intervention  - Comparator group  - Outcome** | Optional (recommended)  - Period for follow-up | YES |  |
| 2. | Did the report of the review contain an explicit statement that the review methods were established prior to the conduct of the review and did the report justify any significant deviations from the protocol? | **For Partial Yes: The authors state that they had written protocol or guide that included ALL the following:   - review question(s)  - a search strategy  - inclusion/exclusion criteria  - a risk of bias assessment** | **For Yes: As for partial yes, plus the protocol should be registered and should also have specfied:  - a meta-analysis/synthesis plan, if appropriate, and - a plan for investigating causes of heterogeneity - justification for any deviations from the protocol** | YES | It is an update of a Cochrane SR. It describes that they drafted a protocol, but it was not possible to access the protocol. |
| 3. | Did the review authors explain their selection of the study designs for inclusion in the review? | **For Yes, the review should satisfy ONE of the following:  - explanation for including only RCTs - OR explanation for including only NRSI - OR explanation for including both RCTs and NRSI** |  | YES |  |
| 4. | Did the review authors use a comprehensive literature search strategy? | **For Partial Yes (all the following):  - searched at least 2 databases (relevant to research question) - provided key word and/or search strategy - justified publication restrictions (e.g., language)** | **For Yes, also have (all the flowing):  - searched the reference list/bibliographies of included studies - searched trial/study registries - included/consulted content experts in the field - where relevant, searched for grey literature** | YES |  |
| 5. | Did the review authors perform study selection in duplicate? | **For Yes, either ONE of the following:  - at least two reviewers independently agreed on selection of eligible studies and achieved consensus on which studies to include - OR two reviewers selected a sample of eligible studies and achieved good agreement (at least 80 percent), with the remainder selected by one reviewer.** |  | YES |  |
| 6. | Did the review authors perform data extraction in duplicate? | **For Yes, either ONE of the following:  - at least two reviewers achieved consensus on which data to extract from included studies - OR two reviewers extracted data from a sample of eligible studies and achieved good agreement (at least 80 percent), with the remainder extracted by one reviewer** |  | YES |  |
| 7. | Did the review authors provide a list of excluded studies and justify the exclusions? | **For Partial Yes:  - provided a list of all potentially relevant studies that were read in full-text form but excluded from the review** | **For Yes, must also have:  - justified the exclusion from the review of each potentially relevant study** | YES |  |
| 8. | Did the review authors describe the included studies in adequate detail? | **For Partial Yes:  - described populations - described interventions - described comparators - described outcomes - described research designs** | **For Yes, must also have ALL the following:  - described population in detail - described intervention in detail (including doses where relevant) - described comparator in detail (including doses where relevant) - described study's setting - period for follow-up** | YES |  |
| 9. | Did the review authors use a satisfactory technique for assessing the risk of bias (RoB) in individual studies that were included in the review? | **RCTs For Partial Yes, must have assessed RoB from:  - unconcealed allocation, and - lack of blinding of patients and assessors when assessing outcomes (unnecessary for objective outcomes such as all- cause mortality)** | **RCTs For Yes, must also have assessed RoB from:  - allocation sequence that was not truly random, and - selection of the reported result from among multiple measurements or analyses of a specified outcome** | YES |  |
|  |  | **NRSI For Partial Yes, must have assessed RoB:  - from confounding, and - from selection bias** | **NRSI For Yes, must also have assessed RoB:  - methods used to ascertain exposures and outcomes, and  - selection of the reported result from among multiple measurements or analyses of a specified outcome** |  |  |
| 10. | Did the review authors report on the sources of funding for the studies included in the review? | **For Yes:  - Must have reported on the resources of funding of individual studies included in the review. Note: Reporting that the reviewers looked for this information, but it was not reported by study authors also qualifies** |  | NO |  |
| 11. | If meta-analysis was performed did the review authors use appropriate methods for statistical combination of results? | **RCTs For Yes:  - The authors justified combining the data in a meta-analysis - AND they used an appropriate weighted technique to combine study results and adjusted for heterogeneity** |  | YES |  |
|  |  | **NRSI For Yes:  - The authors justified combining the data in a meta-analysis - AND they used an appropriate weighted technique to combine study results, adjusting for heterogeneity if present - AND they statistically combined effect estimates from NRSI that were adjusted for confounding, rather than combining raw data, or justified combining raw data when adjusted effect estimates were not available - AND they reported separate summary estimates for RCTs and NRSI separately when both were included in the review** |  |  |  |
| 12. | If meta-analysis was performed, did the review authors assess the potential impact of RoB in individual studies on the results of the meta-analysis or other evidence synthesis? | **For Yes:  - Included only low risk of bias RCTs - OR, if the pooled estimate was based on RCTs and/or NRSI at variable RoB, the authors performed analyses to investigate possible impact of RoB on summary estimates of effect** |  | YES |  |
| 13. | Did the review authors account for RoB in individual studies when interpreting/ discussing the results of the review? | **For Yes:  - included only low risk of bias RCTs - OR, if RCTs with moderate or high RoB, or NRSI were included the review provided a discussion of the likely impact of RoB on the results** |  | YES |  |
| 14. | Did the review authors provide a satisfactory explanation for, and discussion of, any heterogeneity observed in the results of the review? | **For Yes:  - There was no significant heterogeneity in the results - OR if heterogeneity was present the authors performed an investigation of sources of any heterogeneity in the results and discussed the impact of this on the results of the review** |  | YES |  |
| 15. | If they performed quantitative synthesis did the review authors carry out an adequate investigation of publication bias (small study bias) and discuss its likely impact on the results of the review? | **For Yes:  - Performed graphical or statistical tests for publication bias and discussed the likelihood and magnitude of impact of publication bias** |  | YES |  |
| 16. | Did the review authors report any potential sources of conflict of interest, including any funding they received for conducting the review? | **For Yes:  - The authors reported no competing interests OR - The authors described their funding sources and how they managed potential conflicts of interest** |  | YES |  |
|  |  |  | **Overall Confidence** | **High** |  |

# Section 12 – Assessment of Sex and Gender Considerations in Included Studies

| **Author** | **Sex/Gender Disaggregated Data** | **Reported Differences** |
| --- | --- | --- |
| **Bailey et al. (2021)** | No gender-disaggregated results are provided. | No gender differences reported. |
| **Berendes et al. (2021)** | No sex/gender-disaggregated data are provided. | No gender differences discussed. |
| **Burns et al. (2016)** | No gender-disaggregated results are provided. | No gender differences found. |
| **Clarke et al. (2022)** | No sex/gender-disaggregated data are provided. | No gender differences mentioned. |
| **Conserve et al. (2017)** | No sex/gender-disaggregated data are provided. | No gender differences reported. |
| **Ilskens et al. (2022)** | No sex/gender-disaggregated data are included. | No gender differences identified. |
| **Jones et al. (2014)** | No sex/gender-disaggregated data are provided. | No gender differences reported. |
| **Kamitani et al. (2024)** | Mention of transgender women included, but no detailed breakdown. | No explicit discussion of gender differences in effectiveness. |
| **Khuwaja and Peck (2022)** | No sex/gender-disaggregated data are provided. | No gender differences discussed. |
| **Knight et al. (2017)** | No sex/gender-disaggregated data are provided. | No gender differences reported. |
| **Manby et al. (2022)** | No gender-disaggregated results are provided. | No gender differences reported. |
| **Nguyen et al. (2019)** | No sex/gender-disaggregated data are provided. | No gender differences discussed. |
| **Ou et al. (2023)** | No gender-disaggregated results are provided. | No gender differences reported. |
| **Palmer et al. (2020)** | No sex/gender-disaggregated data are provided. | No gender differences reported. |
| **Saragih et al. (2024)** | No gender-disaggregated results are provided. | No gender differences reported. |
| **Schnall et al. (2014)** | No gender-disaggregated results are provided. | No gender differences reported. |
| **Sewak et al. (2023)** | No sex/gender-disaggregated data are provided. | No gender differences discussed. |
| **Veronese et al. (2020)** | No gender-disaggregated results are provided. | No gender differences reported. |
| **Xin et al. (2020)** | Focused on men who have sex with men (MSM). No specific breakdown for gender subgroups. | No gender differences discussed. |
| **Du et al. (2025)** | Male-only sample (MSM 100%); no sex/gender disaggregation possible | No sex/gender differences reported because the sample consisted exclusively of MSM |
| **Huang et al. (2025)** | Mixed-gender populations included across primary studies, but no sex/gender-disaggregated results reported in the review | No sex/gender differences reported; the review did not analyze outcomes stratified by sex or gender |
| **Li et al. (2025)** | Included mixed populations (MSM, transgender women, bisexual men, young women, female entertainment workers, adolescents), but none of the included studies reported sex/gender-disaggregated outcomes | No sex/gender differences reported; all results synthesized without gender stratification |
| **Mo et al. (2025)** | Included adolescents and young adults of multiple genders, but none of the RCTs reported sex/gender-disaggregated outcome data | No sex/gender differences reported across HIV knowledge, condom-use self-efficacy, or condom-use outcomes |

# Section 13 – Characteristics of Systematic Reviews

| **Author and year** | **Title** | **Aim of the SR** | **Number of studies included** | **Participants (total number)** | **Year of the most recent study** | **search period or date** | **Place (country -geographic location),** | **Only RCT** | **Only non-RCT** | **RCT and non RCT** | **Number_RCT** |
| --- | --- | --- | --- | --- | --- | --- | --- | --- | --- | --- | --- |
| Bailey 2021 | Interactive digital interventions for prevention of sexually transmitted HIV. | assesses the effectiveness of interactive digital interventions (IDIs) for prevention of sexually transmitted HIV. | 31 | 11293 | 2017 | from 2014 to June 2017 | USA (27/31),Netherlands (1/31), Uganda (1/31), Zambia (1/31) and Sweden (1/31) | Yes | No | No | 31 |
| Berendes 2021 | Sexual health interventions delivered to participants by mobile technology: a systematic review and meta-analysis of randomised controlled trials. | To assess the effectiveness of mobile health interventions delivered to participants for preventing STIs and promoting preventive behaviour. | 22 | 19 551 | 2020 | 1 January 2010–19 February 2020 | 12 trials had been conducted in HICs (USA: n=6; Europe: n=3; Australia: n=3) and 10 in LMICs (Africa: n=7; China: n=2; India: n=1); | Yes | No | No | 22 |
| Burns 2016 | A systematic review of randomised control trials of sexual health interventions delivered by mobile technologies. | The purpose of this systematic review is to update our knowledge of and assess all mHealth interventions for clinic attendance for sexual health and safer sex behaviours (including STI testing, partner notification, condom use number of partners) for all populations, interventions, comparisons, outcomes and studies globally. | 10 | 16773 | 2014 | January 2010 and July 2014 | Irlanda 1/10, Sudafrica 1/10, australia 3/10, USA 3/10, Kanya 2/10 | Yes | No | No | 10 |
| Clarke 2022 | Increasing attendance at pre-booked sexual health consultations: a systematic review. | to identify the range and effectiveness of interventions implemented to improve attendance at pre-booked sexual health consultations | 13 | Not report | 2021 | 1 January 2000 to 1 September 2021. | 5 Australia, 5 America and 3 the United Kingdom. | No | No | Yes | 5 |
| Conserve 2017 | Systematic review of mobile health behavioural interventions to improve uptake of HIV testing for vulnerable and key populations. | This systematic narrative review examined the empirical evidence on the effectiveness of mobile health (mHealth) behavioral interventions designed to increase uptake of HIV testing among vulnerable and key populations. | 7 | Not report | 2015 | January 1, 2005 and August 1, 2015 | India, Australia, UK, Sudafrica, Kenia, USA y China | No | No | Yes | 2 |
| Ilskens 2022 | An Evidence Map on Serious Games in Preventing Sexually Transmitted Infections Among Adolescents: Systematic Review About Outcome Categories Investigated in Primary Studies. | The aim of this systematic review was to identify and systematically summarize the dimensions that have been investigated in primary studies on serious games targeting STI prevention among adolescents. | 18 | Nor report (from incluided table =15286) | 2021 | from 2009 to 2021 | India, Perú, Hong Kong, Puerto Rico, 10 USA, Tanzania, Brazil, Kenya, | No | No | Yes | 8 |
| Jones 2014 | The impact of health education transmitted via social media or text messaging on adolescent and young adult risky sexual behavior: a systematic review of the literature. | to examine the effectiveness of social media and text messaging interventions designed to increase sexually transmitted disease (STD) knowledge, increase screening/testing, decrease risky sexual behaviors, and reduce the incidence of STDs among young adults aged 15 through 24 years. | 11 | not report | 2014 | no especifica | no especifica | No | No | Yes | 5 |
| Kamitani 2024 | A Community Guide Systematic Review: Digital HIV Pre-exposure Prophylaxis Interventions. | to present the characteristics and effectiveness of digital PrEP adherence interventions. | 9 | not report | 2022 | from 2000 to 2022 | 8/9 USA, 1 netherlands | No | No | Yes | 5 |
| Khuwaja 2022 | Increasing HPV Vaccination Rates Using Text Reminders: An Integrative Review of the Literature. | The purpose of this review is to consider text message reminder system efficacy to improve HPV vaccination rates in eligible children. | 7 | not report | 2021 | between 2011 and 2021 | 6 USA y 1 Australia. | No | No | Yes | 4 |
| Knight 2017 | Online interventions to address HIV and other sexually transmitted and blood-borne infections among young gay, bisexual and other men who have sex with men: a systematic review. | To assess the status of published research (e.g. effectiveness; acceptability; differential effects across subgroups) involving online interventions that address HIV/STBBIs among young gbMSM. | 17 | 4669 | 2016 | from inception to November 2016 | 12 USA, Hong Kong, 2 Peru, China, and Thailand | No | No | Yes | 12 |
| Manby 2022 | Effectiveness of eHealth Interventions for HIV Prevention and Management in Sub-Saharan Africa: Systematic Review and Meta-analyses. | To systematically evaluate the effectiveness of eHealth interventions for sexually transmitted HIV prevention in SSA. (Sub‑Saharan Africa) | 25 | 15343 | 2020 | from 2000 to 2020 | all Sudafrica | Yes | No | No | 25 |
| Nguyen 2019 | A Systematic Review of eHealth Interventions Addressing HIV/STI Prevention Among Men Who Have Sex With Men. | The aim of this review was to summarize and appraise the existing eHealth interventions related to HIV/STI prevention among MSM. In addition, this study identified and summarized considerations of eHealth intervention implementation in order to sustain effectiveness over time among MSM populations. | 55 | not report | 2019 | through 1 June 2019 | United States (n = 36 studies) and Asia and Australia regions (n = 13 studies), Canada and Latin America (n=3) Europa (n=3) | No | No | Yes | 37 |
| Ou 2023 | The Effectiveness of mHealth Interventions Targeting Parents and Youth in Human Papillomavirus Vaccination: Systematic Review. | to conduct a systematic review to assess the effectiveness of mHealth interventions on parental intent to vaccinate youth against HPV and youth’s vaccine uptake. | 17 | not report | 2022 | January 2011 and December 2022 | 14 Usa, Netherlands, Australia, and Japan, | No | No | Yes | 12 |
| Palmer 2020 | Targeted client communication via mobile devices for improving sexual and reproductive health. | We assessed the effect of sending targeted messages by mobile devices to young people and adults about their sexual and reproductive health (SRH). Sexually transmitted infections (STIs) and unintended pregnancies are important causes of illness and early death worldwide. | 40 | 26854 | 2019 | enero de 2010 a julio de 2017), se exctulizó en 2019 | All the trials conducted among adolescent populations were carried out in high‐income countries, with the exception of one conducted in Ghana, a lower‐middle‐income country | Yes | No | No | 40 |
| Saragih 2021 | Effects of telehealth interventions for adolescent sexual health: A systematic review and meta-analysis of randomized controlled studies. | This study aimed to explore the meta-effects of telehealth interventions on self-efficacy of using condoms, condom use practices, and sexually transmitted infection testing behaviors among adolescents. | 15 | 5499 | 2021 | 1 January 2002 to 8 May 2021 | 10/15 USA 1 de cada Kenya, Hong Kong, Netherlands, Bolivia, and Palestine. | Yes | No | No | 15 |
| Schnall 2014 | eHealth interventions for HIV prevention in high-risk men who have sex with men: a systematic review. | to examine the use of eHealth interventions for HIV prevention in high-risk MSM. | 13 | not report | 2014 | from January 2000 to April 2014 | 9 USA, and the remaining studies were conducted in Peru (n=1), Australia (n=1), Taiwan (n=1), and Hong Kong (n=1). | No | Yes | No | 8 |
| Sewak 2023 | The effectiveness of digital sexual health interventions for young adults: a systematic literature review (2010-2020). | The aims of this systematic review study are threefold. First, to build on previous reviews (Salam et al., 2016) and reflect the current trends and practices of the safe-sexual health promotion sector when digitizing interventions. | 61 | not report | 2020 | 2010-2020 | USA (n = 32), the UK, Australia and Nigeria, with three studies from each country. Mexico, Sweden and South Africa contributed two studies each. Only one study was found for Argentina(1) y Chile (1), Colombia, Ghana, Hong Kong, Iran, the Netherlands , Portugal, Senegal, South Korea , Spain , Tajikistan, Tanzania and Uganda | No | No | Yes | 40 |
| Veronese 2020 | Using Digital Communication Technology to Increase HIV Testing Among Men Who Have Sex With Men and Transgender Women: Systematic Review and Meta-Analysis. | We undertook a systematic review and meta-analysis to assess the impact of digital communication technology on HIV testing uptake among MSM and transgender women (TW). Subanalyses aimed to identify the features and characteristics of digital interventions associated with greater impact. | 13 | 8875 | 2018 | January 1, 2010, and May 1, 2018 | the majority of studies took place in high-income countries (Hong Kong, n=1; Taiwan, n=1; and United States, n=6), 4 occurred in upper-middle-income countries (China, n=1 and Peru, n=3), and 1 in a low-middle-income country (India) | No | Yes | No | 10 |
| Xin 2020 | The Effectiveness of Electronic Health Interventions for Promoting HIV-Preventive Behaviors Among Men Who Have Sex With Men: Meta-Analysis Based on an Integrative Framework of Design and Implementation Features. | This study aimed to conduct a meta-analysis of the effectiveness of eHealth technology–based interventions for promoting HIV-preventive behaviors among MSM and to determine effectiveness predictors within a framework integrating design and implementation features. | 44 | 27704 | 2019 | 2006-2019 | Over half of the eligible programs (23/44, 52%) were conducted in the United States, and 10 and 8 programs were conducted in Asia and Europe, respectively | No | No | Yes | 44 |
| Du 2025 | Effectiveness of Digital Health Interventions in Promoting the Pre-Exposure Prophylaxis (PrEP) Care Continuum among Men who Have Sex with Men (MSM): A Systematic Review of Randomized Controlled Trials | The aim was to evaluate the effectiveness of digital health interventions in enhancing the PrEP care continuum (PrEP uptake, adherence, attitudes, beliefs) among MSM populations. | 12 | 2662 | 2024 | Up to July 2, 2024 | USA (10/12), China (2/12) | Yes | No | No | 12 |
| Huang 2025 | Technology-Based HIV Prevention Interventions for Men Who Have Sex With Men in China: Systematic Review and Meta-Analysis | The aim was to evaluate the effectiveness of digital HIV prevention interventions targeting MSM in China and to assess effects on HIV testing and condom use, with a meta-analysis. | 24 | Not reported | 2021 | January 2004 to September 2021 | China (mainland), Hong Kong, Taiwan, Macao | No | No | Yes | 13 |
| Li 2025 | Impact of mHealth on Enhancing Pre-Exposure Prophylaxis (PrEP) Adherence and Strengthening the HIV Prevention Continuum: Systematic Review and Meta-Analysis | The aim was to assess the effectiveness of mHealth interventions in improving PrEP adherence and other steps in the HIV prevention continuum. | 16 | 9809 | 2023 | 2010 to 2023 | USA, China, Kenya, South Africa, Brazil | Yes | No | No | 16 |
| Mo 2025 | Use of Behavior Change Techniques in Digital HIV Prevention Programs for Adolescents and Young People: Systematic Review | The aim was to synthesize evidence on behavior change techniques used in digital HIV prevention programs for adolescents and young people. | 34 | Not reported | 2024 | January 2008 to November 2024 | USA; Nigeria; Uganda; Hong Kong; China; Thailand; Cambodia; Germany | Yes | No | No | 34 |
|  |  |  | 514 | 129481 |  |  |  |  |  |  | 410 |

# Section 14 – Participant Characteristics

## Section 14 a. Participant characteristics by age and target population

| **Section 14a – Participant Characteristics** | | | | | | | | |
| --- | --- | --- | --- | --- | --- | --- | --- | --- |
|  | **Population type** | | | | | | |  |
| **Author and year** | **Adolescents(10 to 19)** | **Youth(20 to 29)** | **Adults ( >29)** | **Men who have sex with men** | **LGTBIQ+** | **People with diagnosis of STD ( without HIV)** | **HIV** | **Target Population (TP)** |
| Bailey 2021 | No | Yes | Yes | Yes | Yes | Yes | Yes | Youth, adults, MSM, LGTBIQ+ |
| Berendes 2021 | Yes | Yes | Yes | Yes | Yes | Yes | No | Adolescents , youth, adults , MSM, LGTBIQ+ |
| Burns 2016 | Yes | Yes | Yes | No | No | Yes | No | General population |
| Clarke 2022 | Yes | Yes | Yes | Yes | Yes | Yes | Yes | Adolescents, youth, adults, MSM, LGTBIQ+ |
| Conserve 2017 | Yes | Yes | Yes | Yes | Yes | Yes | Yes | Adolescents, youth, adults, MSM, LGTBIQ+ |
| Ilskens 2022 | Yes | No | No | No | No | No | No | Adolescents |
| Jones 2014 | Yes | Yes | No | No | No | Yes | Yes | Adolescents and young people |
| Kamitani 2024 | Not report | Yes | Yes | Yes | Yes | Not report | No | Young people, adults, MSM, LGTBIQ+ |
| Khuwaja 2022 | Yes | Yes | No | No | No | No | No | Adolescents and young people |
| Knight 2017 | Yes | Yes | No | Yes | Yes | Yes | Yes | Adolescents, youth, MSM, LGTBIQ+ |
| Manby 2022 | Yes | Yes | Yes | No | No | Yes | Yes | General population |
| Nguyen 2019 | No | Yes | Yes | Yes | No | Yes | Yes | Young people, adults, MSM, LGTBIQ+ |
| Ou 2023 | Yes | Yes | Yes | No | No | No | No | General population |
| Palmer 2020 | Yes | Yes | Yes | Yes | Yes | Yes | Yes | Adolescents, young people, adults, MSM, LGTBIQ+ |
| Saragih 2021 | Yes | Yes | No | Yes | No | No | No | Adolescents, young people and MSM |
| Schnall 2014 | No | No | Yes | Yes | No | No | Yes | MSM (adults) |
| Sewak 2023 | No | Yes | Yes | No | No | No | No | Young people and adults |
| Veronese 2020 | No | Yes | Yes | Yes | Yes | No | No | Young people, adults, MSM, LGTBIQ+ |
| Xin 2020 | No | Yes | Yes | Yes | Yes | No | No | Youth, adults, MSM, LGTBIQ+ |
| Du 2025 | No | No | Yes | Yes | No | No | No | MSM aged 18–65; young MSM; sexual minority men |
| Huang 2025 | No | No | Yes | Yes | No | No | No | MSM in China; HIV testing & condom use |
| Li 2025 | No | No | Yes | Yes | No | No | No | Adults at HIV risk; MSM; general adult populations in PrEP programs |
| Mo 2025 | Yes | Yes | No | No | No | No | No | Adolescents and young people in digital HIV prevention programs |

## Section 14 b. Grouping by Target Population and authors of systematic reviews

| **Target Population*** | **Studies** |
| --- | --- |
| **Adolescents** | Berendes S, 2021; Burns K, 2016; Clarke R, 2022; Conserve DF, 2017; Ilskens K, 2022; Jones K, 2014; Khuwaja SS, 2022; Knight R, 2017; Manby L, 2022; Ou L, 2023; Palmer MJ, 2020; Saragih ID, 2021; Mo 2025 |
| **Young people** | Bailey JV, 2021; Berendes S, 2021; Burns K, 2016; Clarke R, 2022; Conserve DF, 2017; Ilskens K, 2022; Jones K, 2014; Kamitani E, 2024; Khuwaja SS, 2022; Knight R, 2017; Manby L, 2022; Nguyen LH, 2019; Ou L, 2023; Palmer MJ, 2020; Saragih ID, 2021; Sewak A, 2023; Veronese V, 2020; Xin M, 2020; Mo 2025 |
| **Adults** | Bailey JV, 2021; Berendes S, 2021; Burns K, 2016; Clarke R, 2022; Conserve DF, 2017; Kamitani E, 2024; Knight R, 2017; Manby L, 2022; Nguyen LH, 2019; Ou L, 2023; Palmer MJ, 2020; Schnall R, 2014; Sewak A, 2023; Veronese V, 2020; Xin M, 2020; Du 2025; Huang 2025; Li 2025 |
| **MSM** | Bailey JV, 2021; Berendes S, 2021; Clarke R, 2022; Conserve DF, 2017; Kamitani E, 2024; Knight R, 2017; Nguyen LH, 2019; Palmer MJ, 2020; Saragih ID, 2021; Schnall R, 2014; Veronese V, 2020; Xin M, 2020; Du 2025; Huang 2025; Li 2025 |
| **LGTBIQ+** | Bailey JV, 2021; Berendes S, 2021; Clarke R, 2022; Conserve DF, 2017; Kamitani E, 2024; Knight R, 2017; Nguyen LH, 2019; Palmer MJ, 2020; Veronese V, 2020; Xin M, 2020 |
| *Adolescents (10-19 years), Young people (20-29 years), Adults (>29 years), MSM: Men who have sex with men, Sexual Diversity Groups (LGTBIQ+) | |

# Section 15 – Characteristics of Digital Interventions

## Section 15a – Characteristics of Digital Interventions about target population, target behavior, and where were implemented.

| **Author and year** | **Adolescent s(population age=or 10 to 19)** | **Youth(20 to 29)** | **Adults ( >29)** | **Men who have sex with men** | **Grupos diversidad sexual (LGTBIQ+)** | **People with diagnosis of STD ( without HIV)** | **Target of behavior (Prevention of STI/HIV)** | **Target Behavior (Promotion of safe sex conducts** | **Target of behavior ( Diagnosis)** | **Target Behavior( treatment-related behaviour.)** | **Environment: schools (where it was implemented)** | **Environment: universities (where it was implemented)** | **Environment:health centers (was implemented)** |
| --- | --- | --- | --- | --- | --- | --- | --- | --- | --- | --- | --- | --- | --- |
| Bailey 2021 | No | Yes | Yes | Yes | Yes | Yes | Yes | Yes | Yes | Yes | Yes | Yes | Yes |
| Berendes 2021 | Yes | Yes | Yes | Yes | Yes | Yes | Yes | Yes | Yes | Yes | Yes | No | Yes |
| Burns 2016 | Yes | Yes | Yes | No | No | Yes | Yes | Yes | Yes | No | No | No | Yes |
| Clarke 2022 | Yes | Yes | Yes | Yes | Yes | Yes | Yes | No | Yes | Yes | No | No | Yes |
| Conserve 2017 | Yes | Yes | Yes | Yes | Yes | Yes | No | No | Yes | No | No | No | Yes |
| Ilskens 2022 | Yes | No | No | No | No | No | Yes | Yes | No | No | No | No | No |
| Jones 2014 | Yes | Yes | No | No | No | Yes | Yes | Yes | Yes | Yes | Yes | Yes | Yes |
| Kamitani 2024 | Not report | Yes | Yes | Yes | Yes | Not report | Yes | Yes | No | Yes | No | No | Yes |
| Khuwaja 2022 | Yes | Yes | No | No | No | No | Yes | Yes | No | Yes | Yes | No | No |
| Knight 2017 | Yes | Yes | No | Yes | Yes | Yes | Yes | Yes | Yes | Yes | Yes | No | No |
| Manby 2022 | Yes | Yes | Yes | No | No | Yes | Yes | Yes | Yes | Yes | Yes | No | No |
| Nguyen 2019 | No | Yes | Yes | Yes | No | Yes | Yes | Yes | Yes | No | No | No | No |
| Ou 2023 | Yes | Yes | Yes | No | No | No | No | No | No | Yes | Yes | No | Yes |
| Palmer 2020 | Yes | Yes | Yes | Yes | Yes | Yes | Yes | Yes | Yes | Yes | Yes | No | Yes |
| Saragih 2021 | Yes | Yes | No | Yes | No | No | Yes | Yes | Yes | No | Yes | Yes | Yes |
| Schnall 2014 | No | No | Yes | Yes | No | No | Yes | Yes | Yes | No | No | No | No |
| Sewak 2023 | No | Yes | Yes | No | No | No | Yes | Yes | No | No | No | No | No |
| Veronese 2020 | No | Yes | Yes | Yes | Yes | No | No | No | Yes | No | No | No | No |
| Xin 2020 | No | Yes | Yes | Yes | Yes | No | No | No | Yes | No | No | No | No |
| Du 2025 | No | No | Yes | Yes | No | No | Yes | No | No | No | No | No | No |
| Huang 2025 | No | No | Yes | Yes | No | No | Yes | Yes | Yes | No | No | No | No |
| Li 2025 | No | No | Yes | Yes | No | No | Yes | No | Yes | No | No | No | No |
| Mo 2025 | Yes | Yes | No | No | No | No | Yes | Yes | Yes | No | Yes | Yes | No |

## Section 15b – Characteristics of Digital Interventions about description about theory used and behavioral outcomes

|  | **Description of framework or theory used** | | | | **Behavioural outcomes** | | | | | | |
| --- | --- | --- | --- | --- | --- | --- | --- | --- | --- | --- | --- |
| **Author and year** | **Explicit framework of theoretical domains of behavior change from the studies included in the SR** | **Description Behavior Change Techniques according to BCTTv1** | **Coding and mapping according to Behavior Change Wheel -BCW -** | **Description according to Theoretical domains framework (TDF)** | **Condom use (internal or external use)** | **Frequency in unprotected sexual intercourse** | **Number of sexual partners** | **STD/HIV testing** | **Uptake of medical male circumcision** | **HIV counselling** | **Get vaccinated against (VPH- HEP A y B)** |
| Bailey 2021 | Yes | No | No | Yes | Yes | Yes | No | Yes | Yes | No | No |
| Berendes 2021 | No | No | No | No | Yes | No | No | Yes | No | No | No |
| Burns 2016 | Yes | Yes | No | No | Yes | Yes | Yes | Yes | No | Yes | No |
| Clarke 2022 | Yes | Yes | No | No | No | No | No | Yes | No | Yes | No |
| Conserve 2017 | No | No | No | No | No | No | No | Yes | No | No | No |
| Ilskens 2022 | No | No | No | No | Yes | Yes | Yes | No | No | No | No |
| Jones 2014 | No | No | No | No | Yes | Yes | Yes | Yes | No | No | No |
| Kamitani 2024 | No | No | No | No | Yes | Yes | No | No | No | No | No |
| Khuwaja 2022 | No | No | No | No | No | No | No | No | No | No | Yes |
| Knight 2017 | Yes | No | No | No | Yes | Yes | Yes | Yes | No | Yes | No |
| Manby 2022 | No | No | No | No | Yes | Yes | No | Yes | Yes | Yes | No |
| Nguyen 2019 | Yes | No | No | No | Yes | Yes | No | Yes | No | Yes | Yes |
| Ou 2023 | Yes | No | No | No | No | No | No | No | No | No | Yes |
| Palmer 2020 | Yes | No | No | No | Yes | Yes | Yes | Yes | Yes | Yes | Yes |
| Saragih 2021 | No | No | No | No | Yes | No | No | Yes | No | No | Yes |
| Schnall 2014 | Yes | No | No | No | Yes | Yes | No | Yes | No | No | No |
| Sewak 2023 | No | No | No | No | Yes | No | No | Yes | No | No | No |
| Veronese 2020 | Yes | No | No | No | No | No | No | Yes | No | No | No |
| Xin 2020 | Yes | No | No | No | Yes | Yes | Yes | Yes | No | No | No |
| Du 2025 | No | No | No | No | No | No | No | Yes | No | No | No |
| Huang 2025 | No | No | No | No | Yes | No | No | Yes | No | No | No |
| Li 2025 | Yes | No | No | No | No | No | No | Yes | No | No | No |
| Mo 2025 | Yes | No | No | No | Yes | No | No | Yes | No | No | Yes |

## Section 15c – Characteristics of Digital Interventions about cognitive outcomes, biological outcomes and, criteria APEASE

|  | **Cognitive outcomes (mediators of prevention)** | | | | **Biological outcomes** | **APEASE (Acceptability, Practicability, Effectiveness, Affordability, Spill-over effects, and Equity)** | | | | | |
| --- | --- | --- | --- | --- | --- | --- | --- | --- | --- | --- | --- |
| **Author and year** | **Self-efficacy** | **STD/HIV related knowledge** | **Attitudes towards condom use** | **PrEP awareness (pre-exposure prophylaxis)** | **HIV or STI acquisition** | **Acceptability** | **Practicability** | **Effectiveness** | **Affordability** | **Spill-over effects** | **Equity** |
| Bailey 2021 | Yes | Yes | Yes | No | Yes | No | No | Yes | No | No | No |
| Berendes 2021 | Yes | Yes | No | No | Yes | No | No | Yes | No | No | No |
| Burns 2016 | No | Yes | No | No | No | Yes | No | Yes | No | No | No |
| Clarke 2022 | Yes | No | No | No | Yes | No | No | Yes | No | No | No |
| Conserve 2017 | No | No | No | No | Yes | No | No | Yes | No | No | No |
| Ilskens 2022 | Yes | Yes | Yes | No | No | Yes | No | Yes | No | No | No |
| Jones 2014 | Yes | Yes | Yes | No | Yes | No | No | Yes | No | No | No |
| Kamitani 2024 | No | No | No | Yes | Yes | Yes | No | Yes | No | No | No |
| Khuwaja 2022 | Yes | No | No | No | No | Yes | No | Yes | No | No | No |
| Knight 2017 | Yes | Yes | Yes | Yes | Yes | Yes | No | Yes | No | No | No |
| Manby 2022 | Yes | Yes | Yes | No | No | No | No | Yes | No | No | No |
| Nguyen 2019 | Yes | Yes | Yes | Yes | No | No | No | Yes | No | No | No |
| Ou 2023 | No | No | No | No | No | Yes | No | Yes | No | No | No |
| Palmer 2020 | Yes | Yes | Yes | Yes | Yes | No | No | Yes | No | No | No |
| Saragih 2021 | Yes | Yes | No | No | No | No | No | Yes | No | No | No |
| Schnall 2014 | No | No | No | No | No | No | No | Yes | No | No | No |
| Sewak 2023 | Yes | Yes | Yes | No | No | No | No | Yes | No | No | No |
| Veronese 2020 | No | No | No | No | No | No | No | Yes | No | No | No |
| Xin 2020 | No | No | No | No | No | No | No | Yes | No | No | No |
| Du 2025 | Not reported | Not reported | Not reported | Yes | Not reported | Yes | Yes | Yes | Not reported | Not reported | Not reported |
| Huang 2025 | Not reported | Not reported | Not reported | Not reported | Not reported | Not reported | Not reported | Not reported | Not reported | Not reported | Not reported |
| Li 2025 | Yes | Yes | Not reported | Yes | Not reported | Not reported | Not reported | Not reported | Not reported | Not reported | Not reported |
| Mo 2025 | Yes | Yes | Yes | Not reported | Yes | Not reported | Not reported | Not reported | Not reported | Not reported | Not reported |

## Section 15d – Characteristics of Digital Interventions about Mode of Delivery (MoD)

Classify how the intervention was implemented, organized into the 15 upper-level categories that describe the characteristics of the delivery mode. These categories capture various aspects of intervention design and implementation from: Marques MM, Carey RN, Norris E, Evans F, Finnerty AN, Hastings J, Jenkins E, Johnston M, West R, Michie S. Delivering Behaviour Change Interventions: Development of a Mode of Delivery Ontology. Wellcome Open Res. 2021 Feb 26;5:125. doi: 10.12688/wellcomeopenres.15906.2. PMID: 33824909; PMCID: PMC7993627.

|  | **Technology delivered** | | | | | | **Digital content type** | | | | | | | | | **Other descriptions** |
| --- | --- | --- | --- | --- | --- | --- | --- | --- | --- | --- | --- | --- | --- | --- | --- | --- |
| **Author and year** | **Mobile device** | **Desktop computer** | **Digital billboard** | **Wearable accessory** | **Digital Environment Object** | **Projection /hologram** | **Audio call/message** | **Video call/ message** | **Text/instant message** | **Email** | **Video Game** | **Audio Broadcast/podcast** | **Website/computer /program/app** | **Ebook** | **Virtual or augmeted reality** | **Used Artificial Intelligence–Based Chatbots for Promoting safe sex or other sexual Behavioral** |
| Bailey 2021 | Yes | No | No | No | No | No | No | No | Yes | No | No | No | No | No | No | No |
| Berendes 2021 | Yes | No | No | No | No | No | No | Yes | Yes | No | No | No | Yes | No | No | No |
| Burns 2016 | Yes | Yes | No | No | No | No | No | Yes | Yes | No | No | No | Yes | No | No | No |
| Clarke 2022 | Yes | No | No | No | No | No | Yes | Yes | Yes | Yes | No | No | Yes | No | No | No |
| Conserve 2017 | Yes | Yes | No | No | No | No | Yes | No | Yes | Yes | No | No | Yes | No | No | No |
| Ilskens 2022 | Yes | Yes | No | No | No | No | No | No | No | No | Yes | No | Yes | No | No | No |
| Jones 2014 | Yes | No | No | No | No | No | No | Yes | Yes | Yes | No | No | Yes | No | No | No |
| Kamitani 2024 | Yes | No | No | No | No | No | Yes | Yes | Yes | Yes | No | No | Yes | No | No | No |
| Khuwaja 2022 | Yes | No | No | No | No | No | Yes | No | Yes | No | No | No | Yes | No | No | No |
| Knight 2017 | Yes | No | No | No | No | No | No | Yes | Yes | Yes | No | No | Yes | No | No | No |
| Manby 2022 | Yes | Yes | No | No | No | No | Yes | Yes | Yes | No | Yes | No | Yes | No | No | No |
| Nguyen 2019 | Yes | Yes | No | No | No | No | No | Yes | Yes | Yes | No | No | Yes | No | Yes | No |
| Ou 2023 | Yes | Yes | No | No | No | No | Yes | Yes | Yes | No | No | No | Yes | Yes | No | No |
| Palmer 2020 | Yes | No | No | No | No | No | Yes | Yes | Yes | Yes | Yes | No | Yes | No | No | No |
| Saragih 2021 | Yes | Yes | No | No | No | No | Yes | Yes | Yes | No | Yes | No | Yes | No | No | No |
| Schnall 2014 | Yes | Yes | No | No | No | No | No | No | Yes | Yes | Yes | No | Yes | No | Yes | No |
| Sewak 2023 | Yes | Yes | No | No | No | No | Yes | Yes | Yes | No | Yes | Yes | Yes | No | No | No |
| Veronese 2020 | Yes | Yes | No | No | No | No | No | Yes | Yes | Yes | No | No | Yes | No | No | No |
| Xin 2020 | Yes | Yes | No | No | No | No | Yes | Yes | Yes | No | No | No | Yes | No | No | No |
| Du 2025 | Yes | No | No | No | No | No | Yes | Yes | Yes | No | No | Yes | No | No | No | No |
| Huang 2025 | Yes | No | No | No | No | No | No | No | No | No | No | No | Yes | No | No | No |
| Li 2025 | Yes | No | No | No | No | No | No | No | Yes | Yes | Yes | No | Yes | No | No | Yes |
| Mo 2025 | Yes | Yes | No | No | No | No | No | No | Yes | No | Yes | No | Yes | No | No | No |

|  | Risk of bias and certainty of evidence | | |
| --- | --- | --- | --- |
| **Author and year** | **Risk of bias tool** | **Meta-analysis** | **Certainty of Evidence (GRADE)** |
| Bailey 2021 | Cochrane risk of bias (ROB) assessment tool | Yes | No |
| Berendes 2021 | Cochrane risk of bias (ROB) assessment tool | Yes | Yes |
| Burns 2016 | Cochrane risk of bias (ROB) assessment tool | No | No |
| Clarke 2022 | The Mixed Methods Appraisal Tool (MMAT) | No | No |
| Conserve 2017 | no reported | No | No |
| Ilskens 2022 |  | No | No |
| Jones 2014 |  | No | No |
| Kamitani 2024 |  | No | No |
| Khuwaja 2022 |  | No | No |
| Knight 2017 | Risk of bias was assessed using the Cochrane risk of bias instrument for randomized controlled trials (RCTs) and the modified Newcastle Ottawa scale for non-randomized studies | No | No |
| Manby 2022 | Cochrane risk of bias (ROB) assessment tool | Yes | No |
| Nguyen 2019 | ICROMS | No | No |
| Ou 2023 | The level of evidence of the included studies was assessed using the modified Melnyk Levels of Evidence [14]. In addition, each study was assessed for its risk of bias and level of certainty in evidence using the Cochrane Grading of Recommendations Assessment, Development, and Evaluation methodology | No | Yes |
| Palmer 2020 | Cochrane risk of bias (ROB) assessment tool | Yes | Yes |
| Saragih 2021 | Cochrane risk-of-bias tool for randomized trials ROB-2 | Yes | No |
| Schnall 2014 | A quality assessment tool for evaluating HIV prevention interventions was created based on the previously published efficacy criteria developed by the Center for Disease Control and Prevention’s HIV/AIDS Prevention Research Branch | No | No |
| Sewak 2023 |  | No | No |
| Veronese 2020 | Quality Assessment Tool for Quantitative Studies | Yes | Yes |
| Xin 2020 |  | Yes | No |
| Du 2025 | RoB 2 | No | Not reported |
| Huang 2025 | Integrated quality criteria for review of multiple study designs (ICROMS) | Yes | Not reported |
| Li 2025 | Not reported | Not reported | Not reported |
| Mo 2025 | JBI Critical Appraisal Tool (Joanna Briggs Institute) | No | Not reported |

## Section 15e – Characteristics of Digital Interventions about distribution of theoretical frameworks used in Interventions use of theory, Behavioral Determinants and Behavior change techniques (BCTs)

| **Author and year** | **Behavior change framework used** | **Framework/ Theory description**  **(if was reported)** | **BCT description** | **TDF description** |
| --- | --- | --- | --- | --- |
| **Bailey 2021** | Yes | The included studies reported using personalized feedback based on user knowledge, motivation, and behavioral skills. Other interventions included interactive texts, virtual peer discussions, decision-making scenarios, and social support visualization. | Not reported | Yes |
| **Berendes 2021** | No | Not specified. | Not reported | Not reported |
| **Burns 2016** | Yes | Theory of Planned Behavior, Precaution Adoption Process Model, social cognitive theory, Information-Motivation-Behavioral Skills (IMB) model of AIDS risk behavior change. | Yes | Not reported |
| **Clarke 2022** | Yes | Based on IMB model of adherence and social action theory. | Yes | Not reported |
| **Conserve 2017** | No | Not specified | Not reported | Not reported |
| **Ilskens 2022** | No | Not specified | Not reported | Not reported |
| **Jones 2014** | No | Not specified | Not reported | Not reported |
| **Kamitani 2024** | No | Not specified | Not reported | Not reported |
| **Khuwaja 2022** | No | Not specified | Not reported | Not reported |
| **Knight 2017** | Yes | Social cognitive theory, Health Belief Model, Integrated Behavioral Model, IMB model. | Not reported | Not reported |
| **Manby 2022** | No | Not specified | Not reported | Not reported |
| **Nguyen 2019** | Yes | IMB model, social cognitive theory, Stages of Change, Health Belief Model, Sexual Health Model, social support theory. | Not reported | Not reported |
| **Ou 2023** | Yes | IMB model, PRECEDE-PROCEED model, Health Belief Model, Theory of Planned Behavior, social cognitive theory, Theory of Reasoned Action. | Not reported | Not reported |
| **Palmer 2020** | Yes | Social support theory, IMB model, social cognitive theory. | Not reported | Not reported |
| **Saragih 2021** | No | Telehealth interventions were based on self-efficacy theory and behavioral skills development. | Not reported | Not reported |
| **Schnall 2014** | Yes | IMB model, Health Belief Model, Stages of Change, social learning theory, social cognition and developmental theory, Sexual Health Model. | Not reported | Not reported |
| **Sewak 2023** | No | Not specified. | Not reported | Not reported |
| **Veronese 2020** | Yes | Self-determination theory principles and integrated behavioral model, Health Belief Model, social learning theory, IMB model, natural helping, empowerment education, social cognitive theory, integrative model of behavior change, diffusions of innovation theory, social normative theory. | Not reported | Not reported |
| **Xin 2020** | Yes | IMB model. The intervention effects on unprotected anal intercourse (UAI) and HIV testing appeared to be comparable regardless of whether any theory was used. | Not reported | Not reported |
| **Du 2025** | No | Not reported | Not reported | Not reported |
| **Huang 2025** | No | Not reported | Not reported | Not reported |
| **Li 2025** | Yes | Some included studies used behavioral theories (e.g., IMB model, Social Cognitive Theory), but no single framework was applied at the SR level. | Not reported | Not reported |
| **Mo 2025** | Yes | Multiple behavior change theories used in included interventions (e.g., Social Cognitive Theory, Health Belief Model, Theory of Planned Behavior). Review focuses on identifying and coding BCTs across studies. | Yes | Not reported |

Summary of theoretical frameworks and behavioral models applied across the 23 systematic reviews of DBCIs for STI/HIV prevention. Columns indicate whether a behavior change framework was used, its description, and whether Behavior Change Techniques (BCTs) or Theoretical Domains Framework (TDF) constructs were reported. Note: Most reviews did not systematically apply or report behavioral frameworks.

| Distribution of Theoretical Frameworks Used in Interventions | | |
| --- | --- | --- |
| **Theoretical Framework** | **Frequency** | **Percentage** |
| Information-Motivation-Behavioral Skills Model (IMB) | 10 | 14.3% |
| Health Belief Model | 7 | 10.0% |
| Social Cognitive Theory | 7 | 10.0% |
| Theory of Planned Behaviour | 6 | 8.6% |
| Social Learning Theory | 4 | 5.7% |
| Stages of Change Theory | 3 | 4.3% |
| Integrated Behavioural Model | 3 | 4.3% |
| Self-determination Theory | 2 | 2.9% |
| Diffusion of Innovation Theory | 2 | 2.9% |
| Natural Helping | 2 | 2.9% |
| Empowerment Education | 2 | 2.9% |
| Protection-Motivation Theory | 2 | 2.9% |
| Theory of Reasoned Action | 2 | 2.9% |
| Social Normative Theory | 1 | 1.4% |
| Weinstein’s Precaution Adoption Process Model | 2 | 2.9% |
| Bandura’s Concept of Self-efficacy | 2 | 2.9% |
| PRECEDE-PROCEED Model | 1 | 1.4% |
| Sexual Health Model | 2 | 2.9% |
| Social Support Theory | 2 | 2.9% |
| Community Mobilization Model | 1 | 1.4% |
| Popular Opinion Leader Model | 1 | 1.4% |
| Dual Processing Cognitive Emotional Decision Making Framework | 1 | 1.4% |
| Prospect Theory | 1 | 1.4% |
| Common Ingroup Identity Model | 1 | 1.4% |
| Social Identity Theory | 1 | 1.4% |
| Model of Culture-Centric Narratives in Health Promotion | 1 | 1.4% |
| Model of Adherence and Social Action Theory (SAT) | 1 | 1.4% |
| Self-efficacy Theory | 1 | 1.4% |
|  |  |  |
|  | 71 | 1,00 |

# Section 16 – Critical assessments based on AMSTAR 2

| **Author-Year/Criteria** | 1 | 2 | 3 | 4 | 5 | 6 | 7 | 8 | 9 | 10 | 11 | 12 | 13 | 14 | 15 | 16 | **Rating Overall Confidence in the results of the review** |
| --- | --- | --- | --- | --- | --- | --- | --- | --- | --- | --- | --- | --- | --- | --- | --- | --- | --- |
| **Bailey 2021** | YES | YES | YES | YES | YES | YES | YES | YES | YES | NO | YES | YES | YES | YES | YES | YES | **High** |
| **Berendes 2021** | YES | YES | YES | YES | YES | YES | YES | YES | YES | NO | YES | YES | YES | YES | YES | YES | **High** |
| **Burns 2016** | YES | YES | YES | YES | YES | YES | YES | NO | YES | NO | NO META-ANALYSIS | NO META-ANALYSIS | YES | YES | NO META-ANALYSIS | YES | **Moderate** |
| **Clarke 2022** | YES | YES | YES | YES | NO | YES | NO | YES | PARTIAL YES | NO | NO META-ANALYSIS | NO META-ANALYSIS | NO | YES | NO META-ANALYSIS | YES | **Critically low** |
| **Conserve 2017** | NO | PARTIAL YES | YES | YES | YES | YES | NO | YES | YES | NO | NO META-ANALYSIS | NO META-ANALYSIS | YES | NO | NO META-ANALYSIS | YES | **Low** |
| **Ilskens 2022** | YES | NO | YES | PARTIAL YES | NO | YES | NO | YES | NO | NO | NO META-ANALYSIS | NO META-ANALYSIS | NO | YES | NO META-ANALYSIS | NO | **Critically low** |
| **Jones 2014** | YES | NO | YES | YES | NO | NO | NO | YES | NO | NO | NO META-ANALYSIS | NO META-ANALYSIS | NO | NO | NO | NO | **Critically low** |
| **Kamitani 2024** | NO | NO | YES | YES | YES | YES | NO | YES | NO | NO | NO META-ANALYSIS | NO META-ANALYSIS | NO | NO | NO META-ANALYSIS | YES | **Critically low** |
| **Khuwaja 2022** | YES | NO | YES | NO | NO | NO | NO | YES | NO | NO | NO META-ANALYSIS | NO META-ANALYSIS | NO | NO | NO META-ANALYSIS | NO | **Critically low** |
| **Knight 2017** | YES | YES | YES | YES | NO | YES | YES | YES | YES | NO | NO META-ANALYSIS | NO META-ANALYSIS | YES | YES | NO META-ANALYSIS | YES | **Moderate** |
| **Manby 2022** | YES | YES | YES | YES | YES | YES | NO | YES | YES | NO | YES | YES | YES | YES | YES | YES | **High** |
| **Nguyen 2019** | YES | NO | YES | YES | YES | YES | NO | PARTIAL YES | YES | NO | NO META-ANALYSIS | NO META-ANALYSIS | YES | NO | NO META-ANALYSIS | YES | **Critically low** |
| **Ou 2023** | NO | PARTIAL YES | YES | YES | YES | YES | NO | YES | YES | NO | NO META-ANALYSIS | NO META-ANALYSIS | NO | NO | NO META-ANALYSIS | NO | **Critically low** |
| **Palmer 2020** | YES | YES | YES | YES | YES | YES | YES | YES | YES | YES | YES | YES | YES | YES | YES | YES | **High** |
| **Saragih 2021** | YES | YES | YES | PARTIAL YES | YES | YES | NO | YES | YES | NO | YES | YES | YES | YES | YES | YES | **Low** |
| **Schnall 2014** | NO | NO | YES | PARTIAL YES | YES | YES | PARTIAL YES | PARTIAL YES | PARTIAL YES | NO | NO META-ANALYSIS | NO META-ANALYSIS | NO | NO | NO | NO | **Critically low** |
| **Sewak 2023** | YES | NO | YES | YES | YES | YES | PARTIAL YES | PARTIAL YES | NO | NO | NO META-ANALYSIS | NO META-ANALYSIS | NO | NO | NO META-ANALYSIS | YES | **Critically low** |
| **Veronese 2020** | NO | PARTIAL YES | YES | YES | NO | NO | PARTIAL YES | YES | YES | NO | YES | YES | YES | YES | YES | YES | **Moderate** |
| **Xin 2020** | YES | NO | YES | YES | YES | NO | PARTIAL YES | YES | PARTIAL YES | NO | YES | YES | YES | YES | YES | YES | **Low** |
| **Du 2025** | YES | YES | YES | PARTIAL YES | YES | YES | YES | YES | YES | NO | NO META-ANALYSIS | NO META-ANALYSIS | YES | YES | NO META-ANALYSIS | YES | **Low** |
| **Huang 2025** | YES | YES | YES | PARTIAL YES | YES | YES | YES | YES | NO | NO | YES | YES | YES | YES | YES | YES | **Critically low** |
| **Li 2025** | YES | YES | YES | YES | YES | YES | YES | YES | YES | YES | YES | YES | YES | YES | YES | YES | **High** |
| **Mo 2025** | YES | NO | YES | PARTIAL YES | YES | YES | NO | YES | PARTIAL YES | YES | NO META-ANALYSIS | NO META-ANALYSIS | NO | YES | NO META-ANALYSIS | YES | **Critically low** |
| **Rating overall confidence in the results of the review** | **High** | Up to one non-critical weakness: the SR provides an accurate and comprehensive summary of the results of the available studies that address the topic of interest. | | | | | | | | | | | | | | | |
|  | **Moderate** | More than one non-critical weakness*: the SR has more than one weakness but no critical flaws. It may provide an accurate summary of the results of the available studies included in the review. | | | | | | | | | | | | | | | |
|  | **Low** | One critical flaw with or without non-critical weaknesses: the review has a critical flaw and may not provide an accurate, comprehensive summary of the available studies that address the topic of interest. | | | | | | | | | | | | | | | |
|  | **Critically low** | More than one critical flaw with or without non-critical weaknesses: the review has more than one critical flaw and should not be relied on to provide an accurate, comprehensive summary of the available studies. | | | | | | | | | | | | | | | |
|  |  | *Multiple non-critical weaknesses may diminish confidence in the review, and it may be appropriate to move the overall appraisal down from moderate to low confidence. | | | | | | | | | | | | | | | |

Summary of AMSTAR-2 critical appraisal results for the 23 SRs included in this overview. Reviews were classified as high, moderate, low, or critically low confidence depending on the presence of critical and non-critical weaknesses.

*Note:* Below is a brief summary of the 16 AMSTAR-2 items, with critical domains highlighted in bold: 1) clear definition of the research question and inclusion criteria (PICO), **2) protocol registered before starting the review**, 3) justification for included study designs, **4) adequate literature search strategy**, 5) study selection conducted in duplicate, 6) data extraction conducted in duplicate, **7) clear justification for excluded studies**, 8) detailed description of included studies, **9) appropriate assessment of risk of bias in individual studies**, 10) adequate reporting of funding sources, **11) appropriate use of meta-analytical methods**, 12) assessment of the impact of risk of bias on results, **13) interpretation of the risk of bias in discussing results**, 14) discussion of heterogeneity in results, **15) assessment of potential publication bias**, and 16) reporting of conflicts of interest by review authors.

| **AMSTAR 2** Shea BJ, Reeves BC, Wells G, Thuku M, Hamel C, Moran J, Moher D, Tugwell P, Welch V, Kristjansson E, Henry DA. AMSTAR 2: a critical appraisal tool for systematic reviews that include randomised or non-randomised studies of healthcare interventions, or both. BMJ. 2017 Sep 21;358:j4008. | |
| --- | --- |
|  | |
| **Rating Overall Confidence in the results of the review** | **AMSTAR 2  CRITICAL DOMAINS** |
| **High** No or one non-critical weakness: the systematic review provides an accurate and comprehensive summary of the results of the available studies that address the question of interest  **Moderate** More than one non-critical weakness*: the systematic review has more than one weakness but no critical flaws. It may provide an accurate summary of the results of the available studies that were included in the review  **Low** One critical flaw with or without non-critical weaknesses: the review has a critical flaw and may not provide an accurate and comprehensive summary of the available studies that address the question of interest  **Critically low** More than one critical flaw with or without non-critical weaknesses: the review has more than one critical flaw and should not be relied on to provide an accurate and comprehensive summary of the available studies *Multiple non-critical weaknesses may diminish confidence in the review and it may be appropriate to move the overall appraisal down from moderate to low confidence | **2.** Protocol registered before commencement of the review   **4.** Adequacy of the literature search   **7.** Justification for excluding individual studies   **9.** Risk of bias from individual studies being included in the review   **11.** Appropriateness of meta-analytical methods   **13.** Consideration of risk of bias when interpreting the results of the review  **15.** Assessment of presence and likely impact of publication bias |

## Section 16a – Supplementary Material – AMSTAR-2 Quality Assessment of the Four Included Reviews (Du et al., 2025, Huang et al., 2025, Li et al., 2025, and Mo et al., 2025)

|  |  |  | **Author Year** | **Du 2025** |  | **Huang 2025** |  | **Li 2025** |  | **Mo 2025** |  |
| --- | --- | --- | --- | --- | --- | --- | --- | --- | --- | --- | --- |
| **Criteria** | **Question** | **Description** | **Description** | **Vote** | **Quotes** | **Vote** | **Quotes** | **Vote** | **Quotes** | **Vote** | **Quotes** |
| 1. | Did the research questions and inclusion criteria for the review include the components of PICO? | **For yes:   - Population  - Intervention  - Comparator group  - Outcome** | Optionnal (recommended)  - Timeframe for follow-up | YES |  | YES |  | YES |  | YES |  |
| 2. | Did the report of the review contain an explicit statement that the review methods were established prior to the conduct of the review and did the report justify any significant deviations from the protocol? | **For Partial Yes: The authors state that they had written protocol or guide that included ALL the following:   - review question(s)  - a search strategy  - inclusion/exclusion criteria  - a risk of bias assessment** | **For Yes: As for partial yes, plus the protocol should be registered and should also have specfied:  - a meta-analysis/synthesis plan, if appropiate, and - a plan for investigating causes of heterogenity - justification for any deviations from the protocol** | YES |  | YES |  | YES |  | NO |  |
| 3. | Did the review authors explain their selection of the study designs for inclusion in the review? | **For Yes, the review should satisfy ONE of the following:  - explanation for including only RCTs - OR explanation for including only NRSI - OR explanation for including both RCTs and NRSI** |  | YES |  | YES |  | YES |  | YES |  |
| 4. | Did the review authors use a comprehensive literature search strategy? | **For Partial Yes (all the following):  - searched at least 2 databases (relevant to research question) - provided key word and/or search strategy - justified publication restrictions (e.g. language)** | **For Yes, also have (all the flowing):  - searched the reference list/bibliographies of included studies - searched trial/study registries - included/consulted content experts in the field - where relevant, searched for grey literature** | PARTIAL YES |  | PARTIAL YES |  | YES |  | PARTIAL YES |  |
| 5. | Did the review authors perform study selection in duplicate? | **For Yes, either ONE of the following:  - at least two reviewers independently agreed on selection of elegible studies and achieved consensus on which studies to include - OR two reviewers selected a sample of eligible studies and achieved good agreement (at least 80 percent), with the remainder selected by one reviewer.** |  | YES |  | YES |  | YES |  | YES |  |
| 6. | Did the review authors perform data extraction in duplicate? | **For Yes, either ONE of the following:  - at least two reviewers achieved consensus on which data to extract from included studies - OR two reviewers extracted data from a sample of eligible studies and achieved good agreement (at least 80 percent), with the remainder extracted by one reviewer** |  | YES |  | YES |  | YES |  | YES |  |
| 7. | Did the review authors provide a list of excluded studies and justify the exclusions? | **For Partial Yes:  - provided a list of all potentially relevant studies that were read in full-text form but excluded from the review** | **For Yes, must also have:  - justified the exclusion from the review of each potentially relevant study** | YES |  | YES |  | YES |  | NO |  |
| 8. | Did the review authors describe the included studies in adequate detail? | **For Partial Yes:  - described populations - described interventions - described comparators - described outcomes - described research designs** | **For Yes, must also have ALL the following:  - described population in detail - described intervention in detail (including doses where relevant) - described comparator in detail (including doses where relevant) - described study's setting - timeframe for follow-up** | YES |  | YES |  | YES |  | YES |  |
| 9. | Did the review authors use a satisfactory technique for assessing the risk of bias (RoB) in individual studies that were included in the review? | **RCTs For Partial Yes, must have assessed RoB from:  - unconcealed allocation, and - lack of blinding of patients and assessors when assessing outcomes (unnecessary for objective outcomes such as all- cause mortality)** | **RCTs For Yes, must also have assessed RoB from:  - allocation sequence that was not truly random, and - selection of the reported result from among multiple measurements or analyses of a specified outcome** | YES |  | NO |  | YES |  | PARTIAL YES |  |
|  |  | **NRSI For Partial Yes, must have assessed RoB:  - from confounding, and - from selection bias** | **NRSI For Yes, must also have assessed RoB:  - methods used to ascertain exposures and outcomes, and  - selection of the reported result from among multiple measurements or analyses of a specified outcome** |  |  |  |  |  |  |  |  |
| 10. | Did the review authors report on the sources of funding for the studies included in the review? | **For Yes:  - Must have reported on the resources of funding of individual studies included in the review. Note: Reporting that the reviewers looked for this information but it was not reported by study authors also qualifies** |  | NO |  | NO |  | YES |  | YES |  |
| 11. | If meta-analysis was performed did the review authors use appropriate methods for statistical combination of results? | **RCTs For Yes:  - The authors justified combining the data in a meta-analysis - AND they used an appropriate weighted technique to combine study results and adjusted for heterogeneity** |  | NO META-ANALYSIS CONDUCTED |  | YES |  | YES |  | NO META-ANALYSIS CONDUCTED |  |
|  |  | **NRSI For Yes:  - The authors justified combining the data in a meta-analysis - AND they used an appropriate weighted technique to combine study results, adjusting for heterogeneity if present - AND they statistically combined effect estimates from NRSI that were adjusted for confounding, rather than combining raw data, or justified combining raw data when adjusted effect estimates were not available - AND they reported separate summary estimates for RCTs and NRSI separately when both were included in the review** |  |  |  |  |  |  |  |  |  |
| 12. | If meta-analysis was performed, did the review authors assess the potential impact of RoB in individual studies on the results of the meta-analysis or other evidence synthesis? | **For Yes:  - Included only low risk of bias RCTs - OR, if the pooled estimate was based on RCTs and/or NRSI at variable RoB, the authors performed analyses to investigate possible impact of RoB on summary estimates of effect** |  | NO META-ANALYSIS CONDUCTED |  | YES |  | YES |  | NO META-ANALYSIS CONDUCTED |  |
| 13. | Did the review authors account for RoB in individual studies when interpreting/ discussing the results of the review? | **For Yes:  - included only low risk of bias RCTs - OR, if RCTs with moderate or high RoB, or NRSI were included the review provided a discussion of the likely impact of RoB on the results** |  | YES |  | YES |  | YES |  | NO |  |
| 14. | Did the review authors provide a satisfactory explanation for, and discussion of, any heterogeneity observed in the results of the review? | **For Yes:  - There was no significant heterogeneity in the results - OR if heterogeneity was present the authors performed an investigation of sources of any heterogeneity in the results and discussed the impact of this on the results of the review** |  | NO |  | YES |  | YES |  | YES |  |
| 15. | If they performed quantitative synthesis did the review authors carry out an adequate investigation of publication bias (small study bias) and discuss its likely impact on the results of the review? | **For Yes:  - Performed graphical or statistical tests for publication bias and discussed the likelihood and magnitude of impact of publication bias** |  | NO META-ANALYSIS CONDUCTED |  | YES |  | YES |  | NO META-ANALYSIS CONDUCTED |  |
| 16. | Did the review authors report any potential sources of conflict of interest, including any funding they received for conducting the review? | **For Yes:  - The authors reported no competing interests OR - The authors described their funding sources and how they managed potential conflicts of interest** |  | YES |  | YES |  | YES |  | YES |  |
|  |  |  | **Overall Confidence** | **Low** |  | **Critically low** |  | **High** |  | **Critically low** |  |

# Section 17 – Overlap in primary studies included in reviews

Two versions of the GROOVE framework figure are provided to support accessibility and to accommodate different publication formats:

GROOVE Framework – Color Version

This version uses the full color palette of the GROOVE design and is intended for digital viewing, presentations, and contexts where color enhances interpretability.

GROOVE Framework – Greyscale Version

A greyscale version is included to ensure compatibility with print formats and journals that require monochrome figures.

Importantly, this version also enhances accessibility for readers with color vision deficiencies, as it does not rely on color contrasts for interpretation.

Both versions retain identical structure, labels, and visual hierarchy to ensure consistent understanding across formats.


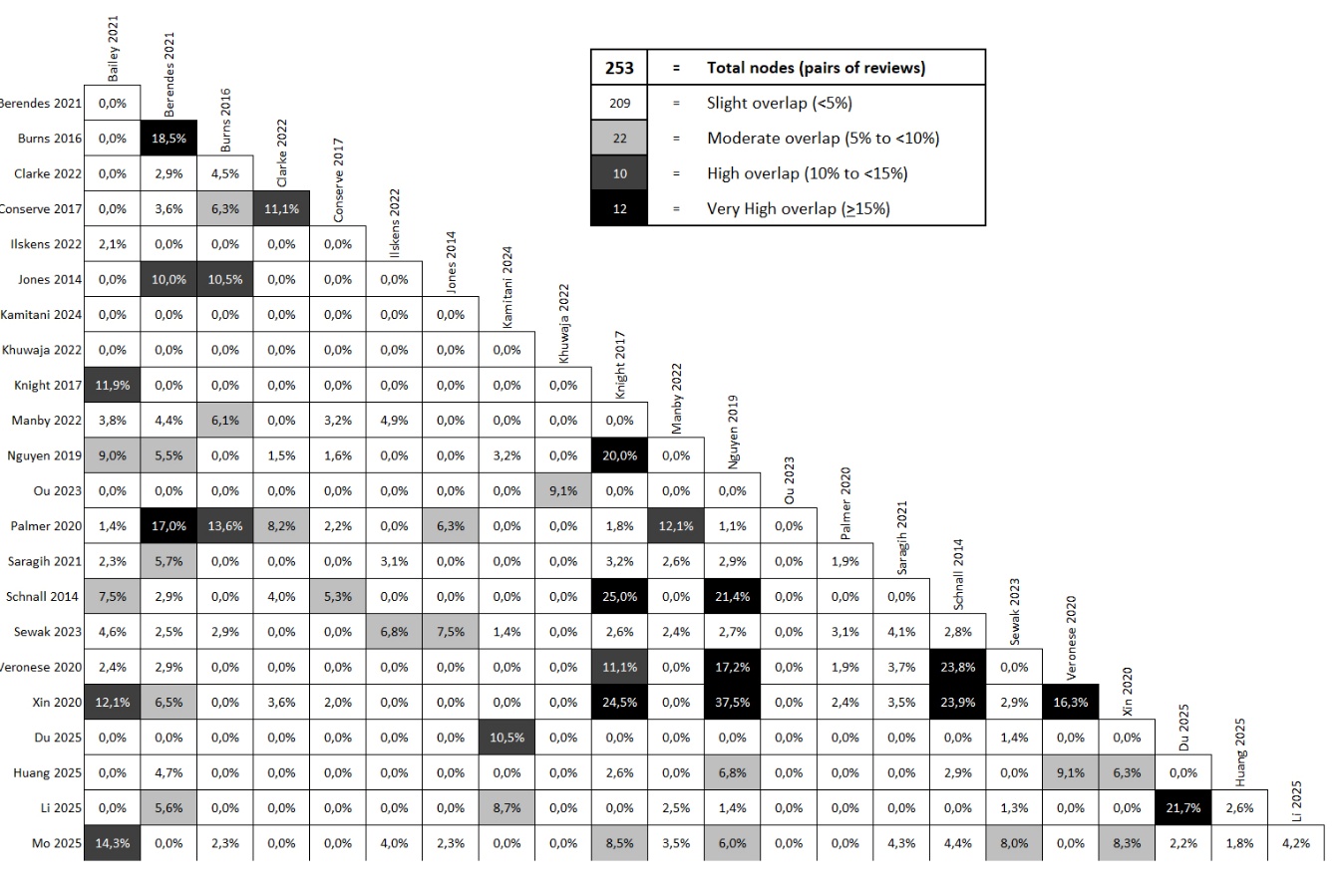


**
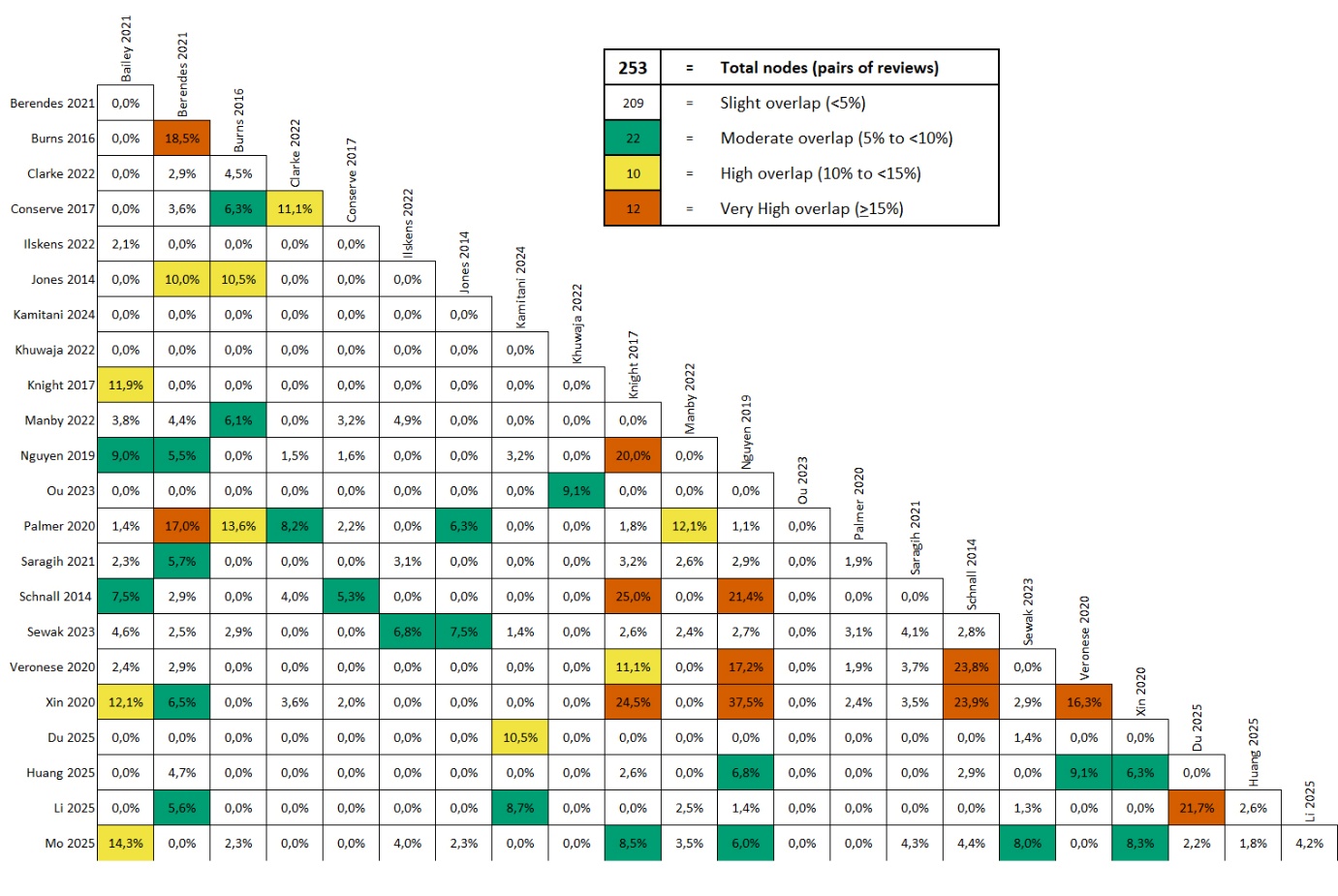
**

Visualization of the degree of overlap in primary studies across the 23 SRs included in this overview, generated using the GROOVE (Graphical Representation of Overlap for Overviews) tool. Color shading represents the degree of overlap: light green (<5%, slight), dark green (5–10%, moderate), yellow (10–15%, high), orange (≥15%, very high). Note: Most review pairs showed minimal overlap (<5%)

# Section 18. Matrix of evidence

| **Primary Studies** | **Systematic Reviews** | | | | | | | | | | | | | | | | | | | | | | |
| --- | --- | --- | --- | --- | --- | --- | --- | --- | --- | --- | --- | --- | --- | --- | --- | --- | --- | --- | --- | --- | --- | --- | --- |
| **Author, year** | **Bailey 2021** | **Berendes 2021** | **Burns 2016** | **Clarke 2022** | **Conserve 2017** | **Ilskens 2022** | **Jones 2014** | **Kamitani 2024** | **Khuwaja 2022** | **Knight 2017** | **Manby 2022** | **Nguyen 2019** | **Ou 2023** | **Palmer 2020** | **Saragih 2021** | **Schnall 2014** | **Sewak 2023** | **Veronese 2020** | **Xin 2020** | **Du 2025** | **Huang 2025** | **Li 2025** | **Mo 2025** |
| Bauermeister 2015 | 1 |  |  |  |  |  |  |  |  | 1 |  | 1 |  |  | 1 |  |  | 1 | 1 |  |  |  |  |
| Billings 2015 | 1 |  |  |  |  |  |  |  |  |  |  |  |  |  |  |  |  |  |  |  |  |  |  |
| Bowen 2007 | 1 |  |  |  |  |  |  |  |  | 1 |  | 1 |  |  |  |  |  |  |  |  |  |  |  |
| Bull 2009 | 1 |  |  |  |  |  |  |  |  |  |  |  |  |  |  |  |  |  |  |  |  |  | 1 |
| Calderon 2013 | 1 |  |  |  |  |  |  |  |  |  |  |  |  |  |  |  |  |  |  |  |  |  |  |
| Carpenter 2010 | 1 |  |  |  |  |  |  |  |  | 1 |  | 1 |  |  |  | 1 |  |  | 1 |  |  |  |  |
| Christensen 2013 | 1 |  |  |  |  |  |  |  |  | 1 |  | 1 |  |  |  | 1 | 1 |  | 1 |  |  |  | 1 |
| Davidovich 2006 | 1 |  |  |  |  |  |  |  |  |  |  |  |  |  |  |  |  |  | 1 |  |  |  |  |
| Di Noia 2004 | 1 |  |  |  |  |  |  |  |  |  |  |  |  |  |  |  |  |  |  |  |  |  |  |
| Evans 2000 | 1 |  |  |  |  |  |  |  |  |  |  |  |  |  |  |  |  |  |  |  |  |  |  |
| Festinger 2016 | 1 |  |  |  |  |  |  |  |  |  |  |  |  |  |  |  |  |  |  |  |  |  |  |
| Fiellin 2017 | 1 |  |  |  |  | 1 |  |  |  |  |  |  |  |  |  |  | 1 |  |  |  |  |  | 1 |
| Gilbert 2008 | 1 |  |  |  |  |  |  |  |  |  |  |  |  |  |  |  |  |  |  |  |  |  |  |
| Hightow-Weidman 2012 | 1 |  |  |  |  |  |  |  |  | 1 |  |  |  |  |  |  |  |  | 1 |  |  |  | 1 |
| Ito 2008 | 1 |  |  |  |  |  |  |  |  |  |  |  |  |  |  |  |  |  |  |  |  |  |  |
| Jenkins 2000 | 1 |  |  |  |  |  |  |  |  |  |  |  |  |  |  |  |  |  |  |  |  |  |  |
| Kiene 2006 | 1 |  |  |  |  |  |  |  |  |  |  |  |  |  |  |  |  |  |  |  |  |  |  |
| Klein 2013 | 1 |  |  |  |  |  |  |  |  |  |  |  |  |  |  |  |  |  |  |  |  |  | 1 |
| Kurth 2014 | 1 |  |  |  |  |  |  |  |  |  |  |  |  |  |  |  |  |  |  |  |  |  |  |
| Leiby 2016 | 1 |  |  |  |  |  |  |  |  |  | 1 |  |  | 1 |  |  |  |  |  |  |  |  |  |
| Marsch 2011 | 1 |  |  |  |  |  |  |  |  |  |  |  |  |  |  |  |  |  |  |  |  |  | 1 |
| Marsch 2015 | 1 |  |  |  |  |  |  |  |  |  |  |  |  |  |  |  | 1 |  |  |  |  |  | 1 |
| McKinstry 2017 | 1 |  |  |  |  |  |  |  |  |  |  |  |  |  |  |  |  |  |  |  |  |  |  |
| Merchant 2011 | 1 |  |  |  |  |  |  |  |  |  |  |  |  |  |  |  |  |  |  |  |  |  |  |
| Milam 2016 | 1 |  |  |  |  |  |  |  |  |  |  | 1 |  |  |  |  |  |  |  |  |  |  |  |
| Perry 1991 | 1 |  |  |  |  |  |  |  |  |  |  |  |  |  |  |  |  |  |  |  |  |  |  |
| Read 2006 | 1 |  |  |  |  |  |  |  |  |  |  |  |  |  |  |  |  |  | 1 |  |  |  |  |
| Rosser 2010 | 1 |  |  |  |  |  |  |  |  |  |  | 1 |  |  |  | 1 | 1 |  | 1 |  |  |  |  |
| Schonnesson 2016 | 1 |  |  |  |  |  |  |  |  |  |  | 1 |  |  |  |  |  |  | 1 |  |  |  |  |
| Ybarra 2013 | 1 |  |  |  |  |  |  |  |  |  | 1 |  |  |  |  |  |  |  |  |  |  |  | 1 |
| de Tolly 2012 |  | 1 | 1 |  | 1 |  |  |  |  |  | 1 |  |  | 1 |  |  |  |  |  |  |  |  |  |
| Delamere 2006 |  | 1 | 1 |  |  |  |  |  |  |  |  |  |  | 1 |  |  |  |  |  |  |  |  |  |
| Downing 2013 |  | 1 | 1 | 1 |  |  |  |  |  |  |  |  |  | 1 |  |  |  |  |  |  |  |  |  |
| Free 2016 |  | 1 |  |  |  |  |  |  |  |  |  |  |  |  |  |  |  |  |  |  |  |  |  |
| Gold 2011 |  | 1 | 1 |  |  |  | 1 |  |  |  |  |  |  | 1 |  |  | 1 |  |  |  |  |  |  |
| Govender 2019 |  | 1 |  |  |  |  |  |  |  |  | 1 |  |  |  |  |  |  |  |  |  |  | 1 |  |
| Kelvin 2019 |  | 1 |  |  |  |  |  |  |  |  |  |  |  |  |  |  |  |  |  |  |  |  |  |
| Kelvin 2019 |  | 1 |  |  |  |  |  |  |  |  |  |  |  |  |  |  |  |  |  |  |  |  |  |
| Lim 2012 |  | 1 |  |  |  |  | 1 |  |  |  |  |  |  | 1 |  |  | 1 |  |  |  |  |  |  |
| Mimiaga 2017 |  | 1 |  |  |  |  |  |  |  |  |  | 1 |  |  |  |  |  |  | 1 |  |  |  |  |
| Mugo 2016 |  | 1 |  |  |  |  |  |  |  |  |  |  |  | 1 |  |  |  |  |  |  |  |  |  |
| Nielsen 2021 |  | 1 |  |  |  |  |  |  |  |  |  |  |  |  |  |  |  |  |  |  |  |  |  |
| Parkes-Ratanshi 2018 |  | 1 |  |  |  |  |  |  |  |  |  |  |  |  |  |  |  |  |  |  |  |  |  |
| Reback 2019 |  | 1 |  |  |  |  |  |  |  |  |  | 1 |  |  |  |  |  |  | 1 |  |  |  |  |
| Rokicki 2017 |  | 1 |  |  |  |  |  |  |  |  |  |  |  | 1 |  |  |  |  |  |  |  |  |  |
| Suffoletto 2013 |  | 1 | 1 |  |  |  | 1 |  |  |  |  |  |  | 1 |  |  |  |  |  |  |  |  |  |
| Tang 2018 |  | 1 |  |  |  |  |  |  |  |  |  |  |  |  |  |  |  |  | 1 |  | 1 |  |  |
| Trent 2019 |  | 1 |  |  |  |  |  |  |  |  |  |  |  |  |  |  |  |  |  |  |  |  |  |
| Ybarra 2017 |  | 1 |  |  |  |  |  |  |  |  |  |  |  | 1 | 1 |  |  |  | 1 |  |  |  |  |
| Young 2013 |  | 1 |  |  |  |  |  |  |  |  |  | 1 |  |  |  | 1 |  | 1 |  |  |  |  |  |
| Zhu X 2019 |  | 1 |  |  |  |  |  |  |  |  |  | 1 |  |  |  |  |  |  |  |  | 1 | 1 |  |
| Jones 2013 |  |  | 1 |  |  |  |  |  |  |  |  |  |  |  |  |  | 1 |  |  |  |  |  | 1 |
| Lim 2010 |  |  | 1 |  |  |  |  |  |  |  |  |  |  |  |  |  |  |  |  |  |  |  |  |
| Odeny 2012 |  |  | 1 |  |  |  |  |  |  |  |  |  |  | 1 |  |  |  |  |  |  |  |  |  |
| Odeny 2014 |  |  | 1 |  |  |  |  |  |  |  | 1 |  |  |  |  |  |  |  |  |  |  |  |  |
| Shahkolahi 2013 |  |  | 1 |  |  |  |  |  |  |  |  |  |  |  |  |  |  |  |  |  |  |  |  |
| Biggs 2016 |  |  |  | 1 |  |  |  |  |  |  |  |  |  |  |  |  |  |  |  |  |  |  |  |
| Bourne 2011 |  |  |  | 1 | 1 |  |  |  |  |  |  | 1 |  |  |  | 1 |  |  | 1 |  |  |  |  |
| Burton 2014 |  |  |  | 1 | 1 |  |  |  |  |  |  |  |  |  |  |  |  |  |  |  |  |  |  |
| Guy 2013 |  |  |  | 1 |  |  |  |  |  |  |  |  |  |  |  |  |  |  |  |  |  |  |  |
| Ingersoll 2015 |  |  |  | 1 |  |  |  |  |  |  |  |  |  | 1 |  |  |  |  |  |  |  |  |  |
| Malotte 2004 |  |  |  | 1 |  |  |  |  |  |  |  |  |  |  |  |  |  |  |  |  |  |  |  |
| Norton 2014 |  |  |  | 1 |  |  |  |  |  |  |  |  |  | 1 |  |  |  |  |  |  |  |  |  |
| Nyatsanza 2016 |  |  |  | 1 |  |  |  |  |  |  |  |  |  |  |  |  |  |  |  |  |  |  |  |
| Rana 2016 |  |  |  | 1 |  |  |  |  |  |  |  |  |  |  |  |  |  |  |  |  |  |  |  |
| Rutland 2012 |  |  |  | 1 |  |  |  |  |  |  |  |  |  | 1 |  |  |  |  |  |  |  |  |  |
| Tanner 2018 |  |  |  | 1 |  |  |  |  |  |  |  |  |  |  |  |  |  |  |  |  |  |  |  |
| Zou 2013 |  |  |  | 1 |  |  |  |  |  |  |  |  |  |  |  |  |  |  | 1 |  |  |  |  |
| Rinehart 2020 |  | 1 |  |  |  |  |  |  |  |  |  |  |  |  | 1 |  |  |  |  |  |  |  |  |
| Agarwal 2015 |  |  |  |  | 1 |  |  |  |  |  |  |  |  |  |  |  |  |  |  |  |  |  |  |
| Odeny 2014 |  |  |  |  | 1 |  |  |  |  |  |  |  |  |  |  |  |  |  |  |  |  |  |  |
| Udeagu 2014 |  |  |  |  | 1 |  |  |  |  |  |  |  |  |  |  |  |  |  |  |  |  |  |  |
| Zou 2013 |  |  |  |  | 1 |  |  |  |  |  |  |  |  |  |  |  |  |  |  |  |  |  |  |
| Bertozzi 2018 |  |  |  |  |  | 1 |  |  |  |  |  |  |  |  |  |  |  |  |  |  |  |  |  |
| Chib 2011 |  |  |  |  |  | 1 |  |  |  |  |  |  |  |  |  |  |  |  |  |  |  |  |  |
| Chu 2015 |  |  |  |  |  | 1 |  |  |  |  |  |  |  |  |  |  | 1 |  |  |  |  |  |  |
| Escobar-Chaves 2011 |  |  |  |  |  | 1 |  |  |  |  |  |  |  |  |  |  |  |  |  |  |  |  |  |
| Gariepy 2018 |  |  |  |  |  | 1 |  |  |  |  |  |  |  |  |  |  |  |  |  |  |  |  |  |
| Haruna 2018 |  |  |  |  |  | 1 |  |  |  |  | 1 |  |  |  |  |  | 1 |  |  |  |  |  |  |
| Markham 2012 |  |  |  |  |  | 1 |  |  |  |  |  |  |  |  |  |  |  |  |  |  |  |  |  |
| Oliveira 2016 |  |  |  |  |  | 1 |  |  |  |  |  |  |  |  |  |  |  |  |  |  |  |  |  |
| Peskin 2015 |  |  |  |  |  | 1 |  |  |  |  |  |  |  |  |  |  |  |  |  |  |  |  | 1 |
| Peskin 2019 |  |  |  |  |  | 1 |  |  |  |  |  |  |  |  |  |  | 1 |  |  |  |  |  |  |
| Potter 2016 |  |  |  |  |  | 1 |  |  |  |  |  |  |  |  |  |  |  |  |  |  |  |  |  |
| Rohrbach 2019 |  |  |  |  |  | 1 |  |  |  |  |  |  |  |  |  |  | 1 |  |  |  |  |  |  |
| Shegog 2014 |  |  |  |  |  | 1 |  |  |  |  |  |  |  |  |  |  |  |  |  |  |  |  |  |
| Shegog 2017 |  |  |  |  |  | 1 |  |  |  |  |  |  |  |  |  |  |  |  |  |  |  |  |  |
| Shegog 2021 |  |  |  |  |  | 1 |  |  |  |  |  |  |  |  |  |  |  |  |  |  |  |  |  |
| Tortolero 2010 |  |  |  |  |  | 1 |  |  |  |  |  |  |  |  |  |  |  |  |  |  |  |  |  |
| Winskell 2018 |  |  |  |  |  | 1 |  |  |  |  | 1 |  |  |  | 1 |  |  |  |  |  |  |  |  |
| Bull 2012 |  |  |  |  |  |  | 1 |  |  |  |  |  |  |  |  |  | 1 |  |  |  |  |  | 1 |
| Danielson 2013 |  |  |  |  |  |  | 1 |  |  |  |  |  |  |  |  |  | 1 |  |  |  |  |  |  |
| Gold 2011 |  |  |  |  |  |  | 1 |  |  |  |  |  |  |  |  |  |  |  |  |  |  |  |  |
| Huang and Hung 2009 |  |  |  |  |  |  | 1 |  |  |  |  |  |  |  |  |  |  |  |  |  |  |  |  |
| Jones 2012 |  |  |  |  |  |  | 1 |  |  |  |  |  |  |  |  |  |  |  |  |  |  |  |  |
| Juzang 2011 |  |  |  |  |  |  | 1 |  |  |  |  |  |  |  |  |  | 1 |  |  |  |  |  |  |
| Markham 2014 |  |  |  |  |  |  | 1 |  |  |  |  |  |  |  |  |  |  |  |  |  |  |  |  |
| Roberto 2007 |  |  |  |  |  |  | 1 |  |  |  |  |  |  |  |  |  |  |  |  |  |  |  |  |
| Van den Elshout MAM 2021 |  |  |  |  |  |  |  | 1 |  |  |  |  |  |  |  |  |  |  |  |  |  |  |  |
| Colson 2020 |  |  |  |  |  |  |  | 1 |  |  |  |  |  |  |  |  |  |  |  |  |  |  |  |
| Moore Dj 2018 |  |  |  |  |  |  |  | 1 |  |  |  | 1 |  |  |  |  |  |  |  |  |  |  |  |
| Fuchs JD 2018 |  |  |  |  |  |  |  | 1 |  |  |  | 1 |  |  |  |  |  |  |  |  |  |  |  |
| Mitchell JT 2018 |  |  |  |  |  |  |  | 1 |  |  |  |  |  |  |  |  |  |  |  |  |  |  |  |
| Liu 2019 |  |  |  |  |  |  |  | 1 |  |  |  |  |  |  |  |  | 1 |  |  | 1 |  | 1 |  |
| Whiteley 2021 |  |  |  |  |  |  |  | 1 |  |  |  |  |  |  |  |  |  |  |  | 1 |  | 1 |  |
| Mitchel JT 2022 |  |  |  |  |  |  |  | 1 |  |  |  |  |  |  |  |  |  |  |  |  |  |  |  |
| Weitzman PF 2021 |  |  |  |  |  |  |  | 1 |  |  |  |  |  |  |  |  |  |  |  |  |  |  |  |
| Gerend 2021 |  |  |  |  |  |  |  |  | 1 |  |  |  |  |  |  |  |  |  |  |  |  |  |  |
| Tull 2019 |  |  |  |  |  |  |  |  | 1 |  |  |  | 1 |  |  |  |  |  |  |  |  |  |  |
| Keeshin & Feinberg 2017 |  |  |  |  |  |  |  |  | 1 |  |  |  |  |  |  |  |  |  |  |  |  |  |  |
| Rand 2017 |  |  |  |  |  |  |  |  | 1 |  |  |  |  |  |  |  |  |  |  |  |  |  |  |
| Rand 2015 |  |  |  |  |  |  |  |  | 1 |  |  |  | 1 |  |  |  |  |  |  |  |  |  |  |
| Matheson 2014 |  |  |  |  |  |  |  |  | 1 |  |  |  |  |  |  |  |  |  |  |  |  |  |  |
| Kharbanda 2011 |  |  |  |  |  |  |  |  | 1 |  |  |  |  |  |  |  |  |  |  |  |  |  |  |
| Bowen 2008 |  |  |  |  |  |  |  |  |  | 1 |  | 1 |  |  |  | 1 |  |  | 1 |  |  |  |  |
| Lau 2008 |  |  |  |  |  |  |  |  |  | 1 |  | 1 |  |  |  | 1 |  |  | 1 |  |  |  |  |
| Blas 2010 |  |  |  |  |  |  |  |  |  | 1 |  | 1 |  |  |  | 1 |  | 1 |  |  |  |  |  |
| Mustanski 2013 |  |  |  |  |  |  |  |  |  | 1 |  |  |  |  |  | 1 |  |  | 1 |  |  |  | 1 |
| Kasatpibal 2014 |  |  |  |  |  |  |  |  |  | 1 |  | 1 |  |  |  |  |  |  | 1 |  |  |  |  |
| Mustanski 2014 |  |  |  |  |  |  |  |  |  | 1 |  |  |  |  |  |  |  |  |  |  |  |  |  |
| Lelutiu-Weinberger 2015 |  |  |  |  |  |  |  |  |  | 1 |  | 1 |  |  |  |  |  |  | 1 |  |  |  |  |
| Mustanski 2015 |  |  |  |  |  |  |  |  |  | 1 |  |  |  |  |  |  |  |  |  |  |  |  |  |
| Young SD 2015 |  |  |  |  |  |  |  |  |  | 1 |  | 1 |  | 1 |  |  |  | 1 | 1 |  |  |  |  |
| Huang 2016 |  |  |  |  |  |  |  |  |  | 1 |  |  |  |  |  |  |  |  |  |  |  |  |  |
| Lau 2016 |  |  |  |  |  |  |  |  |  | 1 |  | 1 |  |  |  |  |  |  | 1 |  | 1 |  | 1 |
| Solorio 2016 |  |  |  |  |  |  |  |  |  | 1 |  | 1 |  |  |  |  | 1 |  | 1 |  |  |  |  |
| Barnabas 2016 |  |  |  |  |  |  |  |  |  |  | 1 |  |  | 1 |  |  |  |  |  |  |  |  |  |
| Haberer 2016 |  |  |  |  |  |  |  |  |  |  | 1 |  |  |  |  |  |  |  |  |  |  |  |  |
| Harder 2019 |  |  |  |  |  |  |  |  |  |  | 1 |  |  |  |  |  |  |  |  |  |  |  |  |
| Joseph Davey 2016 |  |  |  |  |  |  |  |  |  |  | 1 |  |  | 1 |  |  |  |  |  |  |  |  |  |
| Kalichman 2019 |  |  |  |  |  |  |  |  |  |  | 1 |  |  |  |  |  |  |  |  |  |  |  |  |
| Kiwanuka 2018 |  |  |  |  |  |  |  |  |  |  | 1 |  |  |  |  |  |  |  |  |  |  |  |  |
| Kurth 2019 |  |  |  |  |  |  |  |  |  |  | 1 |  |  |  |  |  |  |  |  |  |  |  |  |
| Lapinski 2008 |  |  |  |  |  |  |  |  |  |  | 1 |  |  |  |  |  |  |  |  |  |  |  |  |
| Lester 2010 |  |  |  |  |  |  |  |  |  |  | 1 |  |  | 1 |  |  |  |  |  |  |  |  |  |
| Linnemayr 2017 |  |  |  |  |  |  |  |  |  |  | 1 |  |  |  |  |  | 1 |  |  |  |  |  |  |
| MacCarthy 2020 |  |  |  |  |  |  |  |  |  |  | 1 |  |  |  |  |  |  |  |  |  |  |  |  |
| Mbuagbaw 2012 |  |  |  |  |  |  |  |  |  |  | 1 |  |  | 1 |  |  |  |  |  |  |  |  |  |
| Nsagha 2016 |  |  |  |  |  |  |  |  |  |  | 1 |  |  | 1 |  |  |  |  |  |  |  |  |  |
| Pop-Eleches 2011 |  |  |  |  |  |  |  |  |  |  | 1 |  |  |  |  |  |  |  |  |  |  |  |  |
| Reid 2017 |  |  |  |  |  |  |  |  |  |  | 1 |  |  |  |  |  |  |  |  |  |  |  |  |
| Van der Kop 2018 |  |  |  |  |  |  |  |  |  |  | 1 |  |  |  |  |  |  |  |  |  |  |  |  |
| Venter 2019 |  |  |  |  |  |  |  |  |  |  | 1 |  |  |  |  |  |  |  |  |  |  |  |  |
| Ybarra 2015 |  |  |  |  |  |  |  |  |  |  | 1 |  |  |  |  |  |  |  |  |  |  |  | 1 |
| DiClemente 2015 |  |  |  |  |  |  |  |  |  |  |  |  | 1 |  |  |  |  |  |  |  |  |  |  |
| Chen 2017 |  |  |  |  |  |  |  |  |  |  |  |  | 1 |  |  |  |  |  |  |  |  |  |  |
| Pot 2017 |  |  |  |  |  |  |  |  |  |  |  |  | 1 |  |  |  |  |  |  |  |  |  |  |
| Hofstetter 2017 |  |  |  |  |  |  |  |  |  |  |  |  | 1 |  |  |  |  |  |  |  |  |  |  |
| Mohanty 2018 |  |  |  |  |  |  |  |  |  |  |  |  | 1 |  |  |  |  |  |  |  |  |  |  |
| Ortiz 2018 |  |  |  |  |  |  |  |  |  |  |  |  | 1 |  |  |  |  |  |  |  |  |  |  |
| Chen, Todd et al., 2019 |  |  |  |  |  |  |  |  |  |  |  |  | 1 |  |  |  |  |  |  |  |  |  |  |
| Chen 2019 |  |  |  |  |  |  |  |  |  |  |  |  | 1 |  |  |  |  |  |  |  |  |  |  |
| Dempsey 2019 |  |  |  |  |  |  |  |  |  |  |  |  | 1 |  |  |  |  |  |  |  |  |  |  |
| Dixon 2019 |  |  |  |  |  |  |  |  |  |  |  |  | 1 |  |  |  |  |  |  |  |  |  |  |
| Panozzo 2020 |  |  |  |  |  |  |  |  |  |  |  |  | 1 |  |  |  |  |  |  |  |  |  |  |
| Szilagyi 2020 |  |  |  |  |  |  |  |  |  |  |  |  | 1 |  |  |  |  |  |  |  |  |  |  |
| Suzuki 2021 |  |  |  |  |  |  |  |  |  |  |  |  | 1 |  |  |  |  |  |  |  |  |  |  |
| Becker 2022 |  |  |  |  |  |  |  |  |  |  |  |  | 1 |  |  |  |  |  |  |  |  |  |  |
| Shegog 2022 |  |  |  |  |  |  |  |  |  |  |  |  | 1 |  |  |  |  |  |  |  |  |  |  |
| Chiasson 2009 |  |  |  |  |  |  |  |  |  |  |  | 1 |  |  |  |  |  |  | 1 |  |  |  |  |
| Rhodes 2011 |  |  |  |  |  |  |  |  |  |  |  | 1 |  |  |  | 1 |  | 1 | 1 |  |  |  |  |
| Sullivan 2017 |  |  |  |  |  |  |  |  |  |  |  | 1 |  |  |  |  |  |  |  |  |  |  |  |
| Reback 2012 |  |  |  |  |  |  |  |  |  |  |  | 1 |  |  |  | 1 |  |  | 1 |  |  |  |  |
| Reiter 2018 |  |  |  |  |  |  |  |  |  |  |  | 1 |  |  |  |  |  |  |  |  |  |  |  |
| Wray 2019 |  |  |  |  |  |  |  |  |  |  |  | 1 |  |  |  |  |  |  |  |  |  |  | 1 |
| Bauermeister 2019 |  |  |  |  |  |  |  |  |  |  |  | 1 |  |  |  |  |  |  |  |  |  |  |  |
| Starks 2019 |  |  |  |  |  |  |  |  |  |  |  | 1 |  |  |  |  |  |  |  |  |  |  |  |
| Clark 2018 |  |  |  |  |  |  |  |  |  |  |  | 1 |  |  |  |  |  |  |  |  |  |  |  |
| McRee 2018 |  |  |  |  |  |  |  |  |  |  |  | 1 |  |  | 1 |  |  |  |  |  |  |  |  |
| Adam 2011 |  |  |  |  |  |  |  |  |  |  |  | 1 |  |  |  |  |  |  |  |  |  |  |  |
| Greene 2016 |  |  |  |  |  |  |  |  |  |  |  | 1 |  |  |  |  |  |  | 1 |  |  |  |  |
| Bachmann 2013 |  |  |  |  |  |  |  |  |  |  |  | 1 |  |  |  |  |  |  |  |  |  |  |  |
| Prati 2016 |  |  |  |  |  |  |  |  |  |  |  | 1 |  |  |  |  |  |  | 1 |  |  |  |  |
| Ko 2013 |  |  |  |  |  |  |  |  |  |  |  | 1 |  |  |  | 1 |  | 1 | 1 |  | 1 |  |  |
| Klein 2017 |  |  |  |  |  |  |  |  |  |  |  | 1 |  |  |  |  |  |  |  |  |  |  |  |
| Mi 2015 |  |  |  |  |  |  |  |  |  |  |  | 1 |  |  |  |  |  |  | 1 |  |  |  |  |
| Mclver 2016 |  |  |  |  |  |  |  |  |  |  |  | 1 |  |  |  |  |  |  |  |  |  |  |  |
| Rhodes 2016 |  |  |  |  |  |  |  |  |  |  |  | 1 |  |  |  |  |  | 1 | 1 |  |  |  |  |
| Lin 2012 |  |  |  |  |  |  |  |  |  |  |  | 1 |  |  |  |  |  |  |  |  |  |  |  |
| Fleming 2017 |  |  |  |  |  |  |  |  |  |  |  | 1 |  |  |  |  |  |  |  |  |  |  |  |
| Noslinger 2016 |  |  |  |  |  |  |  |  |  |  |  | 1 |  |  |  |  |  |  |  |  |  |  |  |
| Hirshfield 2012 |  |  |  |  |  |  |  |  |  |  |  | 1 |  |  |  | 1 |  | 1 | 1 |  |  |  |  |
| Hightow-Weidman 2019 |  |  |  |  |  |  |  |  |  |  |  | 1 |  |  |  |  |  |  |  |  |  |  | 1 |
| Millard 2016 |  |  |  |  |  |  |  |  |  |  |  | 1 |  |  |  |  |  |  |  |  |  |  |  |
| Tang 2016 |  |  |  |  |  |  |  |  |  |  |  | 1 |  |  |  |  |  | 1 |  |  | 1 |  |  |
| Young 2014 |  |  |  |  |  |  |  |  |  |  |  | 1 |  |  |  |  |  |  |  |  |  |  |  |
| Fernandez 2016 |  |  |  |  |  |  |  |  |  |  |  | 1 |  |  |  |  |  |  | 1 |  |  |  |  |
| Kerani 2011 |  |  |  |  |  |  |  |  |  |  |  | 1 |  |  |  |  |  |  |  |  |  |  |  |
| Wang 2017 |  |  |  |  |  |  |  |  |  |  |  | 1 |  |  |  |  |  | 1 | 1 |  | 1 |  |  |
| Smith 2015 |  |  |  |  |  |  |  |  |  |  |  | 1 |  |  |  |  |  |  |  |  |  |  |  |
| Cruess 2018 |  |  |  |  |  |  |  |  |  |  |  | 1 |  |  |  |  |  |  |  |  |  |  |  |
| Mustanski 2018 |  |  |  |  |  |  |  |  |  |  |  | 1 |  |  |  |  |  |  | 1 |  |  |  | 1 |
| Abdul Rashid 2013 |  |  |  |  |  |  |  |  |  |  |  |  |  | 1 |  |  |  |  |  |  |  |  |  |
| Belzer 2015 |  |  |  |  |  |  |  |  |  |  |  |  |  | 1 |  |  |  |  |  |  |  |  |  |
| Bull 2016 |  |  |  |  |  |  |  |  |  |  |  |  |  | 1 |  |  |  |  |  |  |  |  |  |
| Castano 2012 |  |  |  |  |  |  |  |  |  |  |  |  |  | 1 |  |  |  |  |  |  |  |  |  |
| Constant 2014 |  |  |  |  |  |  |  |  |  |  |  |  |  | 1 |  |  |  |  |  |  |  |  |  |
| Cook 2015 |  |  |  |  |  |  |  |  |  |  |  |  |  | 1 |  |  |  |  |  |  |  |  |  |
| da Costa 2012 |  |  |  |  |  |  |  |  |  |  |  |  |  | 1 |  |  |  |  |  |  |  |  |  |
| Garofalo 2016 |  |  |  |  |  |  |  |  |  |  |  |  |  | 1 |  |  |  |  |  |  |  |  |  |
| Gerdts 2015 |  |  |  |  |  |  |  |  |  |  |  |  |  | 1 |  |  |  |  |  |  |  |  |  |
| Hou 2010 |  |  |  |  |  |  |  |  |  |  |  |  |  | 1 |  |  |  |  |  |  |  |  |  |
| Huang 2013 |  |  |  |  |  |  |  |  |  |  |  |  |  | 1 |  |  |  |  |  |  |  |  |  |
| Jeffries 2016 |  |  |  |  |  |  |  |  |  |  |  |  |  | 1 |  |  |  |  |  |  |  |  |  |
| Lee 2016 |  |  |  |  |  |  |  |  |  |  |  |  |  | 1 |  |  |  |  |  |  |  |  |  |
| McCarthy 2016 |  |  |  |  |  |  |  |  |  |  |  |  |  | 1 |  |  | 1 |  |  |  |  |  |  |
| Pop‐Eleches 2011 |  |  |  |  |  |  |  |  |  |  |  |  |  | 1 |  |  |  |  |  |  |  |  |  |
| Reed 2014 |  |  |  |  |  |  |  |  |  |  |  |  |  | 1 |  |  |  |  |  |  |  |  |  |
| Ruan 2017 |  |  |  |  |  |  |  |  |  |  |  |  |  | 1 |  |  |  |  |  |  |  |  |  |
| Russell 2012 |  |  |  |  |  |  |  |  |  |  |  |  |  | 1 |  |  |  |  |  |  |  |  |  |
| Shet 2014 |  |  |  |  |  |  |  |  |  |  |  |  |  | 1 |  |  |  |  |  |  |  |  |  |
| Smith 2015 |  |  |  |  |  |  |  |  |  |  |  |  |  | 1 |  |  |  |  |  |  |  |  |  |
| Bannink 2014 |  |  |  |  |  |  |  |  |  |  |  |  |  |  | 1 |  |  |  |  |  |  |  |  |
| Cordova 2020 |  |  |  |  |  |  |  |  |  |  |  |  |  |  | 1 |  |  |  |  |  |  |  | 1 |
| McCarthy 2020 |  |  |  |  |  |  |  |  |  |  |  |  |  |  | 1 |  | 1 |  |  |  |  |  |  |
| McCarthy 2019 |  |  |  |  |  |  |  |  |  |  |  |  |  |  | 1 |  |  |  |  |  |  |  |  |
| Saberi 2021 |  |  |  |  |  |  |  |  |  |  |  |  |  |  | 1 |  |  |  |  |  |  |  |  |
| Scull TM 2018 |  |  |  |  |  |  |  |  |  |  |  |  |  |  | 1 |  | 1 |  |  |  |  |  |  |
| Starosta 2016 |  |  |  |  |  |  |  |  |  |  |  |  |  |  | 1 |  |  |  |  |  |  |  |  |
| Stephenson 2020 |  |  |  |  |  |  |  |  |  |  |  |  |  |  | 1 |  |  |  |  |  |  |  |  |
| Widman 2018 |  |  |  |  |  |  |  |  |  |  |  |  |  |  | 1 |  | 1 |  |  |  |  |  | 1 |
| Wong 2021 |  |  |  |  |  |  |  |  |  |  |  |  |  |  | 1 |  |  |  |  |  |  |  |  |
| Belzer 2014 |  |  |  |  |  |  |  |  |  |  |  |  |  |  |  |  | 1 |  |  |  |  |  |  |
| Bull 2017 |  |  |  |  |  |  |  |  |  |  |  |  |  |  |  |  | 1 |  |  |  |  |  |  |
| Hacking 2019 |  |  |  |  |  |  |  |  |  |  |  |  |  |  |  |  | 1 |  |  |  |  |  |  |
| John 2016 |  |  |  |  |  |  |  |  |  |  |  |  |  |  |  |  | 1 |  |  |  |  |  |  |
| Merril 2018 |  |  |  |  |  |  |  |  |  |  |  |  |  |  |  |  | 1 |  |  |  |  |  |  |
| Rockicki 2017 |  |  |  |  |  |  |  |  |  |  |  |  |  |  |  |  | 1 |  |  |  |  |  |  |
| Stankievich 2018 |  |  |  |  |  |  |  |  |  |  |  |  |  |  |  |  | 1 |  |  |  |  |  |  |
| Yao 2018 |  |  |  |  |  |  |  |  |  |  |  |  |  |  |  |  | 1 |  |  |  |  |  |  |
| Ybarra 2018 |  |  |  |  |  |  |  |  |  |  |  |  |  |  |  |  | 1 |  |  |  |  |  |  |
| Brayboy 2017 |  |  |  |  |  |  |  |  |  |  |  |  |  |  |  |  | 1 |  |  |  |  |  |  |
| Dehghani 2016 |  |  |  |  |  |  |  |  |  |  |  |  |  |  |  |  | 1 |  |  |  |  |  |  |
| Jeong 2017 |  |  |  |  |  |  |  |  |  |  |  |  |  |  |  |  | 1 |  |  |  |  |  |  |
| Manlove 2020 |  |  |  |  |  |  |  |  |  |  |  |  |  |  |  |  | 1 |  |  |  |  |  |  |
| Mesheriakova 2017 |  |  |  |  |  |  |  |  |  |  |  |  |  |  |  |  | 1 |  |  |  |  |  |  |
| Nielsen 2019 |  |  |  |  |  |  |  |  |  |  |  |  |  |  |  |  | 1 |  |  |  |  |  |  |
| Bailey 2013 |  |  |  |  |  |  |  |  |  |  |  |  |  |  |  |  | 1 |  |  |  |  |  |  |
| Ballester-Arnal 2015 |  |  |  |  |  |  |  |  |  |  |  |  |  |  |  |  | 1 |  |  |  |  |  |  |
| Brown 2016 |  |  |  |  |  |  |  |  |  |  |  |  |  |  |  |  | 1 |  |  |  |  |  |  |
| Doubova 2017 |  |  |  |  |  |  |  |  |  |  |  |  |  |  |  |  | 1 |  |  |  |  |  |  |
| Gottvall 2010 |  |  |  |  |  |  |  |  |  |  |  |  |  |  |  |  | 1 |  |  |  |  |  |  |
| Horvath 2017 |  |  |  |  |  |  |  |  |  |  |  |  |  |  |  |  | 1 |  |  |  |  |  |  |
| Lustria 2016 |  |  |  |  |  |  |  |  |  |  |  |  |  |  |  |  | 1 |  |  |  |  |  |  |
| Massey 2013 |  |  |  |  |  |  |  |  |  |  |  |  |  |  |  |  | 1 |  |  |  |  |  |  |
| Mevissen 2011 |  |  |  |  |  |  |  |  |  |  |  |  |  |  |  |  | 1 |  |  |  |  |  |  |
| Mortimer 2015 |  |  |  |  |  |  |  |  |  |  |  |  |  |  |  |  | 1 |  |  |  |  |  |  |
| Naar-King 2013 |  |  |  |  |  |  |  |  |  |  |  |  |  |  |  |  | 1 |  |  |  |  |  |  |
| Spielberg 2014 |  |  |  |  |  |  |  |  |  |  |  |  |  |  |  |  | 1 |  |  |  |  |  |  |
| Starling 2014 |  |  |  |  |  |  |  |  |  |  |  |  |  |  |  |  | 1 |  |  |  |  |  |  |
| Villegas 2015 |  |  |  |  |  |  |  |  |  |  |  |  |  |  |  |  | 1 |  |  |  |  |  |  |
| Carvalho 2016 |  |  |  |  |  |  |  |  |  |  |  |  |  |  |  |  | 1 |  |  |  |  |  |  |
| Castillo-Arcos 2016 |  |  |  |  |  |  |  |  |  |  |  |  |  |  |  |  | 1 |  |  |  |  |  |  |
| Chong 2020 |  |  |  |  |  |  |  |  |  |  |  |  |  |  |  |  | 1 |  |  |  |  |  |  |
| Kaufman 2018 |  |  |  |  |  |  |  |  |  |  |  |  |  |  |  |  | 1 |  |  |  |  |  |  |
| Klein 2017 |  |  |  |  |  |  |  |  |  |  |  |  |  |  |  |  | 1 |  |  |  |  |  |  |
| Shafii 2019 |  |  |  |  |  |  |  |  |  |  |  |  |  |  |  |  | 1 |  |  |  |  |  |  |
| Markham 2020 |  |  |  |  |  |  |  |  |  |  |  |  |  |  |  |  | 1 |  |  |  |  |  |  |
| Dulli 2020 |  |  |  |  |  |  |  |  |  |  |  |  |  |  |  |  | 1 |  |  |  |  |  |  |
| Whiteley 2018 |  |  |  |  |  |  |  |  |  |  |  |  |  |  |  |  | 1 |  |  |  |  |  |  |
| Ezegbe 2018 |  |  |  |  |  |  |  |  |  |  |  |  |  |  |  |  | 1 |  |  |  |  |  | 1 |
| Sznitman 2010 |  |  |  |  |  |  |  |  |  |  |  |  |  |  |  |  | 1 |  |  |  |  |  |  |
| Blas 2014 |  |  |  |  |  |  |  |  |  |  |  |  |  |  |  |  |  | 1 |  |  |  |  |  |
| Patel 2016 |  |  |  |  |  |  |  |  |  |  |  |  |  |  |  |  |  | 1 | 1 |  |  |  |  |
| Washington 2017 |  |  |  |  |  |  |  |  |  |  |  |  |  |  |  |  |  | 1 |  |  |  |  |  |
| Anand 2018 |  |  |  |  |  |  |  |  |  |  |  |  |  |  |  |  |  |  | 1 |  |  |  | 1 |
| Christensen 2007 |  |  |  |  |  |  |  |  |  |  |  |  |  |  |  |  |  |  | 1 |  |  |  |  |
| Desai 2014 |  |  |  |  |  |  |  |  |  |  |  |  |  |  |  |  |  |  | 1 |  |  |  |  |
| Habarta 2017 |  |  |  |  |  |  |  |  |  |  |  |  |  |  |  |  |  |  | 1 |  |  |  |  |
| Hilliam 2011 |  |  |  |  |  |  |  |  |  |  |  |  |  |  |  |  |  |  | 1 |  |  |  |  |
| Lelutiu-Weinberger 2018 |  |  |  |  |  |  |  |  |  |  |  |  |  |  |  |  |  |  | 1 |  |  |  |  |
| Mikolajczak 2013 |  |  |  |  |  |  |  |  |  |  |  |  |  |  |  |  |  |  | 1 |  |  |  |  |
| Nöstlinger 2016 |  |  |  |  |  |  |  |  |  |  |  |  |  |  |  |  |  |  | 1 |  |  |  |  |
| Uhrig 2012 |  |  |  |  |  |  |  |  |  |  |  |  |  |  |  |  |  |  | 1 |  |  |  |  |
| Buchbinder 2023 |  |  |  |  |  |  |  |  |  |  |  |  |  |  |  |  |  |  |  | 1 |  |  |  |
| Erenrich 2024 |  |  |  |  |  |  |  |  |  |  |  |  |  |  |  |  |  |  |  | 1 |  | 1 |  |
| Liu 2021 |  |  |  |  |  |  |  |  |  |  |  |  |  |  |  |  |  |  |  | 1 |  |  |  |
| Schnall 2022 |  |  |  |  |  |  |  |  |  |  |  |  |  |  |  |  |  |  |  | 1 |  | 1 | 1 |
| Schneider 2021 |  |  |  |  |  |  |  |  |  |  |  |  |  |  |  |  |  |  |  | 1 |  |  |  |
| Serrano 2023 |  |  |  |  |  |  |  |  |  |  |  |  |  |  |  |  |  |  |  | 1 |  |  |  |
| Sullivan 2022 |  |  |  |  |  |  |  |  |  |  |  |  |  |  |  |  |  |  |  | 1 |  | 1 |  |
| Biello 2022 |  |  |  |  |  |  |  |  |  |  |  |  |  |  |  |  |  |  |  | 1 |  |  |  |
| Li 2023 |  |  |  |  |  |  |  |  |  |  |  |  |  |  |  |  |  |  |  | 1 |  |  |  |
| Wray 2024 |  |  |  |  |  |  |  |  |  |  |  |  |  |  |  |  |  |  |  | 1 |  |  |  |
| Cheng 2019 |  |  |  |  |  |  |  |  |  |  |  |  |  |  |  |  |  |  |  |  | 1 |  |  |
| Chiou 2020 |  |  |  |  |  |  |  |  |  |  |  |  |  |  |  |  |  |  |  |  | 1 |  |  |
| Yun 2021 |  |  |  |  |  |  |  |  |  |  |  |  |  |  |  |  |  |  |  |  | 1 |  |  |
| Li 2020 |  |  |  |  |  |  |  |  |  |  |  |  |  |  |  |  |  |  |  |  | 1 |  |  |
| Xiao 2020 |  |  |  |  |  |  |  |  |  |  |  |  |  |  |  |  |  |  |  |  | 1 |  |  |
| Luo 2023 |  |  |  |  |  |  |  |  |  |  |  |  |  |  |  |  |  |  |  |  | 1 |  |  |
| Tang 2019 |  |  |  |  |  |  |  |  |  |  |  |  |  |  |  |  |  |  |  |  | 1 |  |  |
| Liu 2014 |  |  |  |  |  |  |  |  |  |  |  |  |  |  |  |  |  |  |  |  | 1 |  |  |
| Song 2017 |  |  |  |  |  |  |  |  |  |  |  |  |  |  |  |  |  |  |  |  | 1 |  |  |
| Tao 2020 |  |  |  |  |  |  |  |  |  |  |  |  |  |  |  |  |  |  |  |  | 1 |  |  |
| Yan 2013 |  |  |  |  |  |  |  |  |  |  |  |  |  |  |  |  |  |  |  |  | 1 |  |  |
| Zhang 2014 |  |  |  |  |  |  |  |  |  |  |  |  |  |  |  |  |  |  |  |  | 1 |  |  |
| Liu 2012 |  |  |  |  |  |  |  |  |  |  |  |  |  |  |  |  |  |  |  |  | 1 |  |  |
| Wang FX 2011 |  |  |  |  |  |  |  |  |  |  |  |  |  |  |  |  |  |  |  |  | 1 |  |  |
| Wang XD 2014 |  |  |  |  |  |  |  |  |  |  |  |  |  |  |  |  |  |  |  |  | 1 |  |  |
| Wang Y 2009 |  |  |  |  |  |  |  |  |  |  |  |  |  |  |  |  |  |  |  |  | 1 |  |  |
| Xie 2018 |  |  |  |  |  |  |  |  |  |  |  |  |  |  |  |  |  |  |  |  | 1 |  |  |
| Moore 2018 |  |  |  |  |  |  |  |  |  |  |  |  |  |  |  |  |  |  |  |  |  | 1 |  |
| Bauermeister 2019 |  |  |  |  |  |  |  |  |  |  |  |  |  |  |  |  |  |  |  |  |  | 1 | 1 |
| Njuguna 2019 |  |  |  |  |  |  |  |  |  |  |  |  |  |  |  |  |  |  |  |  |  | 1 |  |
| Songtaweesin 2020 |  |  |  |  |  |  |  |  |  |  |  |  |  |  |  |  |  |  |  |  |  | 1 |  |
| Haberer 2021 |  |  |  |  |  |  |  |  |  |  |  |  |  |  |  |  |  |  |  |  |  | 1 |  |
| Lin 2023 |  |  |  |  |  |  |  |  |  |  |  |  |  |  |  |  |  |  |  |  |  | 1 |  |
| Wray 2022 |  |  |  |  |  |  |  |  |  |  |  |  |  |  |  |  |  |  |  |  |  | 1 |  |
| Horvath 2024 |  |  |  |  |  |  |  |  |  |  |  |  |  |  |  |  |  |  |  |  |  | 1 |  |
| Biello 2025 |  |  |  |  |  |  |  |  |  |  |  |  |  |  |  |  |  |  |  |  |  | 1 |  |
| Brody 2022 |  |  |  |  |  |  |  |  |  |  |  |  |  |  |  |  |  |  |  |  |  |  | 1 |
| Levy 2021 |  |  |  |  |  |  |  |  |  |  |  |  |  |  |  |  |  |  |  |  |  |  | 1 |
| Logie 2023 |  |  |  |  |  |  |  |  |  |  |  |  |  |  |  |  |  |  |  |  |  |  | 1 |
| McCrimmon 2024 |  |  |  |  |  |  |  |  |  |  |  |  |  |  |  |  |  |  |  |  |  |  | 1 |
| Nelson 2022 |  |  |  |  |  |  |  |  |  |  |  |  |  |  |  |  |  |  |  |  |  |  | 1 |
| Newcomb 2022 |  |  |  |  |  |  |  |  |  |  |  |  |  |  |  |  |  |  |  |  |  |  | 1 |
| Santa Maria 2021 |  |  |  |  |  |  |  |  |  |  |  |  |  |  |  |  |  |  |  |  |  |  | 1 |
| Swendeman 2024 |  |  |  |  |  |  |  |  |  |  |  |  |  |  |  |  |  |  |  |  |  |  | 1 |
| Widman 2020 |  |  |  |  |  |  |  |  |  |  |  |  |  |  |  |  |  |  |  |  |  |  | 1 |
| Yi 2024 |  |  |  |  |  |  |  |  |  |  |  |  |  |  |  |  |  |  |  |  |  |  | 1 |
| Zhang 2024 |  |  |  |  |  |  |  |  |  |  |  |  |  |  |  |  |  |  |  |  |  |  | 1 |
