# Supplementary material for: Behavioral Determinants and Effectiveness of Digital Behavior Change Interventions for the Prevention of Sexually Transmitted Infections and HIV: Overview of Systematic Reviews
Source: J Med Internet Res. 2026 Jan 29;28:e74201. doi: 10.2196/74201 (PMC12902757; doi:10.2196/74201)
Supplement: Multimedia Appendix 3 [file jmir_v28i1e74201_app3.docx]

**Appendix 3**

**PRISMA-S Checklist for Reporting Literature Searches**

| **Section / Topic** | **#** | **Checklist Item** | **Reported Location in Manuscript** |
| --- | --- | --- | --- |
| **INFORMATION SOURCES AND METHODS** |  |  |  |
| Database name | 1 | Name each individual database searched, stating the platform for each. | Methods → Information Sources (“MEDLINE via PubMed, Cochrane Database of Systematic Reviews, Epistemonikos, PsycINFO; each searched independently using native platforms”) |
| Multi-database searching | 2 | If databases were searched simultaneously on a single platform, state the name of the platform. | Methods → Information Sources (“no multi-database platform such as EBSCOhost or Ovid was used”) |
| Study registries | 3 | List any study registries searched. | NA – Not Applicable (overview of systematic reviews; reported explicitly in Information Sources) |
| Online resources and browsing | 4 | Describe any online or print source browsed (e.g., websites), and how this was done. | Methods → Information Sources; Supplementary Appendix 4 (list of 7 websites, 1 organization; browsing procedures described) |
| Citation searching | 5 | Indicate whether cited/citing references were examined and the methods used. | Methods → Information Sources (“backward citation screening and forward citation tracking using Google Scholar”); Supplementary Appendix 4 |
| Contacts | 6 | Indicate whether additional studies or data were sought by contacting authors or experts. | Methods → Study Selection Process (“28 authors contacted, up to three attempts”); Supplementary Appendix 4 |
| Other methods | 7 | Describe any additional methods used. | Methods → Information Sources (“No automated alerts, web-scraping tools, or API-based retrieval were used; PRISMA-S Not Applicable”) |
| **SEARCH STRATEGIES** |  |  |  |
| Full search strategies | 8 | Include full search strategies for each database and information source, copied exactly as run. | Supplementary Appendices 5–6 (“full strategies line-by-line and block format, copied verbatim as executed”) |
| Limits and restrictions | 9 | Specify any limits or justify when none used. | Methods → Search Strategy (“no restrictions on date, language, or country; no methodological filters applied”) |
| Search filters | 10 | Indicate whether published search filters were used. | Methods → Search Strategy (“No methodological search filters such as systematic review filters or RCT filters were applied”) |
| Prior work | 11 | Indicate whether strategies were adapted or reused from previous reviews. | Methods → Search Strategy (“strategy newly developed; not adapted from prior reviews”) |
| Updates | 12 | Report methods used to update searches. | Methods → Search Strategy (“search rerun on 12 November 2025 following PRISMA-S update guidance”) |
| Dates of searches | 13 | Report the date when each search occurred. | Methods → Search Strategy (“initial search 31 August 2024; rerun 12 November 2025”); Supplementary Appendices 5–6 |
| **PEER REVIEW** |  |  |  |
| Peer review | 14 | Describe any search peer review process. | Methods → Search Strategy (“strategy did not undergo formal PRESS review; Not Applicable”) |
| **MANAGING RECORDS** |  |  |  |
| Total records | 15 | Document number of records identified from each database and information source. | Methods → Information Sources (PubMed=1113; Cochrane=148; PsycINFO=161; Epistemonikos=1891; other sources listed); Supplementary Appendix 6; Figure 1 |
| Deduplication | 16 | Describe software/process used for deduplication. | Methods → Study Selection Process (“Collaboratron automated similarity detection + manual verification by two reviewers”) |
|  |  |  |  |
| PRISMA-S: An Extension to the PRISMA Statement for Reporting Literature Searches in Systematic Reviews Rethlefsen ML, Kirtley S, Waffenschmidt S, Ayala AP, Moher D, Page MJ, Koffel JB, PRISMA-S Group.Last updated February 27, 2020 | | | |
